# Beyond Single‐Active Sites: The Emergence of High‐Entropy Perovskites in Energy and Environment Catalysis

**DOI:** 10.1002/cssc.70851

**Published:** 2026-07-02

**Authors:** K. Aravinthkumar, Pei‐Ying Lin, Shu‐Ling Hsieh, Shuchen Hsieh

**Affiliations:** ^1^ Department of Seafood Science National Kaohsiung University of Science and Technology Kaohsiung Taiwan; ^2^ Department of Chemistry National Sun Yat‐sen University Kaohsiung Taiwan; ^3^ Center for Green‐Energy Key Materials Research National Sun Yat‐Sen University Kaohsiung Taiwan; ^4^ School of Pharmacy College of Pharmacy Kaohsiung Medical University Kaohsiung Taiwan; ^5^ Regenerative Medicine and Cell Therapy Research Center Kaohsiung Medical University Kaohsiung Taiwan; ^6^ Institute of Aquatic Science and Technology College of Hydrosphere Science National Kaohsiung University of Science and Technology Kaohsiung Taiwan

**Keywords:** catalysis, configuration entropy, environmental science, materials science, nanotechnology, perovskite, scalability, sustainable energy

## Abstract

High‐entropy perovskites (HEPs) have emerged as a transformative class of multicomponent oxides that extend beyond the limitations of conventional single‐active‐site perovskites for sustainable energy and environmental catalysis. Configurational entropy provides single‐phase structures by incorporating five or more cations into the ABO_3_ lattice, creating a wide range of local coordination environments, tunable electronic structures, and synergistic catalytic sites. This review summarizes recent advances in entropy engineering strategies, including A‐site, B‐site, and dual‐site disorder, alongside key thermodynamic descriptors governing phase stability and lattice distortion. We further discuss established and emerging synthesis routes, from sol–gel and solid‐state reaction to field‐assisted and ultrafast methods, highlighting their advantages for compositional homogeneity and scalable production. Density functional theory, special quasirandom structures, and thermodynamic modeling are studied to provide computational understanding of stabilization and catalytic descriptors. Particular emphasis is placed on energy and environmental applications, including water splitting, oxygen reduction, CO_2_ reduction, ammonia decomposition, fuel cells, solar cells, and pollutant degradation, where HEPs demonstrate enhanced activity, durability, and resistance to scaling‐related limitations. Finally, key challenges in compositional complexity, mechanistic understanding, and sustainable synthesis are outlined, along with future opportunities in nonequimolar design, multianion engineering, and scalable fabrication for next‐generation entropy‐stabilized catalysts.

## Graphical abstract

This review examines high‐entropy perovskites (HEPs) as a promising catalyst platform for energy and environmental applications. The focus is on configurational entropy engineering, design and synthesis, and computational understanding. These materials possess tunable active sites, enhanced stability, and improved electronic interactions, leading to high performance in various catalytic reactions.

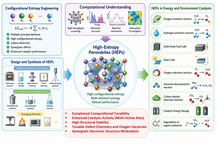

## 1 Introduction

The global shift toward sustainable energy and the urgent need to remediate environmental pollution have thrust catalytic materials into the spotlight of modern chemistry and engineering. Heterogeneous catalysts based on transition‐metal oxides must meet conflicting requirements: high intrinsic activity, selectivity, and durability under severe electrochemical conditions, while remaining cost‐effective and scalable. Conventional perovskite oxides are represented by the generic formula ABO_3_, where A is often a cation that belongs to the alkaline, rare‐earth, or earth groups and B is a transition metal. These oxides have maintained interest for a long time because of their appealing crystal chemistry, which allows them to contain about 90% of metallic elements without sacrificing structural integrity [1]. The B‐site transition metal is the major active site, determining redox activity, oxygen vacancy generation, and adsorption energies for critical intermediates in processes such as the oxygen reduction reaction (ORR), oxygen evolution reaction (OER), and CO_2_ reduction [2, 3]. Catalytic performance adjustment via A‐ and B‐site replacement techniques has therefore attracted much attention. There is still a dominant active‐site chemistry that limits the performance of typical perovskite oxides, even after decades of optimizing their composition. An essential trade‐off is imposed by the well‐known volcano‐type relationships, in which increasing the adsorption of one intermediate often weakens another. Moreover, long‐term operational stability remains a challenge, particularly given concerns about structural degradation and A‐site cation segregation under realistic operating conditions [4].

Metallurgy, not oxide chemistry, provided the conceptual breakthrough that allowed for a new realm of design possibilities. The concept of high‐entropy materials originated from high‐entropy alloys (HEAs) introduced by Yeh et al. and Cantor et al. in 2004 [5, 6]. HEAs are multicomponent metallic systems with five or more main components mixed, where each element typically occupies a concentration between 5 and 35 at%. This results in a configurational entropy (Δ*S*_conf _ ≥ 1.5R) sufficient to thermodynamically stabilize a single‐phase solid solution against phase segregation [7, 8]. The remarkable mechanical and functional features of these alloys have been ascribed to four distinct effects: sluggish diffusion, high‐entropy stabilization, severe lattice distortion, and the so‐called cocktail effect of synergistic multielement interactions. Metals were not the only ones to benefit from the idea of entropy‐driven stability. Subsequently, the high‐entropy strategy was extended to ceramic systems, leading to the development of high‐entropy oxides (HEOs). In 2015, Rost and coworkers reported the first single‐phase entropy‐stabilized oxide, (MgCoNiCuZn)O, which demonstrated that configurational entropy can stabilize multicomponent oxide lattices [9]. This discovery demonstrated that configurational entropy could drive a reversible multiphase‐to‐single‐phase transition in ionic crystals and that deliberate compositional disorder was a viable strategy for the discovery of entirely new classes of functional ceramics. This crucial finding set off a chain reaction of rapid expansion in HEOs, including rocksalt, spinel, fluorite, and, ultimately, perovskites [8].

**FIGURE 1:**
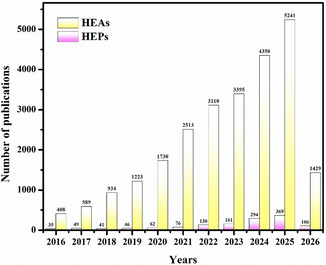
The number of publications on HEAs and HEPs from 2016 to 2026, as collected from the Web of Science database (updated on 22nd of April 2026).

In addition to using entropy engineering high‐entropy perovskites (HEPs) emerged as an advanced subclass of HEOs, where five or more cations are incorporated into either the A‐site, B‐site, or both sites of the ABO_3_ perovskite lattice. For example, the archetypal HEPOs system, which was reported by Jiang et al. [10], demonstrates a continuous distribution of adsorption energies across its surface, rather than a single, fixed value, when five or more transition‐metal cations occupy the B‐site simultaneously. This feature successfully avoids the scaling relations that restrict single‐active‐site catalysts by allowing the simultaneous accommodation of numerous reaction intermediates. Compared with traditional perovskite oxides, HEPOs exhibit several interesting properties. These include improved ferroelectric relaxation [11, 12, 13, 14], lower thermal conductivity [15, 16, 17], high proton conductivity [18], better catalytic activity [19, 20, 21], higher oxygen permeability [22], distinct luminescence sequence [23], unique microstructure in magnetic materials [24, 25], and greater thermal and chemical stability [26, 27]. In addition to its catalytic activity, high‐entropy's thermodynamic stabilization has been demonstrated to significantly reduce elemental segregation, maintain structural integrity at high temperatures, and sustain electrochemical performance over long periods of operation. These properties are essential for practical use in electrolyzers, photovoltaic devices, and solid oxide fuel cells (SOFCs) [28, 29]. The year‐wise publishing trends for HEAs and HEPs from 2016 to 2026 are displayed in Figure 1, which demonstrates a steady rise in HEPs and a rapid increase in HEAs.

Despite this momentum, the field remains at an early stage. There are still unresolved critical concerns regarding the relationships between compositional entropy, local atomic ordering, surface reconstruction, and catalytic mechanism. There is still a lack of systematic approaches to bridge the gap between device integration in practice and performance on a laboratory scale, and there is a lack of rational design techniques that expand beyond equimolar substitution to investigate the enormous nonequimolar compositional space. This review provides a comprehensive assessment of the current status and emerging applications of HEP materials as energy and environmental catalysts. We commence with the main design paths that researchers have at their disposal and go over the thermodynamic principles that determine configurational entropy in perovskite lattices. After a brief overview of synthesis methodologies, including both established and newer methods like rapid sintering and low‐temperature aqueous approaches, we move on to discuss the computational tools that begin to shed light on the mechanisms at work in these intricate systems. The primary focus of the review is on electrocatalytic and photocatalytic applications, which include water electrolysis (OER, HER, ORR), SOFCs, solar cells, CO_2_ reduction, ammonia decomposition, and organic pollutant degradation. The review includes a critical analysis of performance benchmarks and a deeper comprehension of the mechanisms involved. The review concludes with a perspective on sustainability, scalability, and the directions most likely to define the next decade of HEP research.

## 2 Configurational Entropy Engineering

Typically, systems that have a configurational entropy greater than 1.5R are often categorized as high‐entropy systems. This method is used to calculate the configurational entropy of perovskite‐type oxides [30]:

(1) $$\text{Δ}S_{\text{config}}=-R\left(\sum_{i=1}^{N}A_{i}\ln A_{i}+\sum_{j=1}^{M}B_{j}\ln B_{j}+3\sum_{q=1}^{P}O_{q}\ln O_{q}\right)$$

where *R* represents the gas constant, A*_i_*, B*_j_*, and O*_q_* refer to the mole fractions of the *i* ^th^, *j* ^th^, and *q* ^th^ ions, respectively, and *N*, *M*, and *P* denote the number of ions occupying the A, B, and O sites within the perovskite structure, respectively. Currently, very few HEPOs have been documented that depend on the oxygen lattice contribution to entropy in order to attain high configurational entropy. Accordingly, by eliminating the anionic lattice's effect from the compound's configurational entropy, Equation ((1)) can be reduced to $\text{Δ}S_{\text{config}}=\text{Δ}S_{A,\text{config}}+\text{Δ}S_{B,\text{config}}$. Thus, there are two principal approaches to constructing HEPOs: Route 1 involves the occupation of either the B‐site or A‐site by multiple cations, whereas Route 2 involves the occupation of both A‐ and B‐sites by multiple cations.

For Route 1, only a single sublattice will undergo substitution, resulting in the formula being expressed as $\text{Δ}S_{\text{config}}=-R\sum_{i=1}^{N}x_{i}\ln x_{i}$, where *x* *_i_* denotes the mole fraction of the *i* ^th^ ion at the specified site within the perovskite structure. If an equimolar substitution is performed, the formula may be further reduced to $\text{Δ}S_{\text{config}}=\ln(N)R$, where *N* represents the number of species on the fixed sublattice. This design technique is often used. Jiang and colleagues developed 13 Ba/Sr‐based HEPOs utilizing this method [10]. Using tetravalent ions (Ti, Sn, Zr, and Hf, along with one variable ion) in place of the B site creates six molecules that only have perovskite structures. Perovskite is characterized by the presence of two distinct cation sublattices. Sarkar et al. developed eleven rare‐earth‐transition metal HEPOs through modifications at various replacement sites [31]. For Route 2, $\text{Δ}S_{A,\text{config}}$ and $\text{Δ}S_{B,\text{config}}$ are not required to exceed 1.50R. As long as the combined total of $\text{Δ}S_{A,\text{config}}$ and $\text{Δ}S_{B,\text{config}}$ exceeds 1.50R, the system is classified as high‐entropy. For instance, the $\text{Δ}S_{A,\text{config}}$ values for Ba_0.5_Sr_0.5_(Zr_0.4_Hf_0.3_Ti_0.3_)O_3_ and Ba_0.4_Sr_0.4_Bi_0.2_(Zr_0.3_Hf_0.3_Ti_0.2_Fe_0.2_)O_3_ are 0.69R and 1.05R, respectively, while their $\text{Δ}S_{B,\text{config}}$ values are 1.09R and 1.37R. Consequently, the $\text{Δ}S_{S,\text{config}}$ values for both exceed 1.50R ultimately [32].

Table S1 provides an overview of the compositions, ion‐substitution sites, and preparation methods of HEPOs reported in the works. The “A/B site” denotes the position within the crystal structure of HEPOs where two or more ions can coexist, resulting in elevated configurational entropy. In Table S1, it is evident that specific ions, including Ca, La, Ba, and Bi, are commonly located at the A site, whereas Zr, Ti, Fe, Ni, and Co are predominantly situated at the B site. For instance, Bui et al. synthesized a rare‐earth‐based ultra‐HEP oxide containing 16 ions at the A site [33]. The configurational entropy contributes to the thermodynamic stabilization of single‐phase multicomponent perovskite structures, suppressing phase segregation and improving long‐term operational stability under harsh catalytic conditions. At the same time, lattice distortion induced by ionic radius mismatch alters local coordination environments and electronic structures, thereby regulating oxygen vacancy concentration, charge redistribution, and adsorption/desorption energetics of reaction intermediates. In addition, the cocktail effect arising from multielement interactions generates unique synergistic catalytic behaviors that cannot be achieved in conventional single‐component perovskites.

Table S1 indicates that 81% of the enumerated components are nonstoichiometric. The production of these nonstoichiometric components is possible by the replacement of ions within the elements that possess stoichiometric properties. For instance, in the stoichiometric compound Ba(Zr_0.2_Sn_0.2_Ti_0.2_Hf_0.2_Ce_0.2_)O_3_, Ce^4+^ is substituted with Y^3+^ to produce the nonstoichiometric compound Ba(Zr_0.2_Sn_0.2_Ti_0.2_Hf_0.2_Y_0.2_)O_3_ [18]. Likewise, Yb^3+^ in Ba(Zr_0.2_Ti_0.2_Sn_0.2_Yb_0.2_Nb_0.2_)O_3_ is substituted with Hf^4+^ to produce Ba(Zr_0.2_Ti_0.2_Sn_0.2_Hf_0.2_Nb_0.2_)O_3_ [34, 35]. This suggests that the issue of elemental limitations becomes more significant when developing stoichiometric HEPO components. Consequently, designing more stoichiometric HEPO components presents a significant challenge. Therefore, in order to address this obstacle, Tang et al. developed up with the valence‐band combination technique for the construction of HEPOs [35]. This approach could segmented into two components. Initially, the possible systems are identified by examining the valence states of various ions to guarantee charge neutrality. The systems are subsequently partitioned into multiple subsystems. Subsequently, the replacement ions are selected on the basis of their geometric compatibility within these subsystems. In addition to demonstrating that the valence state combination technique is feasible, they presented a novel approach to the development of HEPOs.

Nevertheless, Table S1 also highlights another concern. Numerous components detailed in Table S1 attain higher configurational entropy through the substitution of various ions in equal amounts to inhabit a crystal lattice. Nevertheless, an excessive emphasis on this equimolar approach may result in the oversight of the extensive range of nonequimolar high‐entropy constituents. To study this nonequimolar component space efficiently, Ma and colleagues suggested an approach that uses the valence‐state combination strategy with Ba‐based HEPOs [36]. They proposed 122, 66, 86, and 66 valence‐combination systems, each providing an average valence of +2, +3, +4, and +5, respectively. They also identified 37, 29, 56, and 29 valence‐combination systems involving high‐entropy compounds, each within a 1% interval of constituent composition. Their research advances the valence combination strategy, enabling a comprehensive investigation of the extensive nonequimolar element space of HEPOs through the application of the finite elements of the periodic table. In addition, this method makes it easier to generate a wide variety of nonequimolar and nonstoichiometric HEPOs by facilitating the replacement of ions.

For the selection of HEPO components, a transition from a component‐based approach to a function‐oriented methodology is anticipated over time. Using the valence state combination technique, the idea of the morphotropic phase boundary (MPB) in (1−*x*)Pb(Mg_0.2_Zn_0.2_Nb_0.2_Ta_0.2_W_0.2_)O_3−*x*_PbTiO_3_ [37]. The piezoelectric and ferroelectric characteristics of this system are examined. Subsequently, the implementation of a high‐entropy approach has resulted in exceptional piezoelectric characteristics (*d*_33_ = 1210 pC/N) in Pb(Ni_0.177_Sc_0.015_In_0.06_Ti_0.32_Nb_0.428_)O_3_ possessing a novel flexible polarization configuration [38]. HEPOs have the potential to improve their dielectric energy storage capacity by utilizing lattice distortion, compositional variations, and random fields. This may be accomplished by utilizing these properties. The great thermodynamic stability in HEPOs is thought to be due to the high‐entropy effect, which also accounts for their remarkable electrochemical and structural stability. The synergistic impact of various ionic contacts is thought to be responsible for their increased catalytic efficiency.

HEPOs comprise multiple ions distributed across two cation sublattices, excluding the consideration of the oxygen site. This stability of HEPOs is connected with the radii of these ions as well as their valence states. The stabilization state of Ba/Sr(5B)O_3_ was initially predicted by utilizing the ionic radius deviation (*δ*(*r*)) and the tolerance factor (*t*) [10]. In this system, the permissible range for *t* is 0.97–1.03, and a *t* value nearer to 1 signifies a more facile construction of the cubic structure. Nonetheless, the influence of *δ*(*r*) on phase stabilization remains uncertain. Furthermore, it proposes using *t* to predict HEPO configurations, suggesting that *t* is a necessary but insufficient criterion for the formation of a stable single‐phase perovskite structure. The adjusted range for *t* reflects the various stable configurations of HEPOs. Both the orthogonal and rhombic perovskite phases are stable in rare earth‐transition metal (RE‐TM) systems within the range of 0.878–0.953 [31] and 0.9537–0.9622 [26], respectively.

Unfortunately, it is not possible to ensure a stable structure based just on *t*. In RE‐TM systems, it is important to note that not all samples need to exhibit an orthogonal structure and have *t* values within the range of 0.878–0.953 [31]. Forecasting stable configurations in multicomponent HEPOs through the utilization of the *t* parameter presents considerable challenges. Recently, Yu et al. developed A_32_Ti_8_Sn_8_Nb_4_Ta_4_Me_8_O_96_ (A = Sr, Ba; Me = Ga, Fe) utilizing the cluster‐plus‐glue‐atom model, which accounts for short‐range chemical ordering [39]. This paradigm, derived from metallic glasses and quasicrystals, has been utilized in the study of inorganic compounds [40, 41]. This approach enhances the structural stability of HEPOs by reducing differences in crystal structure across single‐cationic oxides in raw materials. Employing this approach, Spiridigliozzi et al. developed an entropy‐stabilized perovskite, Ba(Ce_0.2_Zr_0.2_Gd_0.2_Y_0.2_La_0.2_)O_2.7_ [42]. Furthermore, the existence of an additional phase may also affect the development of a single HEPO. In (Bi_0.2_Ln_0.2_Sr_0.2_K_0.2_Na_0.2_)TiO_3_ (Ln = La, Pr, Ce, Eu, Nd, Tb, Tm, Sm, Ho, Gd, Dy, Er, Yb), the ionic radii of Ln^3+^ are correlated with the stability of the Ln_2_Ti_2_O_7_ pyrochlore‐like phase during the synthesis of HEPOs [43]. The pyrochlore‐like phase remains stable when 1.46 < *R*_A_^3+^/*R*_B_^4+^ < 1.78. The stability of the pyrochlore‐like structure decreases with higher Ln^3+^ radius, favoring the development of a single‐perovskite structure [43].

Due to the insufficient predictive accuracy of the structural descriptor *t* regarding HEPO stability, alternative parameters were subsequently introduced as primary or auxiliary indicators for structural prediction. When designing HEPOs, it is necessary to take into account both electrical balance and geometric compatibility. This may be achieved by the use of the valence combination technique [35, 36]. According to Tang et al., they proposed 12 Ba‐based HEPOs with equal amounts of B‐site replacement using the valence‐combination approach [35]. HEPO stability was initially assessed using the valence deviation *δ*(*V*). This was done on the basis of the discrepancy in ionic valence states. nevertheless, an analysis of the prediction outcomes for *t*, *δ*(*V*), and *δ*(*r*) across 20 HEPO systems (comprising 12 equimolar [35] and 8 nonequimolar [36] systems indicates that *t* is the predominant factor influencing the structural prediction of HEPOs. It should be noted that ordered structures appear in these systems when the *δ*(*V*) value is above 0.35. This hypothesis, substantiated by the presence of the corresponding ordered arrangements within HEPOs, holds particular significance for HEPOs that are nonequimolarly replaced by multiple nonequivalent ions.

(2) $$t=\frac{r_{A}+r_{O}}{\sqrt{2}\left(r_{B}+r_{O}\right)}$$

(3) $$\delta \left(r\right)=\sqrt{\sum_{i=1}^{N}C_{i}{\left[\frac{r_{i}-\bar{r}}{\bar{r}}\right]}^{2}}$$

(4) $$\delta \left(V\right)=\sqrt{\sum_{i=1}^{N}C_{i}{\left[\frac{V_{i}-\bar{V}}{\bar{V}}\right]}^{2}}$$

(5) $$V_{\sigma }=\frac{\sqrt{\sum_{i=1}^{N}{\left(x_{i}-\bar{x}\right)}^{2}}}{\bar{x}}$$

While no definitive correlation exists among the three parameters (*δ*(*V*), *δ*(*r*), and *t*) and the structural characteristics of HEPOs, numerous investigations have examined the factors affecting the design of HEPOs with varying compositions. For instance, the standard deviation parameter *V*_σ_ characterizes the lattice distortion in HEAs containing two metal sites [44]. The presence of numerous ions at a single‐metal site causes a little lattice deformation in the stable phase of a high‐entropy substance with two metal positions. Equations ((2))–((5)) denote the descriptors of *δ*(*V*), *δ*(*r*), *t*, and *V*_σ_, respectively. These descriptors demonstrate significant shortcomings when evaluated solely based on the geometric factor. The *δ*(*V*) and *δ*(*r*) are ineffectual in assessing HEPOs with both A and B sites occupied by ions. Conversely, utilization of *V*_σ_ is confined to the prediction of high‐entropy compounds possessing two metal sites. Among the listed descriptors, *t* has the most significant influence on structural prediction, as indicated by the structural data that is currently available for HEPOs. Future studies should focus on the continuous effort of examining structural descriptors in this context.

In addition to the aforementioned parameters, thermodynamic considerations are further employed to forecast the stable configurations of HEPOs. The impact of configurational entropy on the development of a stable phase was highlighted in (Mg_0.2_Co_0.2_Ni_0.2_Zn_0.2_Cu_0.2_)O [9]. Sharma and colleagues employed thermodynamic descriptors to assess the relative formation propensity of HEPOs [13]. Shao and his research group integrated density functional theory (DFT) with special quasirandom structure (SQS) to investigate the structural properties, mechanical characteristics, and thermodynamic behavior of Ba/Sr(Zr_0.2_Sn_0.2_Ti_0.2_Hf_0.2_Nb_0.2_)O_3_ [45]. The presence of numerous metal ions causes a negative enthalpy shift, which makes HEPOs structurally stable [45]. Zhong et al. employed an adapted integrated defect chemistry approach alongside the CALPHAD (Calculation of Phase Diagrams) method to investigate the combined behavior of various ions [46]. The Gibbs free energy of various oxides predominantly governs this behavior, with minor secondary effects arising from oxygen partial pressure, temperature, constituents, and domain structures [46].

Recent studies have demonstrated that nonequimolar HEPs can provide greater flexibility in tuning electronic structure and catalytic activity compared with conventional equimolar systems. For example, Ma et al. systematically explored nonequimolar Ba‐based HEPs using a valence‐state combination strategy and demonstrated that slight deviations from equimolar compositions can effectively regulate lattice distortion, local coordination environments, and phase stability [36]. Their work highlighted that nonequimolar compositional engineering enables access to a much broader compositional space while maintaining high configurational entropy, thereby offering additional opportunities for optimizing catalytic performance and structural stability. Similarly, Yang et al. reported that nonequimolar cation distributions can induce ordered local environments and modify the valence deviation parameter, leading to distinct electronic structures and defect chemistry compared with equimolar HEPs [47]. These findings suggest that precise compositional tuning beyond the equimolar approach may become an important strategy for tailoring adsorption energetics, oxygen vacancy concentration, and charge‐transfer kinetics in future HEP catalysts.

In addition to cation engineering, mixed‐anion or multianion high‐entropy systems have recently emerged as a promising research direction. Incorporation of multiple anions such as oxygen, nitrogen, sulfur, fluorine, or halides into high‐entropy lattices can significantly modify the electronic band structure, metal–anion covalency, and defect distribution. For instance, oxyfluoride and oxynitride HEP systems have been reported to exhibit enhanced electronic conductivity and improved catalytic activity due to altered orbital hybridization and increased oxygen vacancy mobility [48]. Multianion engineering is therefore expected to provide additional control over surface reactivity, charge redistribution, and intermediate adsorption behavior, making it a highly attractive strategy for next‐generation energy and environmental catalysts.

It is difficult to suggest a single useful descriptor that can precisely anticipate the stable phase structures of HEPOs since so many variables affect their structure. To address this challenge, the combined impact of thermodynamic and geometric factors of various ions on phase formation can be thoroughly examined.

## 3 Design and Synthesis of HEPs

Synthesis of impurity‐free HEPs often involves numerous processes, such as sol dispersion, mechanical ball milling, and high‐temperature reaction, because of the structural complexity of these compounds [49]. Also, our previous research has demonstrated that compared to traditional perovskites, which typically require synthesis temperatures of around 1000°C [50, 51, 52, 53, 54], HEPs often necessitate temperatures of 1200°C–1600°C [10, 55]. As a result, developing practical techniques for HEP synthesis has been a major research focus in recent decades, as described in detail below. Table S1 provides a compilation of HEPs synthesized using various preparation techniques.

### 3.1 Traditional Synthesis Techniques

#### 3.1.1 Solid‐State Reaction (SSR)

**FIGURE 2:**
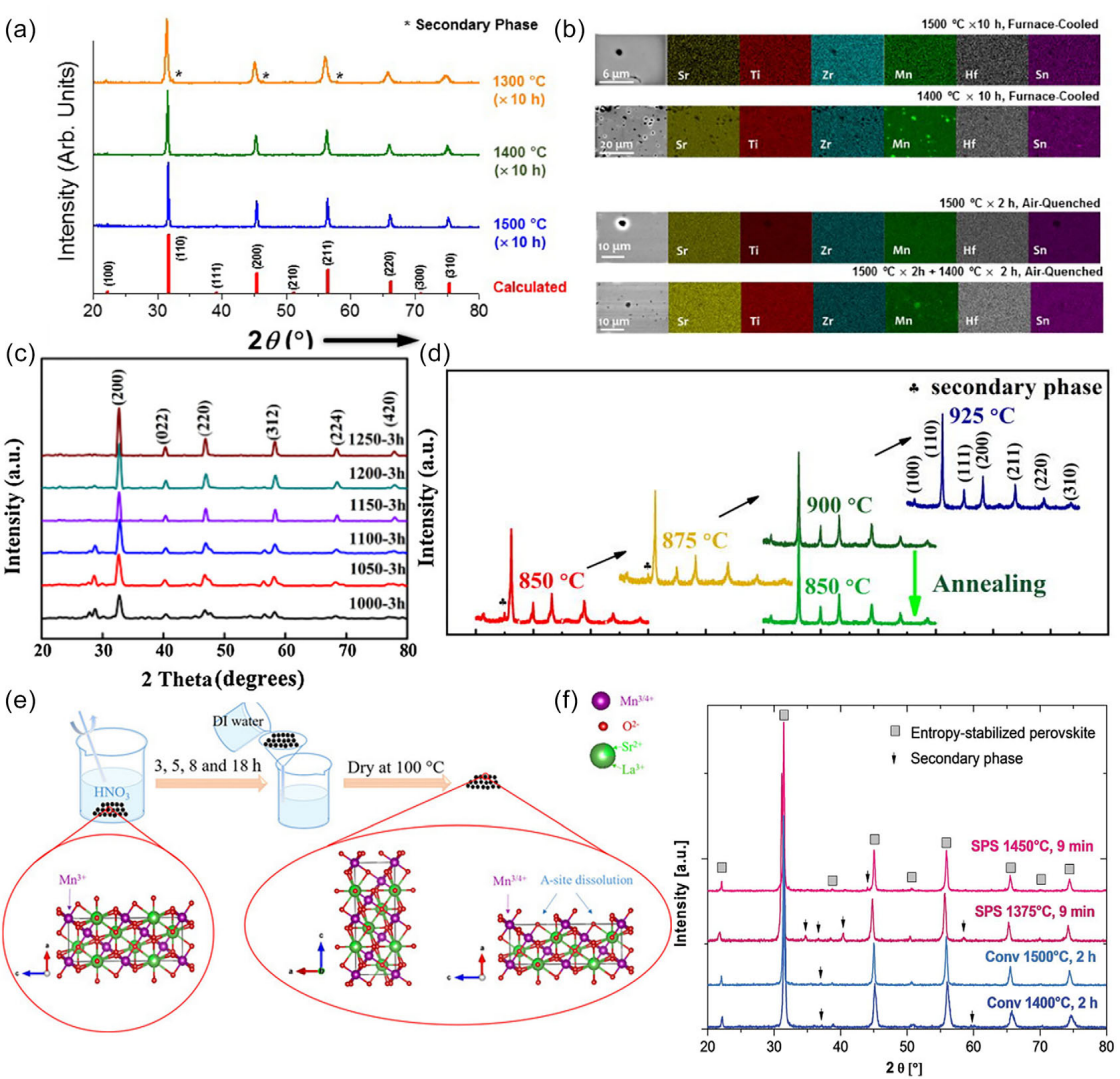
(a) XRD patterns of Sr(Zr_0.2_Sn_0.2_Ti_0.2_Hf_0.2_Mn_0.2_)O_3_ prepared by SSR and sintered at various temperatures. (b) Cross‐sectional EDXS elemental mapping of Sr(Zr_0.2_Sn_0.2_Ti_0.2_Hf_0.2_Mn_0.2_)O_3_ pellet. Reproduced with permission [10]. Copyright 2018, Elsevier. (c) XRD pattern of synthesized [(Bi, Na)_1/5_(La, Li)_1/5_(Ce, K)_1/5_Ca_1/5_Sr_1/5_]TiO_3_ HEP by the SSR technique. Reproduced with permission [57]. Copyright 2020, Springer. (d) XRD patterns of (Na_0.2_Bi_0.2_Ba_0.2_Sr_0.2_Ca_0.2_)TiO_3_ powders synthesized by SSR method and calcined at various temperatures. Reproduced with permission [58]. Copyright 2019, AIP Publishing. (e) Schematic drawing of the preparation of the HNO_3_ treated La_0.8_Sr_0.2_MnO_3−*δ*_ (MnOx/LSMO) structure by a modified sol–gel method. Reproduced with permission [59]. Copyright 2018, John Wiley and Sons. (f) XRD patterns of the Sr((Zr_0.94_Y_0.06_)_0.2_Sn_0.2_Ti_0.2_Hf_0.2_Mn_0.2_)O_3−*x*_ HEP prepared by SPS at different temperatures. Reproduced with permission [55]. Copyright 2019, Taylor & Francis.

A conventional approach to ceramic synthesis is the SSR [56]. Precursor powders (oxides or carbonates) are mechanically ball milled and calcined (often over 1000°C) in conventional SSR. Then, to achieve compositional homogeneity, the mixture is remixed and recalcined. Sintering is typically carried out for a duration of 10–24 h to provide sufficient cationic dispersion among the precursors. Jiang et al. were the first to disclose the critical role of temperature in HEP synthesis through the synthesis of Sr(Zr_0.2_Sn_0.2_Ti_0.2_Hf_0.2_Mn_0.2_)O_3_ at temperatures from 1300°C to 1500°C [10]. Figure 2a,b shows that element mapping using X‐ray diffraction (XRD) and energy‐dispersive X‐ray spectroscopy (EDS) revealed the existence of an additional phase below 1400°C. Nevertheless, entropy‐driven effects were responsible for the inhibition of secondary phase development during sintering at 1500°C [10].

According to the research, the sintering temperature of SSR varies depending on whether it is an A‐site or a B‐site replacement [57]. The typical sintering temperature range for B‐site HEPs is 1300°C–1600°C [10, 34]. On the other hand, the sintering temperature for A‐site HEPs is between 900°C and 1250°C [57, 58]. This temperature decrease could be explained by two factors: (1) the carbonate precursors from which A‐site elements are derived mainly decompose at approximately 900°C (e.g., 825°C for CaCO_3_ [60] and 891°C for 2CO_3_ [61] and (2) the fact that A‐site elements may readily diffuse inside perovskites lattices and are frequently volatile [62]. Unlike most B‐site HEPs, pure [(Bi, Na)_1/5_(La, Li)_1/5_(Ce, K)_1/5_Ca_1/5_Sr_1/5_]TiO_3_ was synthesized at a significantly lower temperature of 1200°C by Yan et al. Figure 2c [57]. Figure 2d shows that (Na_0.2_Bi_0.2_Ba_0.2_Sr_0.2_Ca_0.2_)TiO_3_ was successfully synthesized at 900°C by Pu et al. [58]. The same researchers also synthesized many four‐element non‐HEPs at 900°C, including (Bi_0.25_Ba_0.25_Sr_0.25_Ca_0.25_)TiO_3_, (Na_0.25_Ba_0.25_Sr_0.25_Ca_0.25_)TiO_3_, (Na_0.25_Bi_0.25_Sr_0.25_Ca_0.25_)TiO_3_, and (Na_0.25_Bi_0.25_Ba_0.25_Sr_0.25_)TiO_3_. All of these materials were found to contain impurities, as indicated by four chemical elements. The lower the Δ*G* of HEPs with a larger Δ*S* compared with typical perovskites, the higher the possibility of a single‐phase formation and enhanced stability, since the formula for a material's Gibbs free energy is Δ*G *= Δ*H* − TΔS.

#### 3.1.2 Sol‐Gel Method

The sol–gel approach facilitates a more uniform blending of precursor cations than SSR. In comparison to SSR, this enables a reduced calcination temperature and the formation of smaller particles [59]. Aqueous dissolution of cationic nitrates is the initial step in the standard sol–gel synthesis process. In order to generate cationic chelates, citric acid and ethylenediaminetetraacetic acid (EDTA) are added. At the same time, a solution of ammonia in water (NH_3_·H_2_O) is added to bring the pH to around 7. Figure 2e shows the subsequent steps in producing ceramic powder by calcining the dried gel at temperatures ranging from 700°C to 900°C [59, 63]. As an illustration, the sol–gel process was used to successfully synthesize La_1−*x*_Sr_*x*_(Co, Cr,Fe, Mn,Ni)O_3−δ_ (*x* = 0, 0.1, 0.2, 0.3, 0.4, 0.5) with an average size of the crystal ranging between 18.2 and 27.4 nm at 700°C [26]. Another group, Yang et al., also documented the development of HE‐LSM (La_0.2_Pr_0.2_Nd_0.2_Sm_0.2_Sr_0.2_MnO_3−δ_), using the sol–gel technique at 1000°C [64]. Nevertheless, low‐temperature synthesis is not possible for all HEPs. For instance, Zhao and colleagues reported that the sol–gel technique necessitates calcination at 1500°C for the synthesis of (Y_0.2_Nd_0.2_Sm_0.2_Eu_0.2_Er_0.2_)AlO_3_ due to the sluggish diffusion of rare‐earth aluminates and low thermal conductivity of the material [65]. The sol–gel technique is not much better than SSR for materials in this category.

#### 3.1.3 Spark Plasma Sintering (SPS)

An approach to pressure‐assisted sintering that uses pulsed direct current and operates at low voltage is known as SPS [66]. A standard SPS furnace consists of an inert gas around a pressurized graphite die. When powered by a high current (1–10  kA), the graphite die‐punch performs as a heating substance. Because of graphite's great electrical conductivity, substantial currents may be generated at voltages lower than 10 V. This makes Joule heating very efficient, with a heating range of up to 1000°C min^−1^. Biesuz et al. synthesized Sr((Zr_0.94_Y_0.06_)_0.2_Sn_0.2_Ti_0.2_Hf_0.2_Mn_0.2_)O_3−δ_ using SPS (Figure 2f) [55]. The authors initially combined strontium oxide and transition‐metal oxide precursor particles with a binder (QPAC 40) via ball milling. Subsequently, the mixture was dry‐pressed into pellets. In about 9 min, the aforementioned HEP was formed using a single‐SPS step at 1475°C. When compared, SSR required 2 h.

#### 3.1.4 Flash Sintering

Another field‐assisted technology that is comparable to SPS is flash sintering [67]. In contrast to SPS, flash sintering has two key differences: (1) Before flash sintering can begin, the material is typically preheated to temperatures between 800°C and 1000°C. (2) Instead of using an external heating source, the sample is heated by the Joule effect as a result of an applied current [68]. Thus, samples must have enough electrical conductivity for flash sintering. The bulk Joule heating makes it possible to obtain sample temperatures exceeding 1000°C in a matter of minutes or even seconds, much like an incandescent light bulb. Wang et al. [69] and Liu et al. [70] reported that the synthesis time of Sr(Ti_0.2_Y_0.2_Zr_0.2_Sn_0.2_Hf_0.2_)O_3−*x*_ was reduced to only 1 min by flash sintering at an electric field of 350 V cm^−1^ and a current density of 40 mA mm^−2^. There is an inherent limitation to both SPS and flash sintering that hinders performance in numerous applications: ceramic particles aggregate and grow to micron‐level diameters in the same sequence as those produced by dry pressing and sintering at temperatures exceeding 1000°C [55, 70].

#### 3.1.5 Nebulized Spray Pyrolysis (NSP)

The aerosol‐assisted nanoparticle preparation method known as NSP has the benefits of homogenous components and nanoscale fine powder [71]. Sarkar et al. synthesized A‐site, B‐site, and A‐B‐site HEPs using the NSP method [31]. The synthetic compounds had an average particle size of 180 nm and were named A(Co_0.2_Cr_0.2_Fe_0.2_Mn_0.2_Ni_0.2_)O_3_, (Gd_0.2_La_0.2_Nd_0.2_Sm_0.2_Y_0.2_)BO_3_, (B = Fe, Cr, Mn, Co, or Ni), and (Gd_0.2_La_0.2_Nd_0.2_Sm_0.2_Y_0.2_)(Co_0.2_Cr_0.2_Fe_0.2_Mn_0.2_Ni_0.2_)O_3_. Initially, the stoichiometric ratios of cationic nitrate precursors for A‐ and B‐site elements (e.g., La(NO_3_)_3_, Co(NO_3_)_2_·6H_2_O, etc.) were dissolved and mixed in water. After that, the hot‐wall reactor was filled with the combined aqueous precursor solution, and oxygen was blasted on top at a temperature of 1050°C. The presintered powder that was recovered was further calcined at 1200°C for 2 h to produce nanosized particles. Furthermore, Witte et al. successfully synthesized M(Co_0.2_Cr_0.2_Fe_0.2_Mn_0.2_Ni_0.2_)O_3_ (M = Gd, La, or Nb) and (Gd_0.2_La_0.2_Nd_0.2_Sm_0.2_Y_0.2_)(Co_0.2_Cr_0.2_Fe_0.2_Mn_0.2_Ni_0.2_)O_3_ using the NSP technique [72], further proving the significance and potential of this approach. The NSP approach is difficult to scale up because of the expenses of the hot‐wall reactor and atomizer [73]. Table S1 provides references to literature about the synthesize of HEPs using SPS, NSP, and flash sintering processes.

### 3.2 Low‐Temperature Synthesis Techniques

Conventional technologies such as SSR and sol–gel consume a lot of energy due to the high temperatures required. Consequently, there is a growing research initiative to reduce the synthesis temperature.

#### 3.2.1 Hydrothermal Synthesis

**FIGURE 3:**
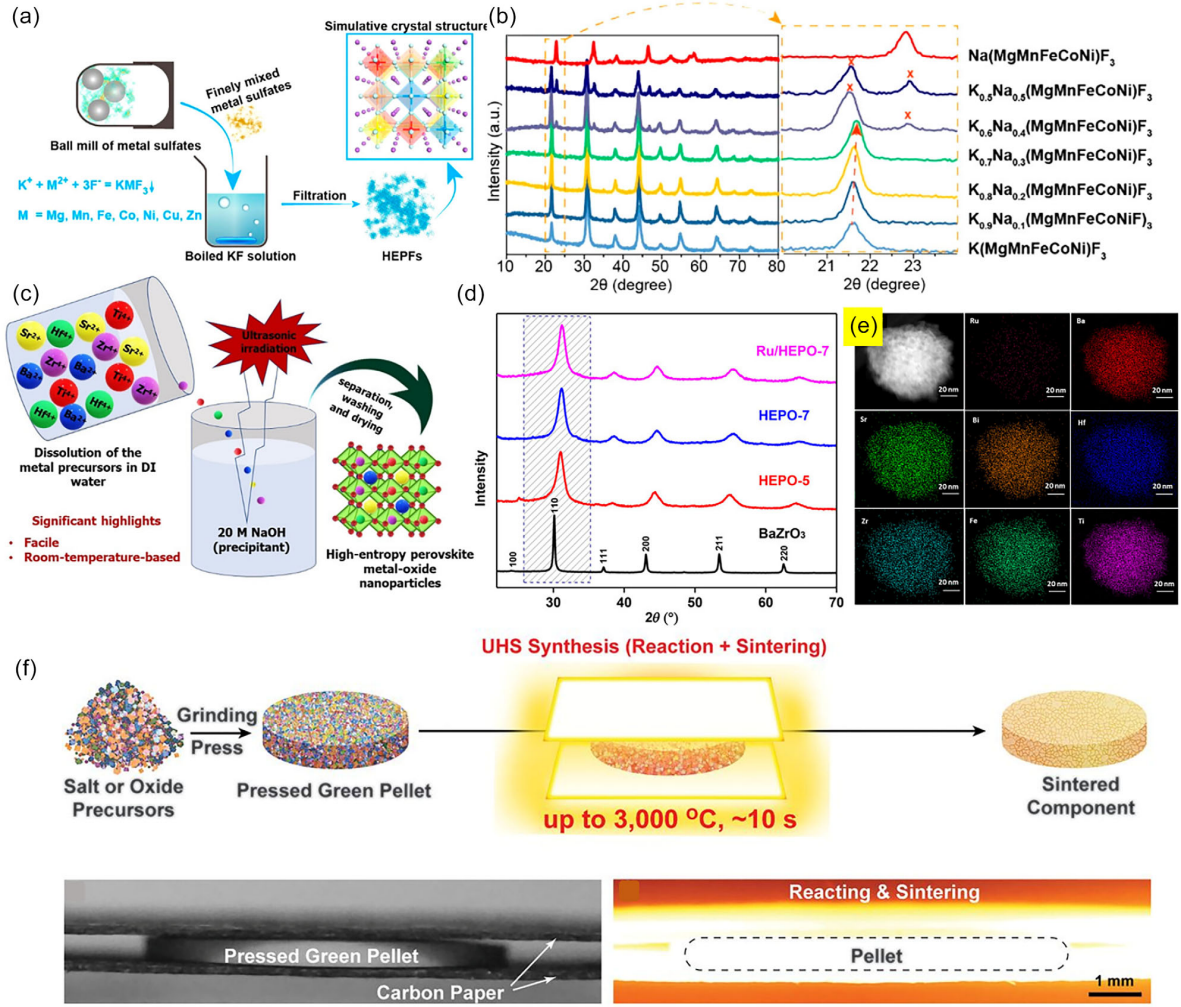
(a) Schematic representation of the preparation of K_1−*x*_Na_*x*_(MgMnFeCoNi)F_3_ (*x* = 0–3) HEP fluorides using a combination of hydrothermal and ball milling synthesis. (b) XRD patterns of K_1−*x*_Na_*x*_(MgMnFeCoNi)F_3_ HEP fluorides. Reproduced with permission [76]. Copyright 2020, American Chemical Society. (c) Schematic representation of the sonochemical preparation of HEP nanoparticles, (d) XRD patterns of BaZrO3, Ba_0.5_Sr_0.5_(Zr_0.4_Hf_0.3_Ti_0.3_)O_3_, Ba_0.4_Sr_0.4_Bi_0.2_(Zr_0.3_Hf_0.3_Ti_0.2_Fe_0.2_)O_3_, and Ru_0.13_/Ba_0.3_Sr_0.3_Bi_0.4_(Zr_0.2_Hf_0.2_Ti_0.2_Fe_0.27_)O_3_, (e) EDS elemental mapping of Ru/BaSrBi(ZrHfTiFe)O_3_ synthesized by an ultrasonication‐based technique without calcination. Reproduced with permission [32]. Copyright 2019, John Wiley and Sons, and (f) Schematic representation of the UHS synthesis process. Reproduced with permission [77]. Copyright 2020, Science.

A standard method to synthesize inorganic compounds is hydrothermal synthesis, which involves heating a precursor solution in a hydrothermal reactor [74]. Krawczyk et al. synthesized the five‐cation A‐site HEPO (Gd_0.2_Nd_0.2_La_0.2_Sm_0.2_Y_0.2_)CoO_3_ via a modified coprecipitation‐hydrothermal technique using NaOH or NH_3_ as a precipitant, followed by hydrothermal reaction at 150°C for 72 h, yielding an orthorhombic distorted single‐phase perovskite confirmed by HAADF–STEM and EDX mapping [75]. Similarly, Wang et al. employed hydrothermal reaction and ball milling to synthesize K(Mg_0.2_Mn_0.2_Fe_0.2_Co_0.2_Ni_0.2_)F_3_ at temperatures ranging from 100°C to 260°C [76]. To begin, precursor powders of MgSO_4_·H_2_O, FeSO_4_, Co(OAc)_2_·4H_2_O, Mn(OAc)_2_, and Ni(OAc)_2_·4H_2_O were ball‐milled for 10 min in stoichiometric quantities. Subsequently, the fine powder was added to boiling potassium fluoride and subjected to continuous heating for 10 min, resulting in the precipitation of K(Mg_0.2_Mn_0.2_Fe_0.2_Co_0.2_Ni_0.2_)F_3_ (Figure 3a,b) [76]. After this stage, the extraneous potassium fluoride and sulfate/acetate ions were eliminated through vacuum drying and filtration, resulting in the acquisition of purified K(Mg_0.2_Mn_0.2_Fe_0.2_Co_0.2_Ni_0.2_)F_3_ material. Another use of this low‐temperature approach was the preparation of pristine Na‐based and Na–K codoped HEP fluorides, which involved substituting NaF or KF–NaF mixed solutions for KF. In a recent study, Wang et al. synthesized HEP oxide‐halogenated solid solutions of [La(Cr_0.2_Mn_0.2_Fe_0.2_Co_0.2_Ni_0.2_)O_3_]_3/4_[K(Mg_0.2_Mn_0.2_Fe_0.2_Co_0.2_Ni_0.2_)‐F_3_]_1/4_ and (Ba_0.5_Sr_0.5_Co_0.8_Fe_0.2_O_3−δ_)_3/4_[K(Mg_0.2_Mn_0.2_Fe_0.2_Co_0.2_‐Ni_0.2_)F_3/2_ [78]. A sol–gel approach was used to generate the perovskites oxides (La(Cr_0.2_Mn_0.2_Fe_0.2_Co_0.2_Ni_0.2_)O_3_ and Ba_0.5_Sr_0.5_Co_0.8_Fe_0.2_O_3−δ_), while a hydrothermal technique was used to prepare the perovskite fluoride (K(Mg_0.2_Mn_0.2_Fe_0.2_Co_0.2_Ni_0.2_)F_3_). Moreover, a solid solution was obtained by mixing the perovskite oxides and fluorides using ball milling. The following are still inherent problems with hydrothermal synthesis that must be considered: (1) The high temperature and high‐pressure conditions necessitate hydrothermal reactors that are specially designed and costly. (2) It is not feasible to conduct practical applications, and the yield is poor.

#### 3.2.2 Sonochemical Synthesis

A high‐intensity ultrasonic source triggers cavitation, which in turn initiates chemical processes; this process is known as sonochemical synthesis [32]. Ultrasonic transducers are used to produce small bubbles, which are then used in the process of sonochemical synthesis. These bubbles are then used to form, grow, and finally implode. The implosion produces pressures greater than 2000 atm and temperatures greater than 5000°C over extremely short timeframes (≤1 ns) [79], whereas the synthesis system is typically kept at ambient temperature. Okejiri et al. used a sonochemical method to synthesize Ba_0.5_Sr_0.5_(Zr_0.4_Hf_0.3_Ti_0.3_)O_3_, Ba_0.4_Sr_0.4_Bi_0.2_(Zr_0.3_Hf_0.3_Ti_0.2_Fe_0.2_)O_3_, and Ru_0.13_/Ba_0.3_Sr_0.3_Bi_0.4_(Zr_0.2_Hf_0.2_Ti_0.2_Fe_0.27_)O_3_ nanoparticles [32]. A solution was prepared by mixing and dissolving stoichiometric cationic chlorides in deionized water. After that, the prepared solution was subjected to sonication in a 20 m NaOH solution at 20 kHz for approximately 10 min (Figure 3c–e) [32]. The final nanoparticles were obtained by centrifuging, washing, and drying the precipitate. The formation of tiny crystallites and particles with diameters of 5.9 and (76 ±  1.7) nm, respectively, was achieved in the HEPs when cavitation prevented the aggregation of ceramic particles [80]. Although ultrasound is highly appealing, it is important to acknowledge that it yields lower results than hydrothermal and solution reaction methods [81].

#### 3.2.3 Solution Reaction

Halide perovskites are commonly synthesized via a solution reaction, which can produce vast amounts of high‐quality components at room temperature [82]. Using a solution process, Yi et al. developed the high‐performance entropy‐stabilized perovskite solar cell (PSC) material Cs_0.2_FA_0.8_PbI_2.84_Br_0.16_ (where FA represents formamidinium, CH_5_N_2_^+^) [83]. Initially, precursors were synthesized by dissolving stoichiometric amounts of FAI, CsI, PbI_2_, and FABr in a combination of dimethylformamide and dimethyl sulfoxide at ambient temperature. Subsequently, mesoporous TiO_2_ films were spin‐coated with the solution and then treated with chlorobenzene. Following that, the coated films were annealed for 30 min at 100°C to produce Cs_0.2_FA_0.8_PbI_2.84_Br_0.16_. The material exhibited an impressive power conversion efficiency of around 17%, little hysteresis, and outstanding durability (1000 h) in ambient air, according to subsequent performance and stability tests, proving the dependability of the solution reaction process. It should not be disregarded, nevertheless, as the solution reaction approach has its own set of limitations, such as batch processing and low yields.

### 3.3 Emerging Techniques

As illustrated in the preceding sections, the development of HEP is impeded by low yields and high energy costs in numerous synthetic processes. Fast material preparation techniques are very desirable to lower production and energy costs, and associated CO_2_ emissions in ceramic synthesis.

In this regard, Wang et al. recently described ultrafast high‐temperature sintering (UHS), a quick material preparation technique (Figure 3f) [77]. Precursor materials are placed between two conductive carbon strips during the UHS process. This enables quick synthesis and sintering by quickly heating the carbon strips with direct current, creating a high‐temperature surroundings (1000°C–3000°C) with high heating (about 103°C–104°C min^−1^) and cooling (104°C min^−1^) rates. The sintering time of UHS is measured in seconds, not minutes, and it does not need electrical conductivity in the material, unlike SPS and flash sintering. An ultrafast sintering method that uses liquid nitrogen for cooling was established by Curcio et al. [84]. Phase metastable materials (such as some brownmillerite oxides) can be synthesized using qUHS technology, even if they are not amenable to ambient‐cooled traditional UHS. Although UHS is a promising new technique, it has a few problems that require fixing. One of them is the deterioration that occurs when carbon felt reacts with ceramics [85]. Another is the temperature inhomogeneity that occurs in thick or large samples [86]. It is possible to apply some of the methods used to synthesize conventional perovskites and oxides to the synthesis of HEPs. These include the methods discussed in this section, as well as microwave synthesis [87], self‐propagation high‐temperature synthesis [88], the microemulsion method [89], and the supercritical drying method [90]. Future trends in HEP synthesis and development will focus on combining economic‐environmental high‐quality synthesis methodologies.

Recently, high‐pressure torsion (HPT) has emerged as a promising nonequilibrium synthesis route for HEOs and perovskites [91]. HPT employs simultaneous application of severe plastic deformation and high pressure to induce atomic‐level mixing, defect generation, and metastable phase formation at relatively low temperatures. Compared with conventional thermal synthesis, HPT can accelerate solid‐solution formation while introducing high concentrations of dislocations, oxygen vacancies, and grain boundaries that are highly beneficial for catalytic and photocatalytic performance. Recent studies have demonstrated that HPT‐derived HEOs exhibit enhanced visible‐light absorption, improved charge separation, and superior photocatalytic activity owing to defect‐rich nanostructures and entropy‐stabilized electronic disorder. Moreover, HPT offers potential advantages for synthesizing metastable or compositionally complex HEPs that are difficult to obtain through equilibrium processing routes. Nevertheless, limitations, including restricted sample geometry, scalability challenges, and specialized high‐pressure equipment, still need to be addressed before industrial implementation.

Although numerous synthesis techniques have been successfully applied for HEPs, each method exhibits distinct advantages and limitations depending on the target composition, crystallinity, morphology, scalability, and intended application. Conventional SSR remains one of the most scalable and industrially viable approaches because of its simplicity, low precursor cost, and compatibility with bulk ceramic production. However, SSR generally requires prolonged calcination at high temperatures (typically >1200°C), which often results in large particle sizes, elemental segregation, and high energy consumption [9]. In contrast, sol–gel synthesis enables molecular‐level mixing of cations, lower synthesis temperatures, and improved compositional homogeneity, making it particularly suitable for oxide‐based HEP electrocatalysts and nanostructured materials [92]. Nevertheless, challenges related to organic additives, long processing times, and limited large‐scale production remain. Field‐assisted techniques such as (SPS, flash sintering, and UHS significantly reduce synthesis time and energy consumption while enabling rapid densification and entropy stabilization [66, 69]. SPS is especially advantageous for dense ceramic HEPs because of its ultrafast heating rates and minimized grain growth, whereas flash sintering offers extremely rapid synthesis within seconds or minutes. However, both methods are limited by equipment cost, sample‐size constraints, and temperature inhomogeneity, which may hinder industrial scalability. NSP provides excellent compositional uniformity and nanoscale particle formation, making it attractive for catalytic applications requiring high surface area, although its dependence on specialized reactors and atomizers restricts cost‐effective scale‐up [73]. Low‐temperature methods such as hydrothermal, sonochemical, and solution‐based synthesis are particularly attractive for halide‐based and nanostructured HEPs because they enable crystallization under mild conditions while suppressing phase decomposition and volatilization of halide species. Hydrothermal and sonochemical approaches can produce highly dispersed nanoparticles with controlled morphology and abundant defects, which are beneficial for photocatalytic and electrocatalytic applications [32, 76]. However, their relatively low yield, batch‐processing nature, and difficulty in continuous production limit practical implementation. Solution reaction methods are especially important for high‐entropy halide perovskites used in photovoltaic and optoelectronic applications because they allow low‐temperature film deposition and facile compositional tuning [83]. Despite these advantages, solvent sensitivity, moisture instability, and reproducibility remain major concerns for large‐scale device fabrication.

## 4 Computational Understanding of HEPs System

Research on atomistic models for high‐entropy materials is in the early stages [8, 93]. The computational endeavors are briefly reviewed in this section.

The Gibbs free energy, Δ*G*, can be used to establish a correlation between entropy effects and stabilization. This energy can be calculated as

(6) ΔG=ΔH–TΔS

Δ*H* represents the reaction enthalpy, whereas Δ*S* stands for the entropy and *T* is the temperature. Δ*G* = Δ*H* − *T*Δ*S*_config_ is obtained by assuming that Δ*S *= Δ*S*_config_. In general, the Δ*S* configuration is positive for materials with high‐entropy states. Therefore, the reaction free energy is reduced by a higher configurational entropy, which in turn facilitates the construction of materials at elevated temperatures [94]. In other words, the introduction of an increased number of elements results in an increase in *T*Δ*S*_config_, which in turn reduces Δ*G* and stabilizes the material.

**FIGURE 4:**
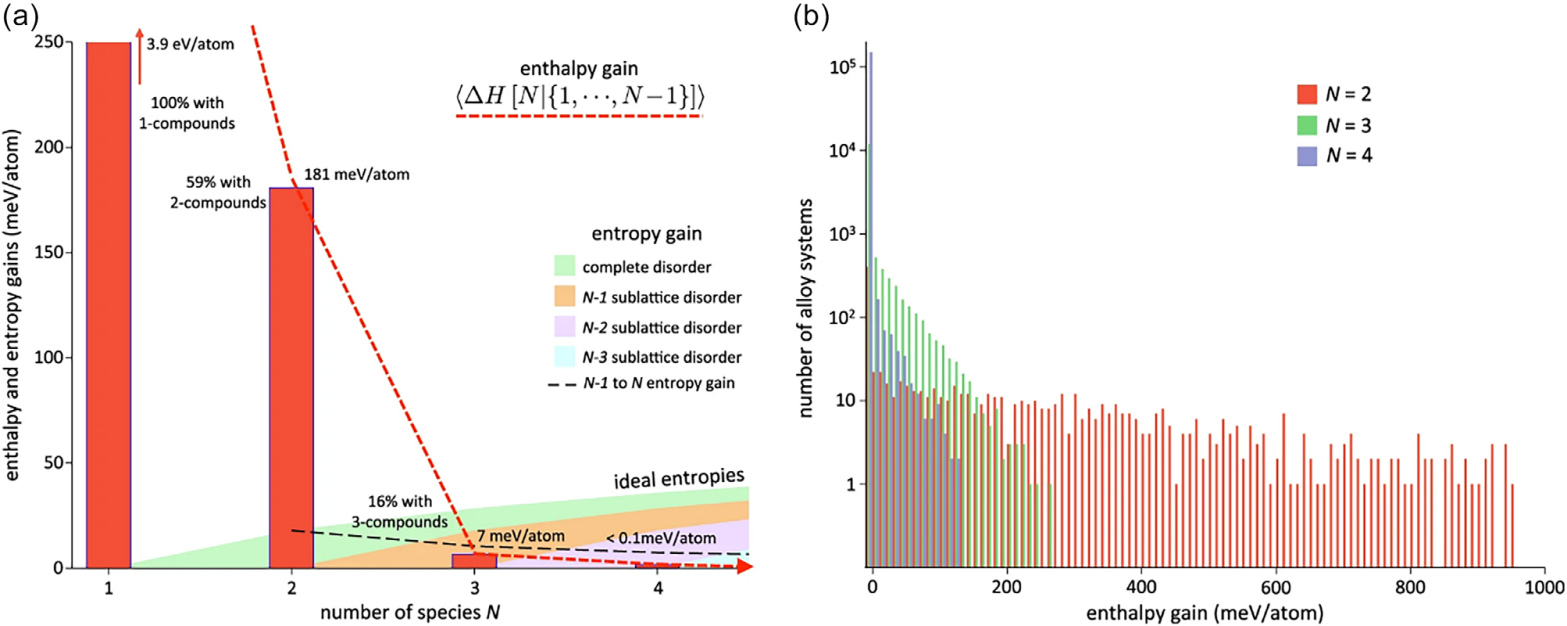
The rivalry between entropy and enthalpy increases as a function of the number of species, *N*. (a) Expected enthalpy gain (Δ*H*) and ideal entropy contributions as functions of the number of species, *N*. (b) Distribution of enthalpy gain for *N* = 2, 3, and 4. Reproduced with permission [93]. Copyright 2019, Springer Nature.

Moreover, it is important to consider that the introduction of additional elements may necessitate a higher enthalpy (Δ*H*) [93]. The energies of every chemical substance in the AFLOW.org materials repository were analyzed by Curtarolo and colleagues in order to comprehend the competition between Δ*H* and Δ*S*_config_ [95]. Δ*H* denotes the enthalpy of an N‐species substance in relation to all materials of ordered {1, …, *N* − 1}‐species [93]. Subsequently, the stable structure of the N‐ordered compound is indicated by Δ*H *≥ 0. Figure 4a illustrates the average Δ*H* for *N* = 1, 2, 3, and 4, in which Δ*H* surpasses the Δ*G* as defined in (4) for compounds with lesser *N*. The reduction in stabilization is suggested by the fact that Δ*H* decreases as the number of species, *N*, increases. The fact that the alloy frequency distribution drops with increasing *N* is further demonstrated in Figure 4b. Figure 4a also illustrates the ideal entropic contribution for the completely (*N*) and partially (*N* − 1, *N* − 2, *N* − 3) disordered systems. Furthermore, using log (*N*) − log (*N* − 1), Curtarolo and colleagues evaluated the entropy gain that occurs when a molecule of order *N* is phase‐separated into many disordered *N* − 1 components. Gains in entropy are greater than gains in enthalpy for *N* = 3 and *N* = 4, respectively, as illustrated in the black dashed line in Figure 4a. This suggests that the entropy effect stabilizes multicomponent and disordered systems.

**FIGURE 5:**
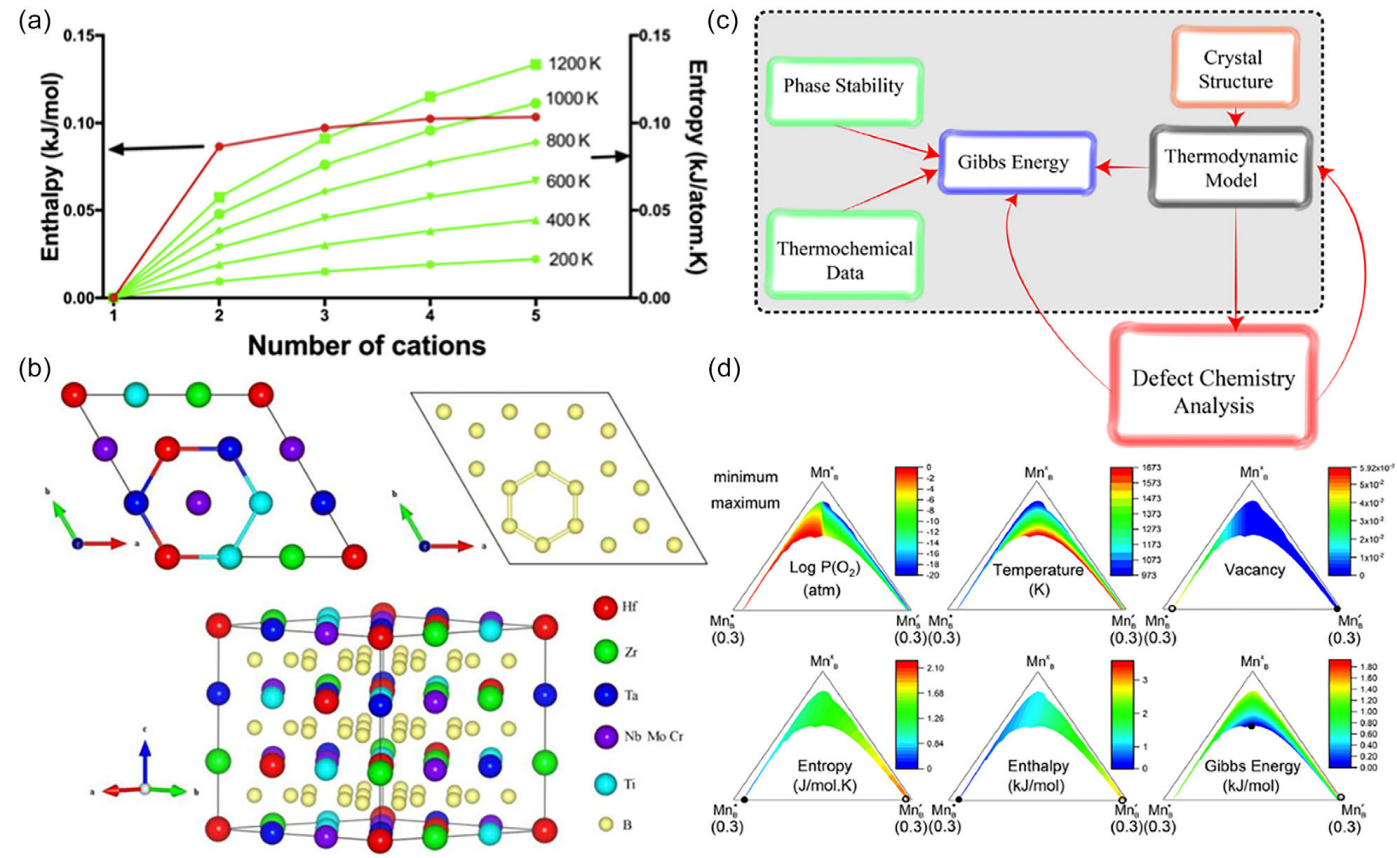
(a) Enthalpy and configurational entropy change as the number of cations increases. Reproduced with permission [105]. Copyright 2018, Elsevier. (b) Crystal structure of (Hf_0.2_Zr_0.2_Ta_0.2_M_0.2_Ti_0.2_)B_2_ (M = Mo, Nb, Cr). Reproduced with permission [97]. Copyright 2018, John Wiley and Sons. (c) Schematic illustration of the Integrated Defect Chemistry and CALPHAD method for HECs. (d) Projections of 2D mixing to display PO_2_, *T*, $V_{B}^{\prime \prime \prime }$, *G*_mix_, Δ*H*_mix_, and Δ*S*_mix_. Reproduced with permission [46]. Copyright 2019, Elsevier.

The formation mechanism of high‐entropy materials can be revealed through DFT simulations. Nevertheless, because DFT computations are expensive and scale cubically with atomic number; very few studies have used this approach to examine the characteristics of high‐entropy materials [96, 97, 98, 99]. In order to account for cationic disorder, the SQS algorithm [99], developed to simulate random (disordered) solid solutions, has been employed in combination with DFT. For instance, Rak et al. examined the (MgCoNiZn)_1−*x*_Cu_*x*_O (*x* = 0.13, 0.2, 0.26) system in terms of charge compensation, Jahn–Teller distortion, and electrostatic transferability [96, 100]. In the same vein, Zheng et al. investigated the adsorption energy of Li_2_S_6_ on the (100) plane of (Mg_0.2_Co_0.2_Ni_0.2_Cu_0.2_Zn_0.2_)O [101]. These authors found that the metal oxide has a negative binding energy of −6.92 eV, which is significantly lower than the reported value for carbon (−0.74 eV). This indicates that the metal oxide is more conducive to adsorption [101]. The polar–polar connections between Li polysulfide and the high‐entropy metal oxide were the primary cause of the strong interaction between Li_2_S_6_ and (Mg_0.2_Co_0.2_Ni_0.2_Cu_0.2_Zn_0.2_)O [102]. Utilizing DFT in tandem with an unconventional quasirandom structure, Katiyar et al. investigated the process of formic acid oxidation on CuAgAuPdPt HEA [103]. A synergistic interaction was observed to be facilitated by the metallic elements in the HEA, which resulted in a reduction of the adsorption energies of species and a reduction of all d‐band centers [103]. Kaufmann et al. demonstrated that combining high‐throughput DFT with statistical learning enables rapid prediction of phase stability and catalytic descriptors in compositionally complex oxides [104]. Such approaches are expected to accelerate the rational design of nonequimolar HEPs by identifying optimal combinations of cation disorder, oxygen vacancy concentration, and electronic structure descriptors. Wang et al. investigated the borides (Hf_0.2_Zr_0.2_Ta_0.2_M_0.2_Ti_0.2_)B_2_ (M = Nb, Mo, Cr) in a smaller supercell with 81 atoms (Figure 5b) [97], while Ye et al. investigated the carbides (Hf_0.2_Zr_0.2_Ta_0.2_Nb_0.2_Ti_0.2_)C and (Zr_0.25_Nb_0.25_Ti_0.25_V_0.25_)C [98] using supercells with 64 atoms and 48 atoms, respectively. It is possible that the depiction of high‐entropy disorder is inadequate due to the fact that the DFT calculations discussed before are usually limited to tiny systems.

Sampling potential configurations is the main obstacle to atomistic scale modeling of high‐entropy materials. In this context, approaches that are based on classical force‐field potentials have the potential to lower the amount of computational expense. In their study, Anand and colleagues investigated the competing roles that Δ*H* and Δ*S*_config_ play in the process of stabilizing the (Mg_0.2_Co_0.2_Ni_0.2_Cu_0.2_Zn_0.2_)O structure and its derivatives by employing the Buckingham interatomic potential [105]. Molecular dynamics simulations and optimization of the lattice parameters were performed using GULP and DL_POLY [106, 107]. A total of 23,536 configurations were generated using various methods, such as a genetic algorithm and random sampling. Important thermodynamic variables, including free energy, could be calculated using the sampled configurations. Figure 5a shows that Anand and colleagues found that the enthalpy cost of adding more cations to the system decreases with increasing cation numbers, but configurational entropy grows simultaneously [105]. This means that configurational entropy is becoming a more important factor in determining the stability of single‐phase oxide solutions, including solid solutions with various oxide components, as the number of cations increases.

High‐entropy perovskites have also been studied using classical calculations, along with oxides, borides, and carbides. Zhong et al. [46] explored the thermodynamic properties of LaMnO_3±δ_ and the mixing behavior of different defects using the CALPHAD method [108]. Specifically, they examined the effects of *p*_*O*2_, *T*, and composition on the mixing of particles and vacancies such as $V_{B}^{\prime \prime \prime }$ and $V_{\ddot{O}}$VO on three lattice sites of LaMnO_3 ± δ_ (Figure 5c,d) [46]. The authors proved that entropy is the primary factor in determining the mixing behavior of LaMnO_3 ± δ_ by establishing a connection between the mixing behaviors and the thermochemical characteristics.

Krawczyk et al. conducted research on the HEP (Gd_0.2_Nd_0.2_La_0.2_Sm_0.2_Y_0.2_)CoO_3_ and found that the bandgap measurements and ab initio calculations were in extremely close agreement with one another [75]. In addition, they found that the valence and conduction bands are significantly impacted by the O and Co atoms, respectively, while the Y and La atoms have a negligible impact. In contrast to the Moss–Burstein effect, which claims that the effective bandgap grows when in‐gap states are filled, the authors found that the bandgap decreases as temperature increases [109]. The higher spin‐state degeneracy of the Co^3+^ ions and electronic delocalization (Mott‐like transition) might be the cause of this contrasting pattern. In their study, Nguyen et al. observed that the catalytic activity of a La(Cr_1/6_Mn_1/6_Fe_1/6_Co_2/6_Ni_1/6_)O_3_ perovskite is highly connected to the Co^3+^/Co^2+^ ratio [20]. Specifically, they found that a larger ratio decreases the OER overpotential. Additionally, the scientists noted that *O is stable and *OO is unstable in this perovskite, leading them to conclude that OER proceeds via the adsorbate evolution rather than the lattice‐oxygen mechanism.

Additionally, computational descriptors can be used to forecast the synthesizability of high‐entropy materials [110]. For example, Sarker and colleagues presented a descriptor called the entropy‐forming ability (EFA) in order to determine whether or not high‐entropy carbides are capable of being synthesized [111]. For a certain unit‐cell size, EFA determines the spectrum of the N‐system's energy distribution above the ground state. Consequently, a narrow spectrum implies that the system is easily able to incorporate entropy, or unpredictability, at a limited temperature, while a broad range spectrum shows that the system favors an ordered phase structure with a high energy barrier over others. These specific description are quite good for quickly searching for high‐entropy materials. It provides a numerical evaluation of the tendency to produce a homogenous single phase for every combination, and it may assist in identifying the most promising candidates for experimental synthesis. Both of these features make it an ideal choice for the search. Discovering single‐phase, high‐entropy, refractory metal carbides by EFA proved that this method was efficient. In the end, six novel rock‐salt structured high‐entropy carbides were synthesized. The EFA showed great promise in speeding up the efficient search for high‐entropy systems, as all of these materials showed better mechanical characteristics with high hardness.

Furthermore, recent operando computational experiments suggest that surface reconstruction under electrochemical conditions is a key factor affecting catalytic activity [112]. DFT calculations and ab initio molecular dynamics simulations reveal that the high‐entropy surfaces can undergo reversible cation rearrangements and oxygen migrations during operation under OER conditions, generating catalytically active oxyhydroxide‐like surface layers that remain intact during bulk structural changes. This adaptive behavior is considered one of the key advantages of entropy‐stabilized perovskites over conventional perovskite catalysts. Consequently, future computational studies should integrate DFT, CALPHAD, machine learning, and operando simulations to establish quantitative correlations among configurational entropy, local atomic ordering, electronic structure, and catalytic performance.

## 5 HEPs in Energy and Environment Catalysis

**FIGURE 6:**
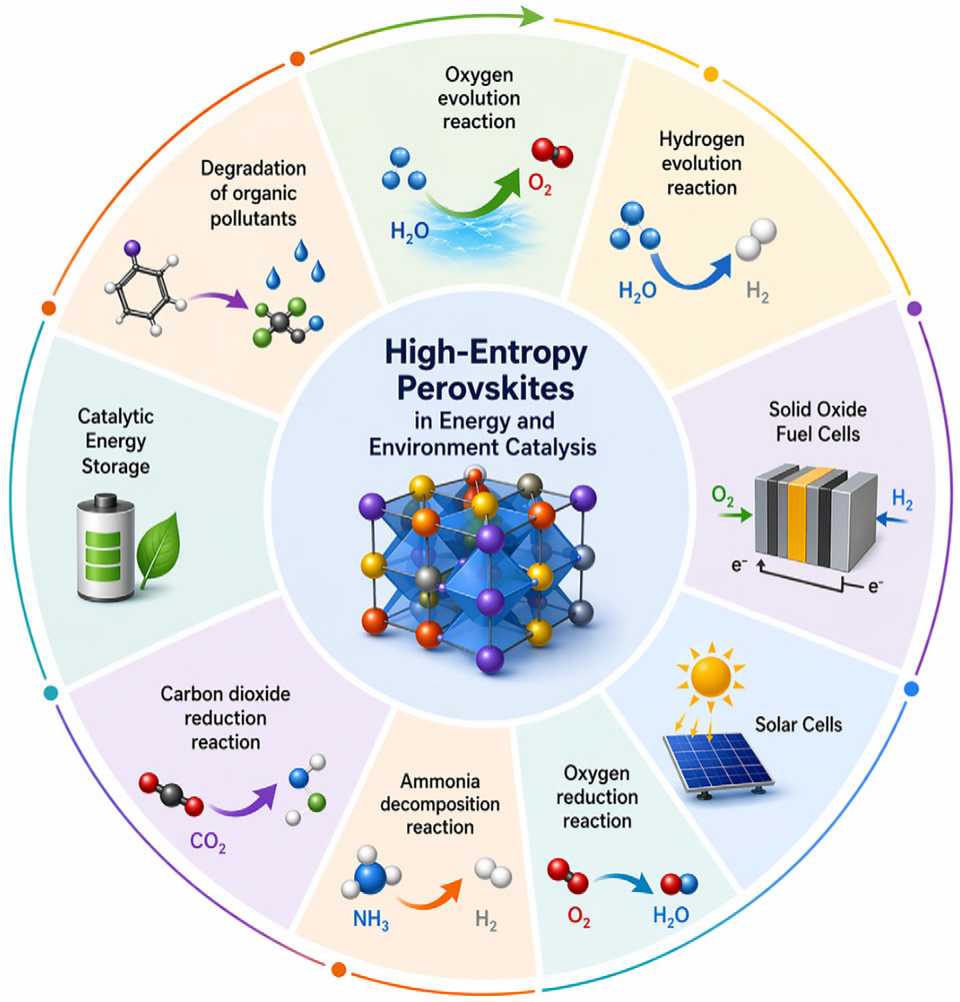
Schematic illustration of the potential use of HEPs in various energy and environmental catalytic applications.

Many studies have focused on HEP as electrocatalysts because of their diverse compositions, numerous surface electrical characteristics, high material utilization efficiency, and outstanding entropy stability [113, 114, 115, 116]. In addition, HEP catalysts have an excellent variety of active centers with optimized adsorption capabilities for various reaction intermediates, which significantly speed up reaction kinetics, and they exhibit outstanding intrinsic catalytic activity [117, 118]. Figure 6 depicts a schematic representation of the possible uses of HEPs in various energy‐ and environmental‐catalytic applications.

### 5.1 Electrocatalysis

#### 5.1.1 OER

The significance of water splitting is evident in a variety of technical fields, including fuel cells, solar energy production, and catalysis. By employing this method, easily accessible water may be converted into molecular oxygen at the anode and hydrogen at the cathode [119]. When it comes to electrochemical water splitting, the OER is crucial [120]. This is an extremely energy‐intensive stage, yet it is essential to the process's overall efficiency [121, 122]. Noble metal oxides (RuO_2_ and IrO_2_) exhibit exceptional OER activity, but their high cost, noble character, and low stability over time limit their use in practical applications [123, 124]. Consequently, there is an immediate requirement for a stable, non‐noble metal‐based OER catalyst that is robust, effective, cheap, and durable.

However, the OER has encountered a significant challenge in the formulation of O and OOH [76]. Fortunately, the increase in configurational entropy serves as a viable approach to promote intrinsic activity and develop the OER mechanism [125]. Additionally, HEM catalysts may have their composition and surface electronic structure adjusted, allowing for a varied atomic configuration. This configuration modifies the electronic coordination structure and adds defects, such as additional oxygen vacancies and metals [118, 126, 127].

Recently, HEPOs have developed as a potential class of electrocatalysts for OER due to their multicomponent cationic configuration, changeable electronic structure, and synergistic catalytic sites. By integrating five or more transition metals at the B‐site, HEPOs provide improved catalytic activity beyond usual scaling relationships, in contrast to ordinary ABO_3_ perovskites.

In one of the first investigations, Nguyen et al. showed that a lanthanum‐based HEPO, La(CrMnFeCo_2_Ni)O_3_, with multication disorder greatly increased OER activity. With an overpotential of 325 mV at 10 mA cm^−2^, the optimized composition La(CrMnFeCo_2_Ni)O_3_ outperformed single‐B‐site perovskites and demonstrated outstanding stability over 50 h [20]. This research demonstrated that compositional tailoring directly influences the catalytic activity of HEPOs. A‐site engineering in La_1−*x*_Sr_*x*_(CrMnFeCoNi)_0.2_O_3_ was recently reported by Wei et al. [128]. An overpotential of 330 mV at 10 mA cm^−2^ was produced by the optimized catalyst, La_0.3_Sr_0.7_(CrMnFeCoNi)_0.2_O_3_, which is 120 mV better than the undoped parent compound. The improvement in adsorption/desorption kinetics of OER intermediates and quicker charge transfer were both attributable to Sr‐induced high‐valence states, such as Co^4+^ and Fe^4+^. The catalyst demonstrated remarkable long‐term stability over 200 h, underscoring its practical application. Kante et al. delivered a mechanistic perspective on the intrinsic advantages of HEPOs [19]. The current densities of the LaCr_0.2_Mn_0.2_Fe_0.2_Co_0.2_Ni_0.2_O_3−*δ*_ model system were 17–680 times greater than those of individual single‐metal perovskites at the same overpotential. The “cocktail effect,” in which many redox‐active cations participate in the OER cycle simultaneously, enabling optimized adsorption energies for intermediates (*OH, O, OOH), is responsible for this remarkable improvement. The presence of mixed‐valence transition metals promotes rapid electron transfer, thereby accelerating reaction kinetics during water oxidation. These systems have the potential to circumvent the conventional limitations of volcano‐type activity that are observed in monometal oxides.

**FIGURE 7:**
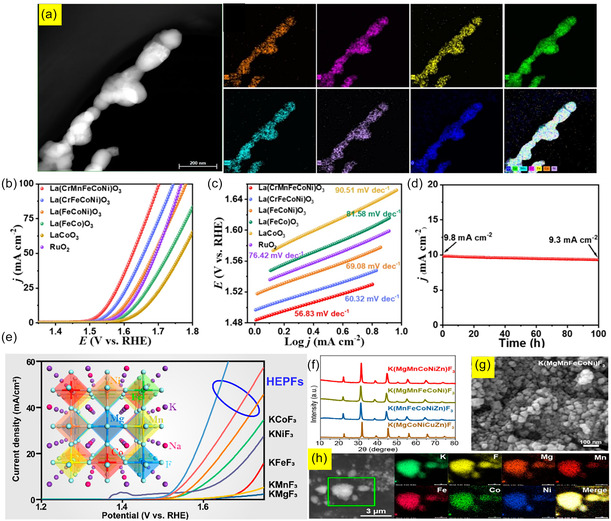
(a) EDX elemental composition maps of La(CrFeCoNi)O_3_, (b) LSV curves, (c) Tafel plots of perovskite oxides, and (d) chronoamperometry testing at 10 mA cm^−2^ of La(CrMnFeCoNi)O_3_. Reproduced with permission [129]. Copyright 2025, Elsevier. (e) High‐entropy perovskite fluorides, (f) XRD patterns of HEPFs, (g) SEM image, and (h) EDX mappings for K(MgMnFeCoNi)F_3_. Reproduced with permission [76]. Copyright 2020, American Chemical Society.

Another significant development in HEP OER catalysts was reported using microwave‐assisted synthesis of La(CrMnFeCoNi)O_3_ (Figure 7a–d) [129]. This equimolar HEPO with a B‐site structure demonstrated an overpotential of 316 mV at 10 mA cm^−2^ and a low Tafel slope of 56.8 mV dec^−1^, surpassing numerous conventionally synthesized counterparts. Furthermore, the catalyst demonstrated exceptional durability under alkaline conditions by maintaining sustained operation for more than 100 h. The study found that the lattice oxygen mechanism (LOM) is the main pathway for OER, which results in quicker oxygen exchange kinetics than the adsorbate evolution mechanism (AEM). This change in mechanism is critical since catalysts based on LOM often exhibit lower kinetic barriers and more intrinsic activity.

Another effective method for generating oxygen vacancies in HEPOs is the incorporation of anion doping, such as sulfur [130]. Using this method, especially the sulphurization process, creates more oxygen vacancies, which greatly improve OER performance by making the material more electrically conductive and providing more electrochemically active surface area. Lattice oxygen involvement is activated by oxygen vacancies, which allows traditional processes to overcome their kinetic constraints. As a result, vacancy engineering can improve kinetics by increasing active sites and tailoring the reaction route. Defect‐engineered La(FeCoNiCrMn)O_3−*x*_ systems include oxygen vacancies that contribute to unsaturated coordination, boost charge transfer, and improve ionic/electronic conductivity [131], resulting in faster reaction rates. Operando XAS studies on perovskite electrocatalysts have revealed reversible valence‐state transitions and oxygen‐vacancy evolution during OER, providing evidence for cooperative multication redox chemistry [132].

Aside from HEPOs, HEP fluorides have also been shown to be extremely active toward OER. Everyone knows that the alkaline OER works by first producing M–OH, then converting it to M–O and M–OOH, and lastly producing O_2_ while simultaneously recovering the original active sites. The primary concern is that the formation of M–OOH is more challenging due to the strong binding of oxygen to M, whereas the formation of M–O is more difficult due to the feeble binding of oxygen to M. Because of its high electronegativity, which typically results in weak metal–F bonds to create greater iconicity, doping metal oxides with fluorine (F) is an attractive technique for producing superior electrocatalytic OER activity. In terms of improving OER, ABF_3_‐type perovskite fluorides should be a better choice than perovskite oxides due to their intrinsic 3D diffusion channels and abundance of F sites. Based on this, Wang and colleagues developed HEP fluorides by synthesis and used them as sophisticated electrocatalysts for organic electrolysis reactions [76]. As shown in Figure 7e–h, the ABF_3_ type HEP fluorides showed remarkable electrocatalytic OERs, boasting an exceptionally lower overpotential of 314 mV at 10 mA cm^−2^. Additionally, the low charge‐transfer resistance, rapid mass transfer, and abundance of active sites are the sources of the exceptional OER performance. Table 1 shows further research on HEPs as OER electrocatalysts.

**TABLE 1:** Electrocatalytic performance of HEPs in the OER.

HEP composition	Electrolyte	**Overpotential @10 mA cm** ^**−2**^	**Tafel slope, mV dec** ^**−1**^	Stability	Reference
La(CrMnFeCoNi)_0.2_O_3_	1 M KOH	450	64	200 h@ 10 mA cm^−2^	[128]
La_0.3_Sr_0.7_(CrMnFeCoNi)_0.2_O_3_	1 M KOH	330	55	200 h@ 10 mA cm^−2^	[128]
La_0.7_Sr_0.3_Co_0.2_Mn_0.2_Fe_0.2_Ni_0.2_Al_0.2_O_3−*x*_	1 M KOH	339	73.9	100 h@ 10 mA cm^−2^	[133]
La(CrMnFeCo_2_Ni)O_3_	0.1 M KOH	325	51.2	50 h@ 10 mA cm^−2^	[20]
(CoNiMnZnFe)_3_O_3.2_	1 M KOH	336	47.5	20 h	[134]
La_0.7_Sr_0.3_Fe_1−*x*_Ni_*x*_O_3−*δ*_	0.1 M KOH	320	35	50 h	[135]
La(CrMnFeCoNi)O_3_	1 M KOH	316	56.83	100 h	[129]
La(Cu_0.2_Ni_0.2_Co_0.2_Fe_0.2_Nb_0.2_)O_3−*δ*_	0.1 M KOH	1868	120	—	[136]
La(MnFeCoNiCu)O_3_	1 M KOH	303	43	100 h@ 10 mA cm^−2^	[137]
S/LMO‐E	1 M KOH	314	47	20 h@ 10 mA cm^−2^	[130]
Ba_0.33_Sr_0.67_Co_0.33_Ti_0.165_Ru_0.165_Sb_0.33_O_3_	1 M KOH	286	49	16 h@ 10 mA cm^−2^	[138]
La(NiCuCoFeMn)O_3_	1 M KOH	328	53	100 h	[139]
La_0.6_Sr_0.4_Co_0.8_Fe_0.2_O_3_	1 M KOH	353	63	1000‐cycles	[140]
(La_0.6_Sr_0.4_)(Co_0.2_[FeMnNiMg]_0.8_)O_3_	1 M KOH	320	45	20 h	[141]
La(Co_0.2_Ni_0.2_Fe_0.2_Cr_0.2_Al_0.2_)O_2.9_F_0.1_	1 M KOH	302	47.4	40 h@ 10 mA cm^−2^	[142]
La_0.6_Sr_0.4_Co_0.8_Fe_0.1_Mn_0.1_O_3−*δ*_	1 M KOH	343	63	20 h@ 10 mA cm^−2^	[143]
La(Co_0.2_Mn_0.2_Fe_0.2_Ni_0.2_Cu_0.2_)O_3_	1 M KOH	309	56.49	72 h@ 10 mA cm^−2^	[144]
Ba_0.5_Sr_0.5_Co_0.8_Fe_0.2_O_3−*δ*_	1 M KOH	360	61	10 h@ 10 mA cm^−2^	[145]
La(CrMnFeCoNi)_1/5_O_3_	1 M KOH	313	47.4	10 h@ 10 mA cm^−2^	[146]
LaCr_0.2_Mn_0.2_Fe_0.2_Co_0.2_Ni_0.2_O_3−*δ*_	1 M KOH	450	51	13.8 h	[19]
K_0.8_Na_0.2_(MgMnFeCoNi)F_3_	1 M KOH	314	55	10 h	[76]
La_0.5_Sr_0.5_Mn_0.15_Fe_0.15_Co_0.4_Ni_0.15_Cu_0.15_O_3_	1 M KOH	309	81.1	20 h@ 10 mA cm^−2^	[147]
La_0.6_Ca_0.4_(CrMnFeCo_2_Ni)O_3_	1 M KOH	340	57.41	200 h@ 10 mA cm^−2^	[148]
(MnFeNiCoCr)_3_O_4_	1 M KOH	323	56	20 h@ 10 mA cm^−2^	[149]

#### 5.1.2 HER

In contrast to their widespread use in oxygen evolution catalysis, HEP‐based materials for the HER are still largely unexplored. One main reason for this shortage is the difficulty in improving the hydrogen adsorption free energy (Δ*G*_H_) in systems with multiple cations in perovskites, as well as the naturally slow HER kinetics on oxide surfaces. However, recent research has shown that integrating multiple cations into a single perovskite lattice can generate distinctive electronic interactions, thereby providing new opportunities for HER catalysis.

**FIGURE 8:**
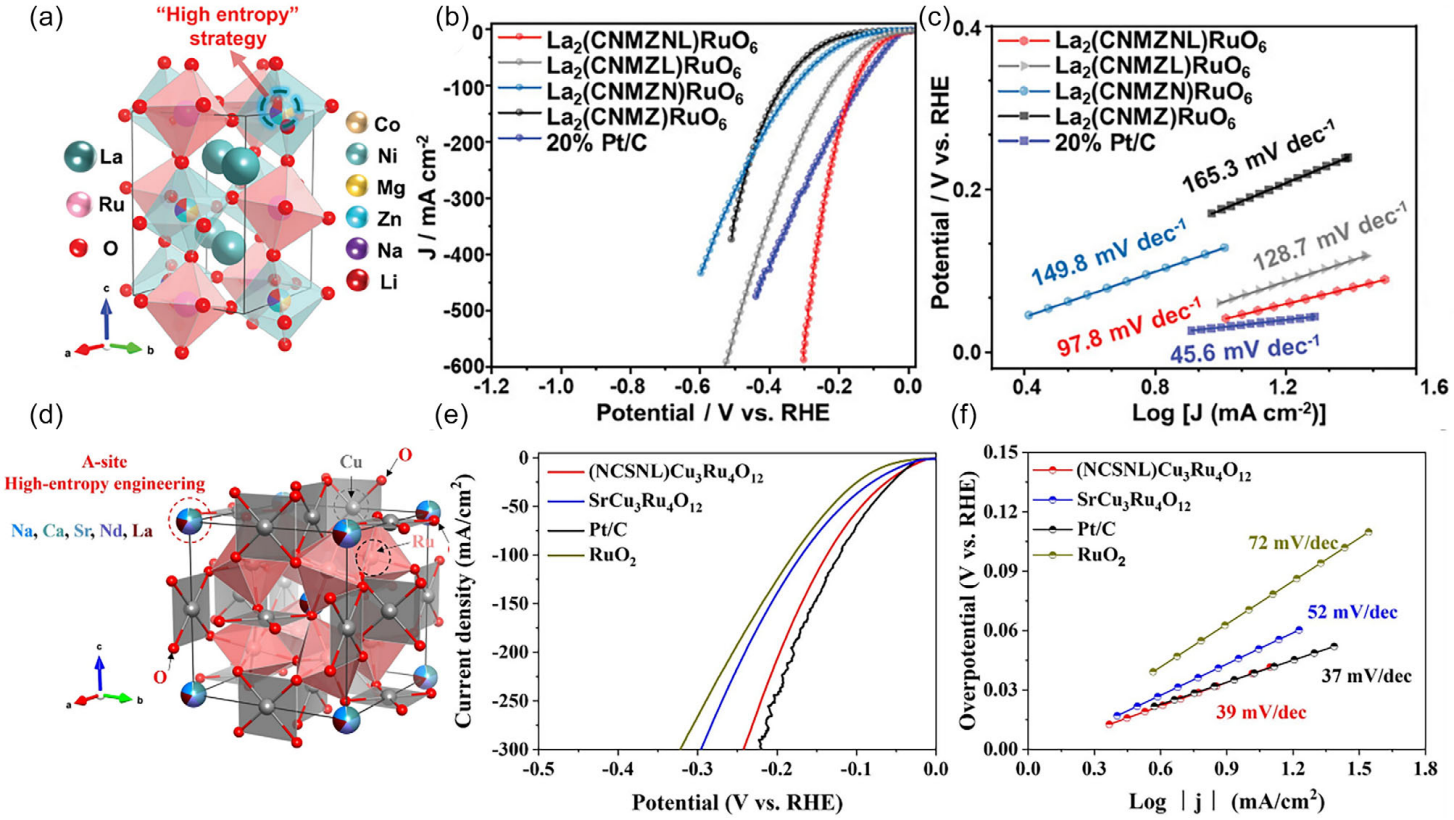
(a) Schematic diagram of the crystal structure of high entropy, (b) LSV curves, and (c) Tafel slopes of the prepared electrocatalyst. Reproduced with permission [150]. Copyright 2024, John Wiley and Sons. (d) Schematic illustration of the crystal structure, (e) LSV curves, and (f) Tafel slopes of the prepared electrocatalysts. Reproduced with permission [151]. Copyright 2025, Elsevier.

Sun et al. described the high‐entropy double perovskite La_2_(Co_1/6_Ni_1/6_Mg_1/6_Zn_1/6_Na_1/6_Li_1/6_)RuO_6_, which is considered to be one of the most impressive examples (Figure 8a) [150]**.** This catalyst demonstrates an overpotential of 40.7 mV at 10 mA cm^−2^ in 1 M KOH (Figure 8b,c), which is significantly lower than that of advanced noble metal catalysts. Additionally, it exhibits exceptional durability for more than 82 h without any deterioration. The remarkable results are explained by the super‐exchange interactions that are created by alkali metals. These interactions enhance electron transport from Ru to Co/Ni sites, which in turn optimizes hydrogen adsorption and lowers the water dissociation barrier. In contrast to traditional perovskites, this research shows that entropy‐driven electronic coupling can greatly improve HER kinetics.

Further advancements have been made by structurally controlling HEPs. A recent investigation carried out by Wenbo Li et al. [151] on an A‐site ordered high‐entropy quadruple perovskite, (Na_0.2_Ca_0.2_Sr_0.2_Nd_0.2_La_0.2_)Cu_3_Ru_2_O_12_, showed an even lower overpotential of around 36 mV at 10 mA cm^−2^, along with a small Tafel slope of approximately 39 mV dec^−1^ (Figure 8d–f). These values position it among the non‐Pt HER catalysts that have been reported to have the best performance so far. The improved activity is due to precise A‐site entropy engineering, which alters the electronic structure of Ru active centers and makes it easier for intermediates to adhere to the surface (H, OH). This system exhibits a nearly twofold increase in catalytic efficiency when contrasted with conventional Ru‐based perovskites, which commonly exhibit an overpotential of over 80 mV. This underscores the significance of lattice ordering and compositional complexity.

Additionally, HEPs have shown potential for photocatalytic hydrogen evolution, in addition to their use in electrocatalysis. For instance, HEPs based on Ba–Sr, such as (Ba_1/2_Sr_1/2_)(Ti_1/3_Zr_1/3_Hf_1/3_)O_3_, demonstrate efficient hydrogen generation without the need for cocatalysts [152]. By virtue of their improved charge carrier mobility and optimized band topologies, these materials are able to effectively split water when exposed to light. Despite the fact that their HER rates are typically lower than those of electrochemical systems, they underscore the adaptability of HEPOs in various hydrogen production pathways. In addition to their activity, HEPOs offer a significant advantage in HER‐related systems by enhancing stability through entropy engineering. Multicomponent perovskites have greater structural stability and resistance to deterioration under electrochemical conditions [153]. The high configurational entropy is responsible for this stability, as it prevents phase segregation and ensures a uniform distribution of active sites. Although this study focuses on electrolysis systems, the same idea applies to HER catalysis, where long‐term durability (>100 h) is critical for practical applications.

Recently, Zhao et al. indicated that porous rare‐earth‐based HEPO nanosheets can simultaneously promote both HER and OER under acidic conditions [154]. These porous nanostructures exhibited only 49 mV overpotential for HER at 10 mA cm^−2^, along with excellent operational stability at industrial‐scale current densities. The enhanced performance originated from abundant oxygen vacancies, low‐valence active sites, and improved mass transport within the porous framework. Such findings further highlight the potential of HEPs as next‐generation electrocatalysts for overall water splitting and renewable hydrogen production. In addition, CdS/La(Co_0.2_Ni_0.2_Fe_0.2_Cr_0.2_Mn_0.2_)O_3_ HEP hollow core–shell heterojunction reported a novel hollow core–shell heterojunction for efficient photocatalytic water splitting by Huang et al. [155]. The material, synthesized through a hydrothermal–annealing–chemical route, integrates CdS nanoparticles with a five‐element HEP oxide, creating strong polymetallic ionic coupling and an efficient heterojunction interface. This synergistic architecture accelerates charge transport, suppresses carrier recombination, and enhances H^+^ adsorption and H_2_O_2_ decomposition, leading to dramatically improved photocatalytic activity. As a result, the catalyst exhibited nearly 110‐fold higher HER performance than LaCoO_3_ and ∼100‐fold higher activity than pristine CdS, achieving an H_2_ evolution rate of 285.73 μmol·g^−1^·h^−1^ with excellent stability. The hollow core–shell design further increases active‐site exposure while mitigating photocorrosion, highlighting the strong potential of HEP heterostructures for sustainable overall water splitting. Continued work on compositional optimization, defect engineering, and hybridization with conductive frameworks is likely to improve their performance for real‐world water‐splitting applications.

#### 5.1.3 ORR

The ORR has attracted significant interest from scientists all around the globe because of its vital role in fuel cell technological advancements. Oxygen molecules are reduced to water or other species in this process. The slow kinetics of this material, however, make it difficult to use in fuel cells and metal‐O_2_ batteries [156, 157, 158]. Platinum‐based NPs have previously been utilized as highly active ORR electrocatalysts; however, their extensive implementation is impeded by their exorbitant cost and insufficient natural resources. HEPs have recently developed as a potential class of electrocatalysts for the ORR, owing to their distinctive configurational entropy, multicomponent synergy, and adjustable electronic structures. There are very few reports on the use of HEPs for ORR reactions. A highly disordered yet thermodynamically stabilized lattice is produced by HEPs by including five or more cations, usually at the B‐site, in contrast to normal ABO_3_ perovskites. Crucial for optimizing ORR kinetics, this compositional complexity allows for the fine‐tuning of adsorption energies for oxygen intermediates (*O_2_, *OOH, *OH). In a recent investigation, Liang and his research group emphasized the attractiveness of high‐entropy materials for fuel cells and metal‐air batteries due to their increased poisoning resistance and wider distribution of active sites compared to single‐ or binary‐metal systems [159].

**FIGURE 9:**
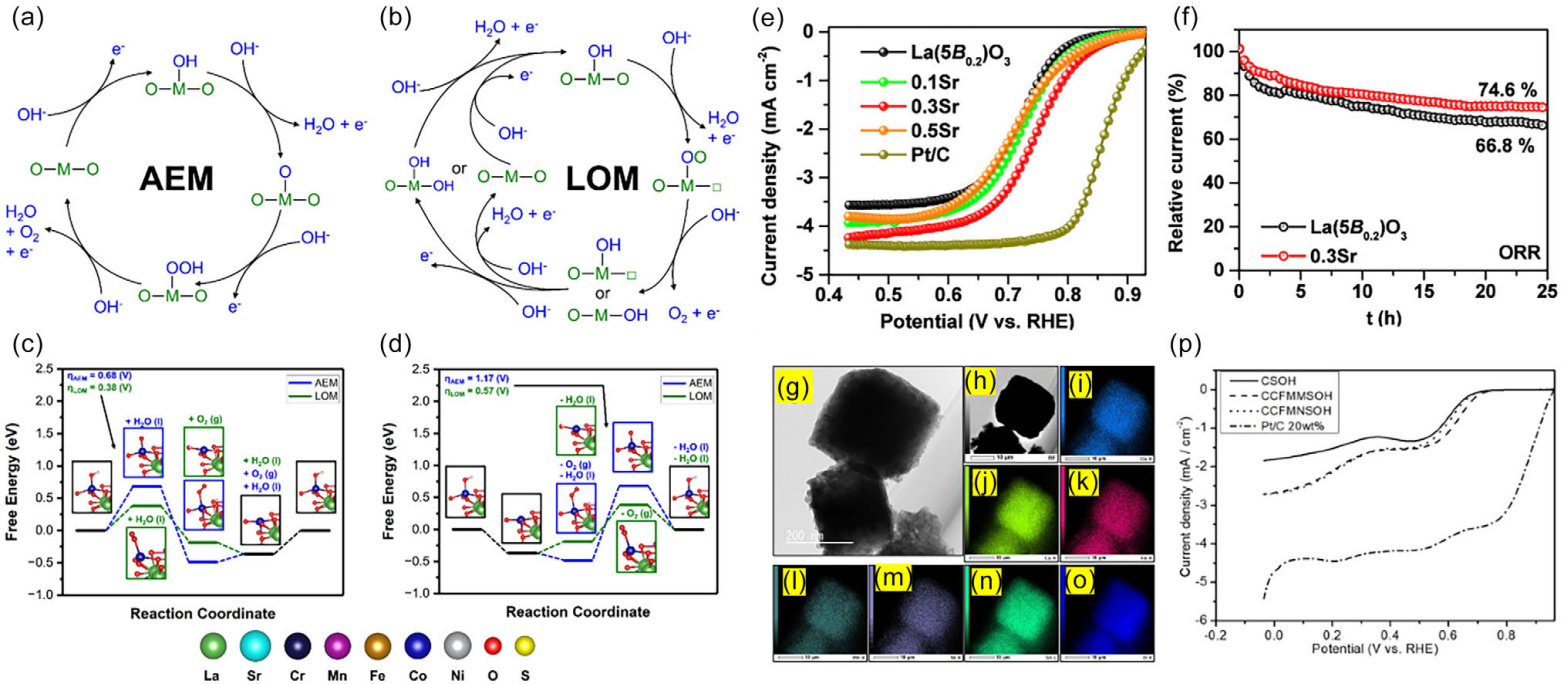
(a) AEM and (b) LOM pathways of OER. The open square indicates where the oxygen vacancy is close to the active site. The reverse reaction of OER is ORR. Free energy illustrations for (c) OER and (d) ORR. Reproduced with permission [160]. Copyright 2025, Elsevier. (e) ORR polarization curves. (f) Chrono‐current response of La_0.7_Sr_0.3_(Cr_0.2_Mn_0.2_Fe_0.2_Co_0.2_Ni_0.2_)O_3_ at 0.5 V versus RHE. Reproduced with permission [161]. Copyright 2025, American Chemical Society. (g) TEM image, (h–o) elemental mapping images of (Co_0.2_Cu_0.2_Fe_0.2_Mn_0.2_Mg_0.2_)Sn(OH)_6_, and (p) ORR curves of prepared catalysts measured at 0.1 M KOH aqueous solution. Reproduced with permission [162]. Copyright 2024, MDPI.

An important development in recent research is the identification of multiactive‐site behavior in HEPs. A high‐entropy system distributes catalytic functions among many cations, in contrast to ordinary perovskites where a single transition metal dominates ORR activity. For instance, a recent study revealed that the primary ORR‐active centers in an S‐doped HEP (La_0.8_Sr_0.2_(CrMnFeCoNi)O_3_) are Mn, Fe, and Co, while Ni serves as a secondary electronic modulator [160]. Electronic delocalization induced by multicomponent cation interactions improves electrical conductivity and lowers charge‐transfer resistance. The coexistence of multiple redox‐active centers facilitates efficient electron transport and promotes oxygen activation. The research has also highlighted the importance of LOM in ORR pathways. Although high‐entropy compositions can activate dual pathways, ORR on perovskites typically follows an AEM (Figure 9a–d). This separation of catalytic functions is crucial because it enables the optimization of three fundamental processes in the ORR process, including O_2_ adsorption, O—O bond breakage, and OH^‐^ desorption in a single material at the same time. This system demonstrated a half‐wave potential of approximately 0.731 V versus RHE, which is considerably higher than the half‐wave potential of many single‐component perovskites (typically between 0.65 and 0.70 V). Compared to Pt/C, this value is relatively lower.

The multicomponent oxide La(Cr_0.2_Mn_0.2_Fe_0.2_Co_0.2_Ni_0.2_)O_3_ is a notable example of a HEP‐based ORR catalyst. In their work, Fu et al. utilized a hole‐doping technique to develop La_0.7_Sr_0.3_(Cr_0.2_Mn_0.2_Fe_0.2_Co_0.2_Ni_0.2_)O_3_ [161]. This material exhibited a substantial increase in ORR activity, as evidenced by a reduced overpotential and a narrow bifunctional gap of approximately 0.88 V between the ORR half‐wave potential and the OER potential at 10 mA cm^−2^ (Figure 9e,f). The entropy‐engineered system demonstrates better catalytic reversibility and kinetics when benchmarked against traditional perovskites like La_0.6_Sr_0.4_CoO_3_, which typically exhibit larger potential gaps (>1.0 V). The authors attributed this enhancement to the optimized *e*_g_ orbital filling (∼1.2), which is close to the ideal value for oxygen electrocatalysis. Consequently, the adsorption/desorption equilibrium of oxygen intermediates was improved. Chae et al. also made a significant contribution by synthesizing HEP hydroxides ((Co_0.2_Cu_0.2_Fe_0.2_Mn_0.2_Mg_0.2_)Sn(OH)_6_) as bifunctional ORR/OER catalysts (Figure 9g–o) [162]. In alkaline conditions, their material showed a half‐wave potential (*E*_1/2_) that was competitive with several catalysts made of nonprecious metals, approaching ∼0.80–0.84 V versus RHE (Figure 9p). The catalyst also showed great longevity, keeping more than 90% of its original current after several cycles. This is better than many common transition‐metal oxides. The activity disparity is relatively small when compared to Pt/C (*E*_1/2 _≈ 0.85–0.87 V), but the HEP system offers superior long‐term stability and reduced cost, underscoring its practical relevance. HEPs are not only competitive with state‐of‐the‐art catalysts but also offer a versatile platform for next‐generation oxygen electrocatalysis.

#### 5.1.4 Ammonia Decomposition Reaction

**FIGURE 10:**
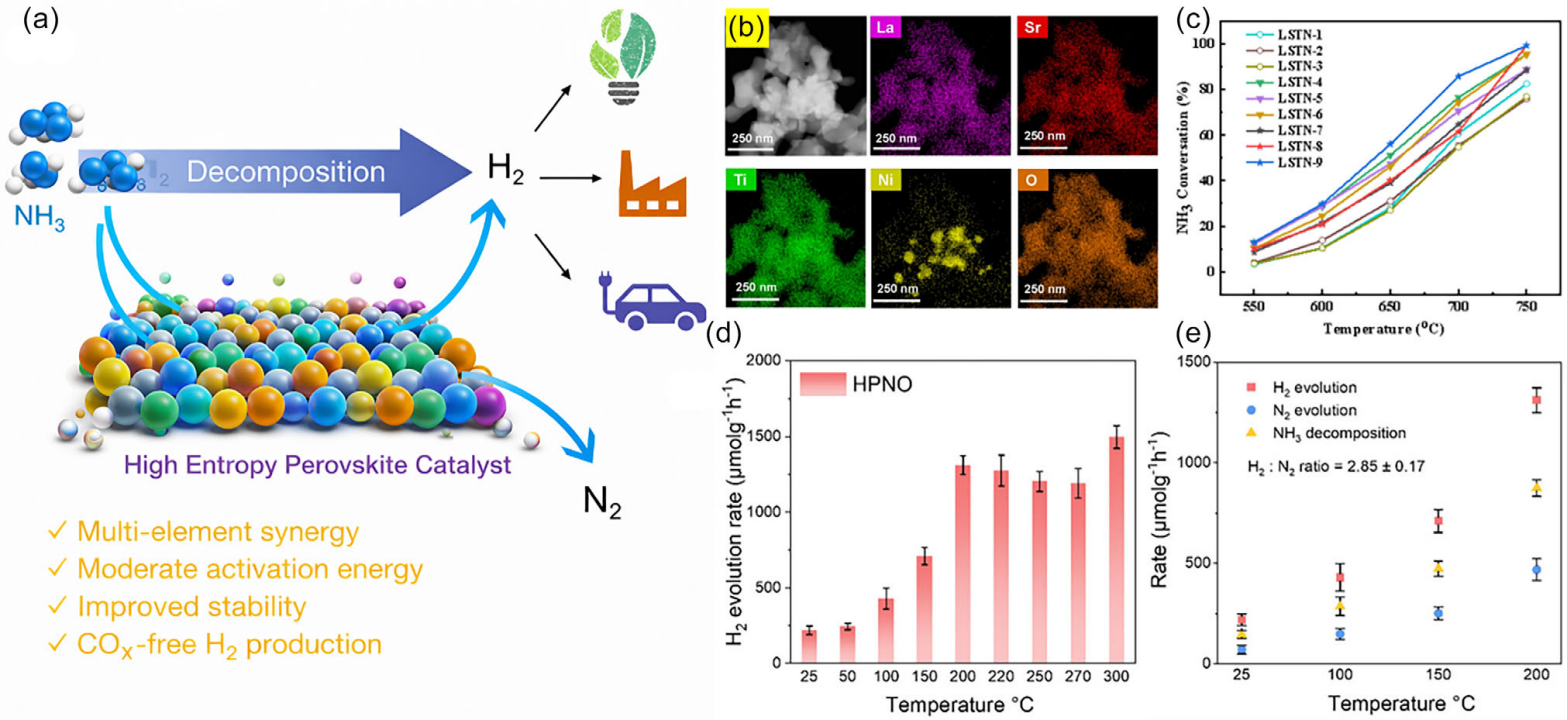
(a) Mechanism of ammonia decomposition reaction facilitated by HEP Catalysts. (b) Elemental mapping images of the La_0.4_Sr_0.5_Ti_0.94_Ni_0.06_O_3−*δ*_ catalyst. (c) Ammonia conversion of La_0.4_Sr_0.5_Ti_0.94_Ni_0.06_O_3−*δ*_ catalysts. Reproduced with permission [165]. Copyright 2024. American Chemical Society. (d) Photocatalytic ammonia decomposition reaction of protonated layered perovskite (HPrNb_2_O_7_) under different temperatures and (e) the rate of H_2_ evolution, N_2_ evolution, and NH_3_ decomposition at varying temperatures. Reproduced with permission [166]. Copyright 2025, John Wiley and Sons.

Although hydrogen has great potential as a clean, efficient fuel for the future, its very low density makes it difficult to transport and store [103]. Hydrogen storage in chemical compounds is the key to overcoming these challenges. Therefore, NH_3_ has the ability to transport and store hydrogen more efficiently than gaseous hydrogen because of its high energy density [163, 164]. In addition, no greenhouse gases are released into the environment when gaseous NH_3_ is decomposed to produce hydrogen (Figure 10a) [167]. The breakdown of ammonia can produce hydrogen, but only with the help of an effective catalyst. Although Ru is far more efficient than other metal catalysts at accelerating the breakdown of NH_3_, its high cost and limited availability prevent its widespread application [168].

More recently, entropy‐engineered perovskite systems have been the focus of research. These systems create substantial configurational entropy by including numerous cations into a single lattice. Although there is a lack of extensive research on HEPs specifically for ammonia breakdown, we can still understand their catalytic principles by drawing comparisons to both traditional perovskites and HEAs. As an example, Ali et al. showed that, compared to monometallic catalysts, Co–Ni bimetallic catalysts based on LaMnO_3_ perovskites converted 95% of ammonia at 535°C (GHSV = 20,400 mL g^−1 ^h^−1^) [169]. The enhanced performance was ascribed to the electronic synergy between Co and Ni, which resulted in an increase in active site density and an improvement in electron transfer. Multielement synergism modifies the electronic density around active metal centers, facilitating N—H bond cleavage. The lattice distortion and defect generation promote rapid electron transfer and increase the density of catalytically active sites. In order to take this idea a step further, HEPs incorporate several B‐site elements (such as Co, Ni, Fe, Mn, and Cu) that allow for the creation of multisite catalytic pathways and the optimization of adsorption energies for NH_*x*_ intermediates. The significance of A‐site deficit and oxygen vacancy generation in improving catalytic activity was emphasized by Chen et al., who found that perovskites based on defect‐engineered La–Sr–Ti (La_0.4_Sr_0.5_Ti_0.94_Ni_0.06_O_3−*δ*_) converted around 86% of NH_3_ at 700°C (Figure 10b,c) [165]. These investigations proved that the ammonia decomposition driven by perovskite catalysts is significantly dependent on defect chemistry and exsolved metal nanoparticles.

The existence of numerous transition metals at the B‐site is expected to result in a uniform distribution of adsorption energies, surpassing the classic Sabatier limitations observed with single‐metal catalysts. In this context, Chen et al. observed that HEP (LaSrNiCoMnFeCuO_3_) porous nanotubes achieved about 100% Faradaic efficiency in reactions involving ammonia [170]. This is because of charge redistribution and multisite synergy. This work concentrated on nitrate reduction; nevertheless, the fundamental concept of entropy‐induced electrical modulation is directly relevant to ammonia breakdown. The significance of oxygen vacancies and lattice oxygen mobility is another crucial component in the ammonia breakdown process using perovskite‐based catalysts. Zhang et al. discovered that protonated layered perovskites may promote ammonia activation via surface defect sites, especially under thermally aided catalytic conditions (Figure 10d,e) [166]. The presence of numerous cations in HEPs destabilizes the lattice intrinsically, which in turn promotes the formation of oxygen vacancies and enhances redox flexibility. The rate‐determining phase of N—H bond dissociation, which is usually the bottleneck in ammonia breakdown, can be accelerated in this defect‐rich environment. Additionally, entropy stabilization is able to prevent phase segregation at elevated temperatures, which guarantees long‐term catalytic stability—a distinct advantage over conventionally doped perovskites.

#### 5.1.5 Carbon Dioxide Reduction Reaction

There is an urgent need to lower the amount of greenhouse gas entering the atmosphere due to the significant increase in anthropogenic CO_2_ emissions from increased fossil fuel burning activities [171]. By converting CO_2_ into useful chemicals such as methane, ethylene, and ethanol, these methods provide possible ways to address the problem of excessive CO_2_ emissions [172, 173, 174]. Consequently, the electrocatalytic CO_2_ reduction reaction (CO_2_RR) has been utilized to develop a range of chemical fuels using a number of catalysts, including carbon‐based materials, metal sulfides, and oxides of metals. It is worth noting that low faradaic efficiency and poor selectivity are major concerns. Problems with producing enough carbonaceous products are caused by catalysts that are not active enough in C–C coupling processes. As a result, it is critical to construct highly efficient and selective electrocatalysts that enable CO_2_RR.

**FIGURE 11:**
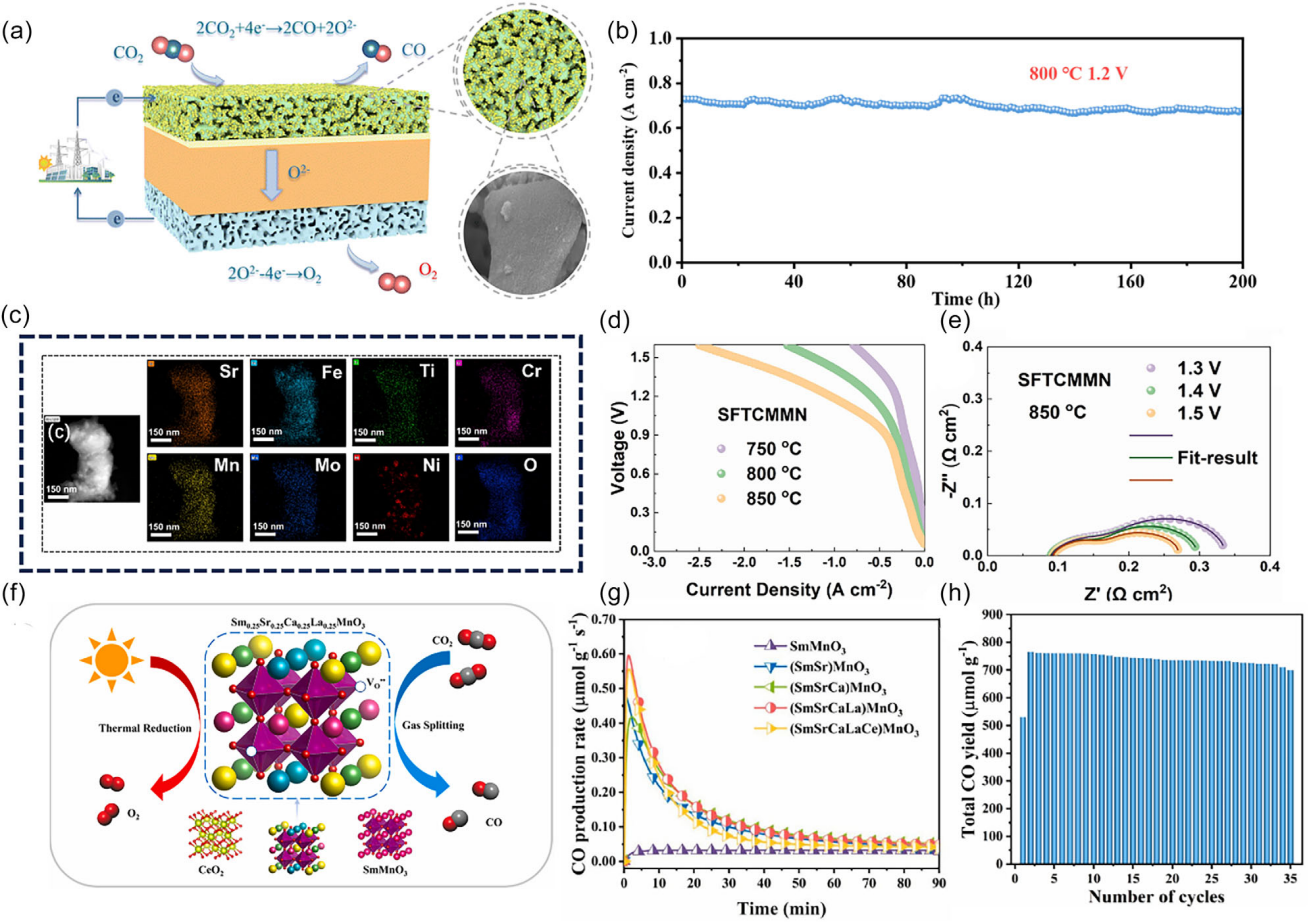
(a) Schematic representation of the SOEC for CO_2_ electrolysis. (b) Constant voltage CO_2_ electrolysis curve of the NiFe@Sr_2_Fe_0.4_V_0.4_Mo_0.4_Ni_0.4_Ti_0.6_O_6−*δ*_ based SOEC at 1.2 V and 800°C. Reproduced with permission [175]. Copyright 2024, Royal Society of Chemistry. (c) EDS mapping of Sr_2_Fe_1_Ti_0.2_Cr_0.2_Mn_0.2_Mo_0.2_Ni_0.2_O_6−*δ*_, (d) *I*–*V* curves of Sr_2_Fe_1_Ti_0.2_Cr_0.2_Mn_0.2_Mo_0.2_Ni_0.2_O_6−*δ*_ ‐ Ce_0.9_Gd_0.1_O_1.95_ fuel electrode at 750°C–850°C, and (e) EIS curves of Sr_2_Fe_1_Ti_0.2_Cr_0.2_Mn_0.2_Mo_0.2_Ni_0.2_O_6−*δ*_‐Ce_0.9_Gd_0.1_O_1.95_ fuel electrode at 850°C under various electrolytic voltage. Reproduced with permission [176]. Copyright 2025, Elsevier. (f) Schematic representation of the reaction of Sm_0.25_Sr_0.25_Ca_0.25_La_0.25_MnO_3_ HEP, (g) CO to O_2_ ratio of different catalysts, and (h) the total CO production of (SmSrCaLa)MnO_3_ after 35 redox cycles at 1350°C and 1100°C. Reproduced with permission [177]. Copyright 2024, Elsevier.

Recently, HEPs have emerged as a promising class of materials for the CO_2_RR. Their remarkable catalytic activity is derived from configurational entropy, which allows for adjustable electronic structures, stabilizes single‐phase structures, and increases oxygen vacancy concentration. As an alternative to traditional perovskites and metal‐based catalysts, HEPs offer desirable properties that make them ideal for CO_2_ adsorption, activation, and conversion. Zhu et al. examined metal/HEP heterointerfaces for CO_2_ electrocatalytic reduction (Figure 11a). Their research showed that using metallic active sites to couple HEPs greatly accelerates CO_2_RR kinetics by leveraging charge transfer and intermediate adsorption energies. Heterostructures exhibit lower polarization resistance and higher current density than single‐phase perovskites, suggesting faster reaction kinetics (Figure 11b) [175]. A La(FeCoNiCrMn)O_3_ HEPO was constructed by Wang et al. for the photocatalytic reduction of CO_2_. The catalyst obtained a CO evolution rate of 131.8 μmol g^−1 ^h^−1^, which is much higher than the typical rate of many conventional perovskite photocatalysts (<50 μmol g^−1 ^h^−1^). The improvement in charge separation and CO_2_ adsorption was attributed to synergistic interactions among multiple metals [178]. Further improvements in electrochemical CO_2_RR performance were demonstrated by a study by Wang et al., study on a B‐site HEP Sr_2_FeTiCrMnMoNiO_6−*δ*_ (SFTCMMN) (Figure 11c). This material achieved a remarkable current density of 2.10 A cm^−2^ at 1.5 V in CO_2_ electrolysis mode and remained stable for 150 h without carbon deposition. This is a huge improvement in performance and longevity over previous systems. By adding more active sites and facilitating electron transport, in situ exsolved Ni nanoparticles significantly improved catalytic performance (Figure 11d,e) [176]. Additionally, an alternative strategy for thermochemical CO_2_ splitting has involved investigating HEPs. Recent research has shown that entropy engineering improves CO yield relative to benchmark CeO_2_ systems by reducing the energy of oxygen vacancy formation and increasing lattice oxygen movement (Figure 11f–h). This work proves that HEPs can convert solar‐driven CO_2_ more efficiently than conventional oxides, even though the precise rates of CO generation are temperature dependent [177]. Recently, AB_2_O_6_‐type layered HEOs have attracted attention because their multicomponent cationic configuration can simultaneously optimize light absorption, charge separation, surface basicity, and CO_2_ adsorption. A recent study by Hai et al. introduced a polymorphic AB_2_O_6_‐type HEO, (Cs_1/7_Ba_4/7_Bi_2/7_)(Nb_1/2_Ta_1/2_)_2_O_6_, containing layered perovskite and pyrochlore structural motifs for photocatalytic CO_2_ reduction using microalgae as a carbon‐negative sacrificial scavenger [179]. In this system, Cs and Ba were incorporated to enhance surface basicity and CO_2_ chemisorption, while Bi lone‐pair electrons promoted local polarization and charge separation. Furthermore, a recent report demonstrated the feasibility of simultaneous CO_2_ conversion and plastic upcycling using multifunctional catalytic systems. This concept opens a highly attractive research direction for HEP materials because their tunable electronic structures, compositional flexibility, and broad adsorption‐energy distributions may enable coupled catalytic reactions involving both carbon dioxide activation and polymer‐derived intermediates. Future research could therefore focus on designing multifunctional HEP catalysts capable of integrating carbon‐neutral fuel production with plastic waste valorization, offering a sustainable pathway toward circular carbon technologies [180]. The performance of HEP as catalysts for CO_2_RR is presented in Table 2.

**TABLE 2:** Performance of HEP as catalysts for CO_2_RR.

Material	System	Current density	Stability	Other performance	Key findings	DOI
La_1/5_Sr_1/5_Pr_1/5_Ba_1/5_Ca_1/5_FeO_3−*δ*_	SOEC	2.14 A cm^−2^ @1.5 V	120 h	High CO_2_ conversion	A‐site entropy → vacancy enhancement	[181]
Sr_2_FeTiCrMnMoNiO_6−*δ*_	SOEC	2.10 A cm^−2^ @ 1.5 V	150 h	No carbon deposition	Ni exsolution improves activity	[176]
Sr_2_Fe_0.8_Mo_0.4_Co_0.2_Ni_0.2_Cu_0.2_Ru_0.2_O_6−*δ*_	SOEC	2.08 A cm^−2^ @ 1.5 V	Stable	>90% CO selectivity	Electronic structure tuning boosts kinetics	[182]
Pr_0.5_Ba_0.5_Mn_0.2_Fe_0.2_Co_0.2_Ni_0.2_Cu_0.2_O_3−*δ*_	SOEC	1.21 A cm^−2^ @2.0 V	60 h	Dual CO_2_RR + OER	Alloy (CoFeNiCu) exsolution	[183]
(LaSrPrBaCaGd)_2_Fe_1.5_Mo_0.5_O_6−*δ*_	SOEC	1.34 A cm^−2^ @ 1.5 V	400 h	Improved activity	A‐site entropy improves durability	[153]
Pr_1/6_La_1/6_Nd_1/6_Ba_1/6_Sr_1/6_Ca_1/6_CoO_3−*δ*_	R‐PCECs.	−1.95 A cm^−2^ @ 1.3 V	1000 min	ORR and OER	Excellent structural stability and oxygen catalytic activity	[184]
Pr_0.8_Sr_1.2_(CuFe)_0.4_Mo_0.2_Mn_0.2_Nb_0.2_O_4−*δ*_	SOEC	1.95 A cm^−2^ @ 1.5 V	200 h	Core‐shell Structure	High Stability and scalability Potential	[185]
La_0.2_Pr_0.2_K_0.2_Sr_0.2_Ca_0.2_Fe_0.8_Ni_0.2_O_3−*δ*_	SOEC	1.1 A⋅cm^−2^ @ 1.5 V	200 h	Doping strategy	K‐doping induced oxygen vacancy formation, improves CO_2_ adsorption and activation	[186]
Ni@Sr_2_Fe_1.0_Ti_0.2_Cr_0.2_Mn_0.2_Mo_0.2_Ni_0.2_O_6−*δ*_	SOEC	1.91 A cm^−^^2^ @ 1.5 V	160 h	In situ exsolution	Enhances the activity and durability	[187]
Sm_0.25_Sr_0.25_Ca_0.25_La_0.25_MnO_3_	Thermochemical	—	Stable cycling	High CO yield	Reduced vacancy formation energy	[177]
Pr_0.1875_Ba_0.1875_Sr_0.1875_La_0.1875_Ca_0.25_CoO_3−*δ*_	SOFC	1.14 W cm^−2^	100 h	ORR	Fast oxygen exchange	[188]
Sr_2_(Fe_1.0_Ti_0.25_Cr_0.25_Mn_0.25_Mo_0.25_)O_6−*δ*_	SOEC	1.50 A cm^−2^ @ 1.5 V	160 h	Medium‐entropy perovskite	Increased oxygen vacancies	[189]
La(FeCuMnMgTi)O_3_	CO_2_RR	21.9 mA cm^−2^ @ −0.75 V	5 h	Heterogeneous catalyst	Synergistic electronic effects involve multiple cations and oxygen vacancies,	[190]
La(FeCoNiCrMn)O_3_	Photocatalysis	—	Stable	131.8 μmol g^−1 ^h^−1^ CO	Multimetal active sites	[178]
NiFe@Sr_2_Fe_0.4_V_0.4_Mo_0.4_Ni_0.4_Ti_0.6_O_6−*δ*_	CO_2_RR	1.66 A cm^−2^ @ 1.5 V	200 h	Reduced polarization	Faster electron transfer	[175]
Pr_1/6_La_1/6_Nd_1/6_Ba_1/6_Sr_1/6_Ca_1/6_FeO_3__−*δ*_	SOEC	1.47 A cm^−2^ @ 1.5 V	360 h	Enhanced electrochemical activity for sustainable reduction	Excellent long‐term stability with suppressed cation segregation	[191]
HE perovskite membrane	CO_2_ splitting	—	140 h	1.46 mL min^−1 ^cm^−2^ O_2_ flux	High oxygen permeability	[192]
La_0.2_Pr_0.2_Sm_0.2_Sr_0.2_Ca_0.2_FeO_2.9_‐δF_0.1_	SOEC	2.43 A cm^−2^ @ 1.6 V	300 h	F‐doped HEP	Suppressed cation migration, and excellent long‐term durability	[193]

### 5.2 Energy Conversion and Storage

#### 5.2.1 SOFCs

SOFCs are innovative electrochemical energy conversion devices that have attracted widespread interest owing to their high thermodynamic efficiency and fuel versatility [194]. Nevertheless, there are major obstacles that limit the future growth of this technology, such as the elemental segregation of traditional perovskite cathodes and an imbalance in thermal expansion between electrolytes and perovskites [195].

**FIGURE 12:**
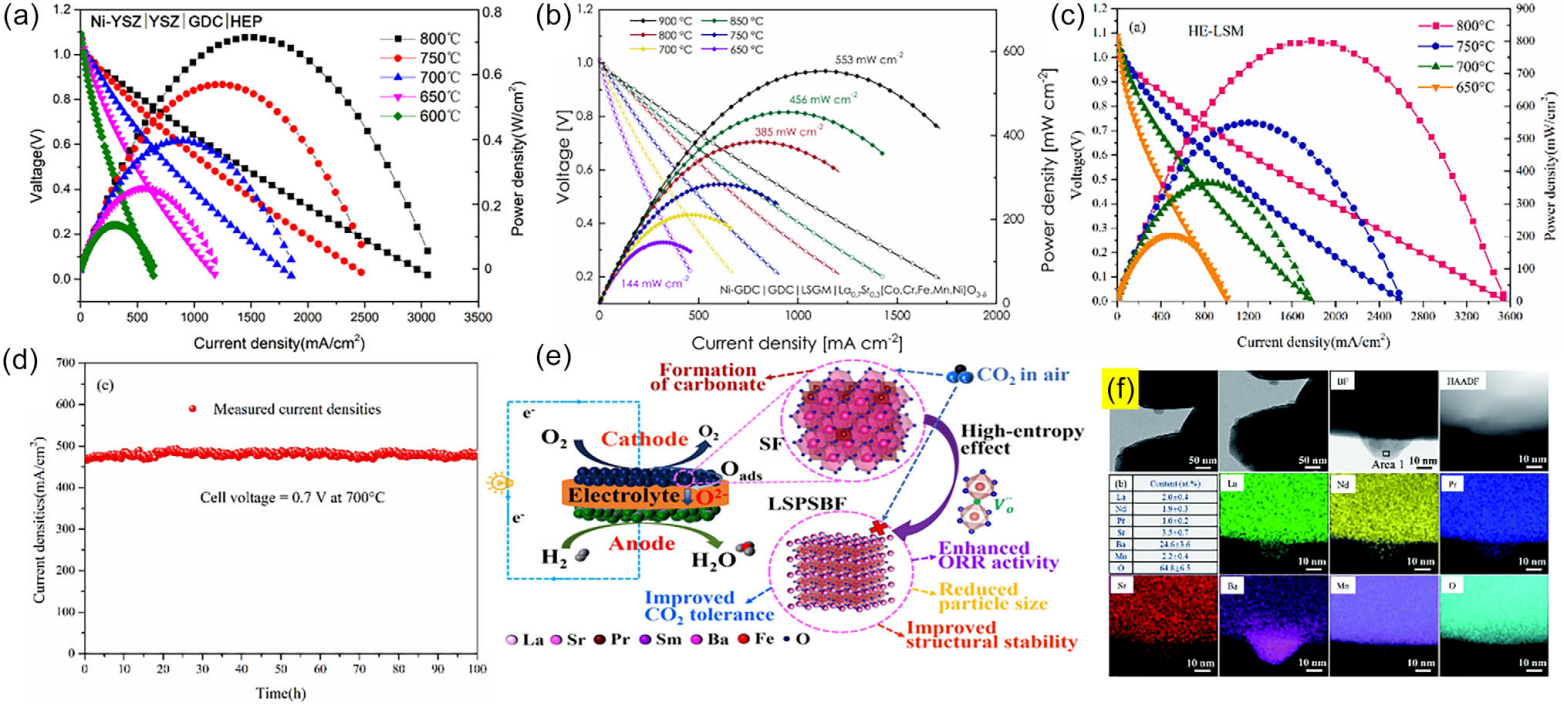
(a) Current–voltage and current–power curves of a single cell with an anode support and a HEP cathode were studied in a wet hydrogen environment at an intermediate temperature of 800°C–600°C. Reproduced with permission [27]. Copyright 2021, Elsevier. (b) *I*–*V*–*P* curves were measured at temperatures between 650°C and 900°C for Ni‐GDC|GDC|LSGM|La_0.7_Sr_0.3_(Co, Cr,Fe, Mn,Ni)O_3−*δ*_ button‐type fuel cell. Reproduced with permission [26]. Copyright 2020, Royal Society of Chemistry. (c) Typical *I*–*V–* *P* curves were measured at 650°C–800°C. (d) Stability study of NiO‐YSZ|YSZ|GDC|La_0.2_Pr_0.2_Nd_0.2_Sm_0.2_Sr_0.2_MnO_3−δ_ cell at 700°C with 0.7 V. Reproduced with permission [64]. Copyright 2021, Elsevier. (e) Diagrammatic illustration of the performance enhancement using (La_0.2_Sr_0.2_Pr_0.2_Sm_0.2_Ba_0.2_)FeO_3−*δ*_ based HEP. Reproduced with permission [196]. Copyright 2025, Elsevier. (f) TEM images and elemental distribution of (La_0.2_Nd_0.2_Pr_0.2_Sr_0.2_Ba_0.2_)MnO_3_ after annealing at 1000°C in air for 100 h. Reproduced with permission [197]. Copyright 2022, Royal Society of Chemistry.

Yang and colleagues constructed a 10‐cation based HEP La_0.2_Pr_0.2_Nd_0.2_Sm_0.2_Ba_0.1_Sr_0.1_Co_0.2_Fe_0.6_Ni_0.1_Cu_0.1_O_3−δ_ [27]. The HEP demonstrated a low polarization resistance (0.31 Ω cm^−2^) and excellent electrical conductivity (635.15 S cm^−1^) in air at 800°C. For comparison, the traditional perovskite La_0.8_Sr_0.2_FeO_3−δ_ has a polarization resistance of 0.60 Ω cm^2^ and an electrical conductivity of approximately 100 S cm^−1^ under the same environments. A button cell that was anode‐supported and based on YSZ demonstrated a remarkable peak power density of 714.5 mW cm^−2^ at 800°C (Figure 12a) [27].

The SOFC cathodes La_1−*x*_Sr_*x*_(Co, Cr,Fe, Mn,Ni)O_3−δ_ (*x* = 0, 0.1, 0.2, 0.3, 0.4, and 0.5) were constructed by Dabrowa et al. [26]. The synergistic interaction between different B‐site cations weakens the scaling‐relations limitations typically observed in conventional perovskites. The button cell with the anode supported by Ni‐Ce_0.8_Gd_0.2_O_2−*δ*_ and the cathode made of La_0.7_Sr_0.3_(Co, Cr,Fe, Mn,Ni)O_3−δ_ achieved a power density of 552 mW cm^−2^ at 900°C and 456 mW cm^−2^ at 850°C, respectively, as shown in Figure 12b. In addition, the thermal expansion coefficient of this HEP was lower (16.0 × 10^−6^ K^−1^) compared to the standard Co‐based Ba_0.5_Sr_0.5_Co_0.8_Fe_0.2_O_3−δ_ (20.3 × 10^−6^ K^−1^), which was caused by a reduced cobalt content [198]. Similarly, an entropy‐stabilized cathode perovskite based on (Pr_1/6_Nd_1/6_Sm_1/6_Ba_1/6_Sr_1/6_)_6/7_(Mn_1/6_Co)_6/7_O_3−*δ*_ achieved a PPD of around 1.41 W·cm^−2^ at 800°C, which is an 88% enhancement compared to traditional perovskite cathodes [199]. The study highlighted that dissociation, oxygen adsorption, and charge‐transfer kinetics are all improved by entropy‐induced lattice distortion. Strong metal–oxygen orbital interactions facilitate charge transport and improve electrode reaction kinetics.

The ORR activity and longevity of a perovskite oxide are significantly impaired by the deposition of A‐site elements that migrate toward the surface [200]. A‐site element segregation toward perovskite surfaces has been demonstrated to be influenced by low thermodynamic stability (Bonjae Koo2018 R). The La_0.2_Pr_0.2_Nd_0.2_Sm_0.2_Sr_0.2_MnO_3−δ_ SOFC cathode exhibits decreased Sr segregation, as demonstrated by Yang et al. [64], by the utilization of HEPs’ high thermodynamic stability. Figure 12c shows that at 750°C and 800°C, the constructed anode‐supported cell reached 549 and 801 mW cm^−2^, respectively, and Figure 12d shows that it remained stable at 700°C for 100 h. Electronic structure engineering through multication substitution effectively suppresses cation segregation during long‐term operation. The SEM results demonstrated that, although a conventional La_0.8_Sr_0.2_MnO_3−δ_ electrode showed numerous insulating white spots caused by Sr segregation on its surface following the stability test, the La_0.2_Pr_0.2_Nd_0.2_Sm_0.2_Sr_0.2_MnO_3−δ_ electrode remained unaffected by the same operating conditions [64]. Further enhancements have been realized through compositional engineering. Among several Fe‐based perovskites, a high‐entropy A‐site designed perovskite (Sr_0.2_Ba_0.2_Bi_0.2_La_0.2_Pr_0.2_)FeO_3_ demonstrated an exceptionally low area‐specific resistance (ASR) of 0.03 Ω·cm^2^ at 800°C [201]. The improved performance was ascribed to reduced energy for oxygen vacancy formation and the improved Fe valence modulation, which accelerated the adsorption and diffusion of oxygen. Similarly, iron‐based HEP (La_0.2_Sr_0.2_Pr_0.2_Sm_0.2_Ba_0.2_)FeO_3−*δ*_ is another important development [196]. The high‐entropy effect raises the number of oxygen vacancies, which improves ORR activity and stability in CO_2_‐containing atmospheres, which makes it crucial for real‐world SOFC applications (Figure 12e). Furthermore, Shi et al. reported that the SOFC cathode material (La_0.2_Nd_0.2_Sm_0.2_Ca_0.2_Sr_0.2_)MnO_3_ exhibited high electrical conductivity (≥200 S cm^−1^) in the temperature range of 750°C–1000°C and outstanding structural stability at high temperatures (1200°C–1400°C) [197]. Figure 12f shows that there was no discernible Sr segregation in the (La_0.2_Nd_0.2_Sm_0.2_Ca_0.2_Sr_0.2_)MnO_3_ that was annealed at 1000°C, according to the EDS data. The individual elements were uniformly distributed. To summarize, HEPs show exceptional promise as a stabilizing material for SOFC cathodes and as a means to inhibit A‐site element segregation. Operando monitoring should be used in future studies to quantitatively characterize elemental segregation. Table 3 contains a comprehensive list of reported HEPs‐based catalysts for SOFCs in recent years.

**TABLE 3:** Summary of some HEP‐based catalysis for SOFCs.

Material (HEP composition)	Electrolyte	Temperature, °C	**ASR, Ω cm** ^**2**^	**PPD, W cm** ^**−2**^	Key findings	Reference
La_0.2_Pr_0.2_Nd_0.2_Sm_0.2_Ba_0.1_Sr_0.1_Co_0.2_Fe_0.6_Ni_0.1_Cu_0.1_O_3−*δ*_	YSZ	800	0.30	0.7145	First HEP cathode, high conductivity (635 S/cm)	[27]
La_0.2_Pr_0.2_Nd_0.2_Sm_0.2_Sr_0⋅2_MnO_3−*δ*_	YSZ	800	0.40	0.80	Stable at the room temperature and stronger oxygen adsorption	[64]
(La_0.2_Pr_0.2_Nd_0.2_Sm_0.2_Gd_0.2_)_2_CuO_4_	GDC	700	0.52	0.528	First A2BO4‐type HEO cathode	[202]
La_1/6_Pr_1/6_Nd_1/6_Ba_1/6_Sr_1/6_Ca_1/6_FeO_3−*δ*_	SDC	750	—	1.439	72% Higher PPD vs. LSF	[203]
(Sr_0.2_Ba_0.2_Bi_0.2_La_0.2_Pr_0.2_)FeO_3_	SDC	800	0.03	0.66	Ultralow ASR via A‐site entropy	[201]
Pr_1/6_La_1/6_Sm_1/6_Ba_1/6_S_1/6_Ca_1/6_FeO_3−*δ*_	—	850	—	—	High oxygen vacancy & fast kinetics	[204]
Fe_0.6_Mn_0.6_Co_0.6_Ni_0.6_Cr_0.6_O_4_	BZCY	700	0.057	1.052	Low ASR + high stability	[205]
La_1−*x*_Sr_*x*_(Co, Cr,Fe, Mn,Ni)O_3−*δ*_ (*x* = 0, 0.1, 0.2, 0.3)	LSGM	900	0.126	0.55	Structural stability & compatibility	[26]
La_0.35_Pr_0.15_Sr_0.5_Fe_0.8_Ti_0.2_O_3−*δ*_	YSZ	800	0.17	0.99	Enhanced oxygen diffusion and surface reaction kinetics	[206]
Sr_0.75_Y_0.25_Co_0.9_Ru_0.1_O_3−*δ*_	YSZ	800	0.14	0.452	Enhanced thermal stabilit	[207]
GdBa(Fe_0.2_Mn_0.2_Co_0.2_Ni_0.2_Cu_0.2_)_2_O_5+*δ*_	YSZ	800	1.68	0.97	Excellent chemical compatibility	[208]
(La_0.2_Sr_0.2_Pr_0.2_Sm_0.2_Ba_0.2_)FeO_3−*δ*_	YSZ	800	0.058	1.084	Suppressed Sr segregation, high CO_2_ tolerance	[196]
Pr_0.2_La_0.2_Sm_0.2_Nd_0.2_Gd_0.2_BaCo_2_O_5+*δ*_	BZCYYb	700	0.064	1.01	High oxygen vacancy, fast kinetics	[209]
La_0.2_Pr_0.2_Eu_0.2_Ce_0.2_Sr_0.2_FeO_3−*δ*_	YSZ	700	0.136	0.451	Suppressed Sr segregation	[210]
SrFe_0.25_Ti_0.25_Co_0.25_Mn_0.25_O_3−*δ*_	LSGM	800	—	1.03	Enhance structural stability	[211]
Pr_1/6_La_1/6_Nd_1/6_Ba_1/6_Sr_1/6_Ca_1/6_CoO_3−*δ*_	Proton ceramic	600	—	1.21	Triple‐conducting electrode	[184]
PrBa_0.5_Sr_0.5_Co_1.5_Fe_0.5_O_5+*δ*_	GDC	800	0.3	1.0	Improves bulk oxygen diffusion	[212]
BaCo_0.4_Fe_0.4_Zr_0.1_Y_0.1_O_3−*δ*_	—	600	—	0.318	High proton conductivity (8.3 mS/cm)	[213]
La_0.7_Bi_0.1_Sr_0.2_Ni_0.2_Fe_0.8_O_3−*δ*_	YSZ	800	0.06	0.824	Fast oxygen reduction reaction	[214]
La_0.6_Sr_0.4_Co_0.8_Fe_0.2_O_3−*δ*_	YSZ	600	—	0.123	Enhanced ionic conductivity	[215]

Abbreviations: ASR‐ area‐specific resistance; BCZY‐ BaCe_0.7_Zr_0.1_Y_0.2_O_3−*δ*_; BZCYYb‐ BaZr_0.1_Ce_0.7_Y_0.1_Yb_0.1_O_3−*δ*__;_ GDC‐ Ce_0.9_Gd_0.1_O_1.95_; LSGM‐ La_0.8_Sr_0.2_Ga_0.8_Mg_0.2_O_3−*δ*__*;*_ PPD‐ peak power density; SDC‐ Sm_0.2_Ce_0.8_O_1.95_; YSZ‐ Yttria‐stabilized zirconia_._

#### 5.2.2 Solar Cells

HEPs have recently developed as a transformative materials platform for next‐generation solar cells, particularly due to their configurational disorder and entropy‐driven stabilization [216]. Unlike conventional perovskites that rely on a single or two cations, HEPs incorporate multiple elements at A‐, B‐, and X‐sites, leading to enhanced defect tolerance, reduced ion migration, and improved thermal and environmental stability. These advantages directly address the two major bottlenecks of PSCs: long‐term stability and reproducibility. Recent studies have demonstrated that entropy engineering can significantly reduce nonradiative recombination and stabilize crystal phases, resulting in superior photovoltaic performance compared to traditional single‐component perovskites.

**FIGURE 13:**
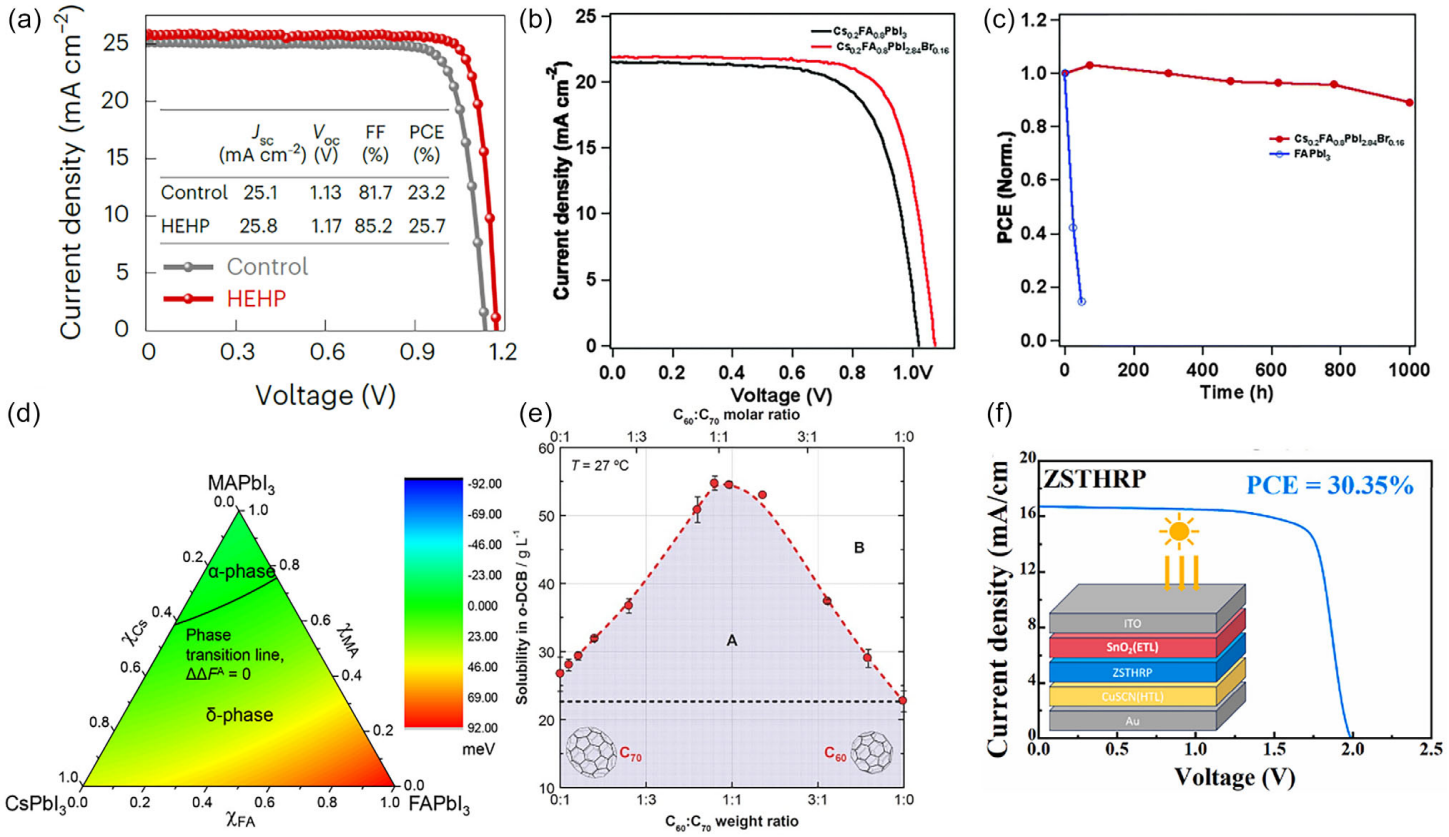
(a) *J*–*V* curves of the perovskite solar cells with and without high‐entropy halide perovskites. Reproduced with permission [217]. Copyright 2024, Springer Nature. (b) *J*–*V* curves for Cs_0.2_FA_0.8_PbI_3_ and Cs_0.2_FA_0.8_PbI_2.84_Br_0.16_ based PSCss. (c) Comparison of the stability of FAPbI_3_ and Cs_0.2_FA_0.8_PbI_2.84_Br_0.16_ PSCs (PCE remains ≈95% of the initial value after 1000 h) and FAPbI_3_ perovskite (PCE decays to ≈10% of the initial value in the first 50 h). Reproduced with permission [83]. Copyright 2016, Royal Society of Chemistry. (d) Schematic of the ternary phase illustrating the free energy difference between the trigonal and hexagonal phases. Reproduced with permission [218]. Copyright 2020, American Chemical Society. (e) Solubility of C60 and C70 in 1,2‐dichlorobenzene (o‐DCB) solvent at 27°C as a function of C60:C70 weight ratio (molar ratio). Reproduced with permission [219]. Copyright 2015, John Wiley and Sons. (f) PCE and construction of Cs_2_(ZrSnTeHfPt)Cl_6_‐based single‐junction solar cells device. Reproduced with permission [220]. Copyright 2025, Elsevier.

One of the most notable experimental breakthroughs was reported in a study on high‐entropy hybrid perovskites, where multiple organic cations were introduced at the A‐site to increase configurational entropy. This approach yielded a certified PCE of 25.5%–25.7%, comparable to that of conventional PSCs (Figure 13a) [217]. Importantly, the high‐entropy system exhibited enhanced resistance to thermal stress and phase segregation, which are common degradation pathways in mixed‐halide perovskites. The enhanced performance was ascribed to reduced lattice strain and suppressed defect formation due to the synergistic interaction of multiple cations [217]. In comparison, conventional mixed‐cation perovskites typically exhibit efficiencies in the range of 22%–24% with more pronounced instability under heat and moisture. The coexistence of diverse cations creates a broad distribution of local electronic environments, enabling efficient charge separation, rapid carrier transport, and suppression of recombination losses. Configurational entropy stabilizes these complex structures while inducing controlled lattice distortion, which tunes the band structure and facilitates charge extraction. Moreover, the interaction among multiple active sites promotes complementary pathways for light absorption and carrier migration, leading to enhanced photovoltaic efficiency and long‐term operational stability.

Yi and colleagues developed high‐performance metal halide perovskites that were stable in entropy by combining ions from the A and X sites [83]. The resultant Cs_0.2_FA_0.8_PbI_2.84_Br_0.16_ solar cell showed exceptional long‐term stability (over 1000 h; Figure 13c) and a high average PCE of 17.35% (Figure 13b) with little hysteresis. Saliba et al. produced an A‐site Rb‐substituted Cs_5/7_FA_1/7_MA_1/7_PbI_3_ perovskite that has better PCE and stability [221]. Subsequently, Senanayak et al. investigated the function of Rb substitution in Rb_0.05_Cs_0.05_FA_0.15_MA_0.75_PbI_3_ [222]. The researchers discovered that by substituting Rb, stability was improved and ion movement was decreased, leading to a suppression of hysteresis [221, 222] and provided good performance. Notably, compared to the standard benchmark material MAPbI_3_, Senanayak et al. found that Rb_0.05_Cs_0.05_FA_0.15_MA_0.75_PbI_3_ is less water adsorption sensitive using Brunauer–Emmett–Teller adsorption and thermogravimetric measurement. The 3D phase diagram, determined by ab initio thermodynamic calculations (Figure 13d), demonstrated by Kim et al. that substituting MA and Cs at the A‐site increases the conformational entropy of FAPbI_3_, making the material more stable in wet conditions [218]. Additional materials have also shown the stabilizing influence of configurational entropy. In their study, Mendaza and colleauges introduced the idea of “entropy dissolution,” which is seen in Figure 13e [219]. By combining C60 and C70, the solubility of fullerene, an acceptor material often utilized in organic polymer solar cells, was enhanced [223].

Further experimental validation of entropy‐driven enhancement was demonstrated in another study where HEP films were integrated into a standard n–i–p device architecture (ITO/SnO_2_/perovskite/Spiro‐OMeTAD/Ag). The high‐entropy device attained a PCE of 25.7%, outperforming the control device (23.2%). Additionally, the open‐circuit voltage (*V*_OC_) increased from 1.13 to 1.17 V, and the fill factor improved from 81.7% to 85.2%. More importantly, the device maintained more than 98% of its initial efficiency after 1000 h, demonstrating remarkable operational stability [224]. These results clearly indicate that entropy engineering not only enhances efficiency but also significantly improves device longevity, which is crucial for commercialization.

From a theoretical and computational perspective, HEPs also show great promise. A recent study proposed a systematic design strategy for multicomponent perovskites by identifying optimal elemental combinations at the B‐site. Using high‐throughput simulations, the authors designed five‐ and six‐element perovskites such as Cs_2_(ZrSnTeHfPt)Cl_6_, achieving simulated efficiencies of 17.67% and 30.35% (Figure 13f), respectively [220]. These results highlight the vast compositional space of HEPs and their potential to surpass the Shockley–Queisser limit in single‐junction devices through bandgap tuning and improved carrier transport. According to Zhang et al., these materials are more stable across different environments, but they still face a trade‐off between bandgap and efficiency, and their bandgaps are often much lower than the Shockley–Queisser limit [225]. Compared to conventional double perovskites, which often suffer from low absorption coefficients and poor charge mobility, high‐entropy systems provide a flexible platform for optimizing optoelectronic properties.

In addition to compositional engineering, entropy also plays a critical role in defect management. In conventional perovskites, defects such as vacancies and grain boundary traps lead to nonradiative recombination, limiting efficiency. However, in HEPs, the existence of multiple elements creates a complex energy landscape that can effectively “average out” defect states. This results in reduced trap density and improved charge carrier lifetimes. For example, studies on defect passivation in inorganic perovskites have shown efficiencies up to 15% with over 94% retention after long‐term storage, demonstrating that defect control directly correlates with improved stability [226]. While this example is not strictly high‐entropy, it supports the broader concept that defect suppression—enhanced further in HEPs‐is key to high‐performance PSCs. The aforementioned theoretical and experimental investigations have yielded compelling evidence for the conformational entropy‐driven enhancement of the material's stability, establishing a basis for the future advancement of solar cell materials, including HEPs. The performance of HEP as a catalyst for solar cells is presented in Table 4.

**TABLE 4:** Performance of HEP as catalysts for solar cells.

High‐entropy perovskite composition	Type	PCE, %	***V***_**OC**_ **, V**	**J**_**SC**_ **, mA cm^−2^**	FF, %	Key feature	Reference
Multication HE hybrid (FA/MA/Cs/Rb mixed)	Hybrid HEP	25.7	1.17	25.8	85.2	Record HE hybrid efficiency	[217]
Cs_2_{Zr_0.18_Sn_0.36_Te_0.27_Hf_0.09_Pt_0.1_}Cl_6_	Inorganic HE double	17.67	—	—	—	5‐Element B‐site tuning	[220]
Cs_0.2_FA_0.8_PbI_2.84_Br_0.16_	Organic–inorganic metal halide perovskites	17.35	1.073	21.9	74.2	Improved near‐IR solar light harvesting	[83]
Cs_2_{Zr_0.18_Sn_0.36_Te_0.27_Hf_0.09_Re_0.05_Pt_0.05_}Cl_6_	Inorganic HE double	30.35	—	—	—	6‐Element ultrahigh efficiency	[220]
(CdCuCoMnZn)S_*x*_	Quantum dot‐sensitized solar cells	8.33	0.665	25.60	49	Excellent mechanical, catalytic, and conductive performance	[227]
(Ti_0.2_Zr_0.2_Sn_0.2_Hf_0.2_Ce_0.2_)O_3_	pn junction	1.22	0.120	8.19 × 10–6	—	Improve kinetic equilibrium	[228]
Cs(Sn_62.5_Ge_25.0_Pb_12.5_)I_3_	Halide perovskites	33.4	0.91	41.79	87	Enhanced phase stability	[229]
CuGaO_2_/La(Ni_0.2_Fe_0.2_Co_0.2_Cr_0.2_Zn_0.2_)O_3_	pn junction	—	—	—	—	Good physical/mechanical stability	[230]

#### 5.2.3 Catalytic Energy Storage

The pursuit of sustainable energy solutions has significantly influenced the development of energy storage technologies. The absence of memory effect, increased efficiency, and long cycle life of lithium‐ion batteries (LIBs) have propelled them to the forefront of the solution spectrum [231]. Nevertheless, the demand for energy storage is on the rise, and the capabilities of conventional electrode materials are no longer sufficient. Consequently, innovative methods are required to improve energy density. The development of multication oxides is a possible approach to overcoming the constraints of existing electrode materials. HEPs are advanced catalytic energy‐storage materials that leverage multication disorder to enhance oxygen‐vacancy formation, electronic conductivity, and redox‐active sites. This entropy‐stabilized mixing results in improved capacitive storage, battery kinetics, and catalytic charge transfer, enabling HEPs to outperform traditional binary and ternary perovskites across various energy storage applications, including dielectric capacitors and supercapacitors.

**FIGURE 14:**
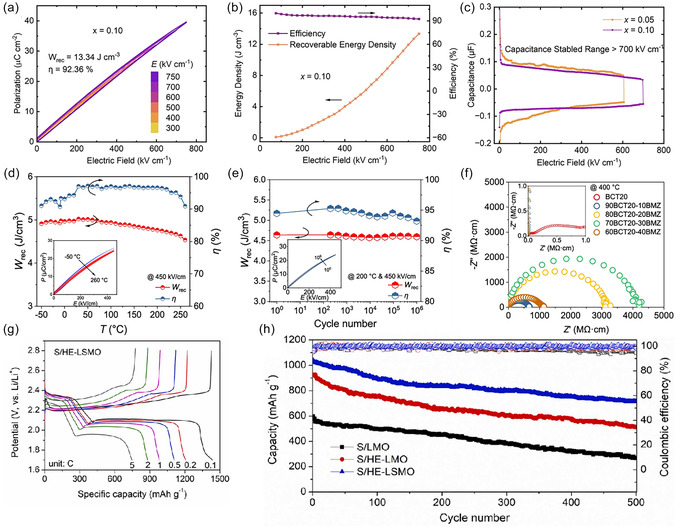
(a) Field‐dependent polarization‐electric field (P–E) loop for 0.10NaTaO_3_−0.15SrTiO_3_–*x*Ca(Hf_*x*/3_Zr_*x*/3_Sn_*x*/3_)O_3_ (*x* = 0.10). (b) Field‐dependent energy storage performance of 0.10NaTaO_3_−0.15SrTiO_3_–*x*Ca(Hf_*x*/3_Zr_*x*/3_Sn_*x*/3_)O_3_ (*x* = 0.10). (c) Evaluation of field‐dependent capacitance of capacitors made with 0.10NaTaO_3_−0.15SrTiO_3_–*x*Ca(Hf_*x*/3_Zr_*x*/3_Sn_*x*/3_)O_3_ (*x*  = 0.05 and 0.10) in the same geometry. Reproduced with permission [232]. Copyright 2025, Springer Nature. (d) Temperature‐dependent *W*_rec_ and *η* values. The P–E loops at various temperatures are displayed in the inset. (e) Cycling stability was tested at 450 kV/cm and 200°C for up to 10^6^ cycles. The P–E loops following different cycles are displayed in the inset. (f) Electrochemical impedance plots of (1−*x*)(Ba_0.8_Ca_0.2_)TiO_3_–*x*Bi(Mg_0.5_Zr_0.5_)O_3_ ceramics at 400°C. Reproduced with permission [233]. Copyright 2025, Springer Nature. (g) Charge–discharge profiles of prepared HEP‐based electrodes and (h) cycle stability at 1 C of sulfur‐loaded HEP composites. Reproduced with permission [234]. Copyright 2023, Elsevier.

For example, Fan et al. described a high‐entropy quantum‐paraelectric perovskite exhibiting field‐independent polarization and ultrahigh recoverable energy density in dielectric capacitors [232]. The findings indicate a high recoverable energy density (*W*_rec_) of 13.3 J cm^−3^ with an efficiency of 92.4% and a breakdown strength of 750 kV/cm achieved in the high‐entropy modulated quantum paraelectric perovskite (0.75−*x*)NaNbO_3_−0.10NaTaO_3_−0.15SrTiO_3_–*x*Ca(Hf_*x*/3_Zr_*x*/3_Sn_*x*/3_)O_3_ (*x* = 0.10) in bulk form (Figure 14a–c). The entropy‐modulated lattice suppressed long‐range ferroelectric ordering and stabilized reversible polarization switching, thereby yielding superior charge–discharge efficiency across a wide range of electric fields. Similarly, Duan and colleagues developed high‐entropy superparaelectric perovskites with diverse ferroic distortions [235], achieving a remarkable *W*_rec_ of 15.48 J cm^−3^ and an energy storage efficiency (η) of 90.02% at a breakdown strength (Eb) of 710 kV cm^−1^ in the composition (1 −*x*) Bi_0.47_Na_0.47_Ba_0.06_TiO_3_–*x* Sr_0.7_La_0.2_Ta_0.2_Ti_0.75_O_3_ (*x* = 0.30). Ning et al. achieved a *W*_rec_ of 5.8 J cm^−3^ at a breakdown strength of 320 kV cm^−1^ in flash‐sintered (Na_0.2_Bi_0.2_Ba_0.2_Sr_0.2_Ca_0.2_)TiO_3_ high‐entropy ferroelectric perovskite ceramics [236]. The enhancement was attributed to entropy‐stabilized grain boundaries and reduced leakage current, which improved the dielectric breakdown strength and the reversible polarization behavior. More importantly, Kong et al. developed high‐entropy BaTiO_3_‐based perovskites with a *W*_rec_ of 10.9 J cm^−3^ and 93% efficiency under 720 kV cm^−1^, significantly outperforming conventional relaxor perovskites (Figure 14d–f) [233]. The entropy‐stabilized polar nanoregions enhanced breakdown strength and enabled rapid charge storage/release behavior. Additionally, the material maintained stable energy storage from −50°C to 260°C with negligible performance degradation, demonstrating exceptional stability for catalytic energy‐storage devices.

A study by Zhang and his research group highlighted HEPO nanofibers, specifically La_0.8_Sr_0.2_(CrMnFeCoNi)O_3_, used in lithium‐sulfur batteries as effective bidirectional catalytic hosts for converting polysulfides [234]. This electrode achieved a remarkable areal capacity of 6.6 mAh  cm^−2^ with a sulfur loading of 8.4 mg cm^−2^ (Figure 14g–h). The nanofibers facilitated rapid Li_2_S nucleation and polysulfide conversion through multiple catalytic sites. The entropy‐stabilized lattice structure contributed to improved adsorption and redox kinetics, making it superior to conventional single‐metal perovskites, which generally have slower Li_2_S decomposition rates. In contrast, a HEP metal‐fluoride (K_0.9_(Mg_0.2_Mn_0.2_Co_0.2_Ni_0.2_Cu_0.2_)F_2.9_) anode synthesized via a one‐pot method exhibited 698 mAh g^−1^ at 0.1 A g^−1^ and 311 mAh g^−1^ at 3.2 A g^−1^, with improved rate performance and long‐term activation behavior [237]. The authors attributed the enhancement to entropy‐induced defect density and improved Li^+^ diffusion channels. Compared with conventional perovskite fluorides (<400 mAh g^−1^), the high‐entropy system showed significantly higher capacity and rate capability, confirming that compositional disorder promotes pseudocapacitive lithium storage. Similarly, another HEP fluoride electrode from recycled batteries showed impressive lithium storage capability due to a multication framework (K–Li–Mg–Mn–Co–Ni–Cu) that stabilizes conversion reactions and enhances cycling durability [238]. Yan et al. constructed the HEP oxide [(Bi, Na)_0.2_(La, Li)_0.2_(Ce, K)_0.2_Ca_0.2_Sr_0.2_]TiO_3_, which has a capacity of 120.4 mA h g^−1^ after 300 cycles at 1000 mA g^−1^. For supercapacitors, porous HEP nanoparticles synthesized via a MOF‐gel route exhibited a specific surface area of 43.2 m^2 ^g^−1^ and enabled a solid‐state device with an energy density of 31.2 Wh kg^−1^ and a power density of 14 500 W kg^−1^ [239]. The results show that HEPs can improve the performance of different batteries and supercapacitors by addressing key challenges and enabling their use in more energy storage applications in the future.

### 5.3 Degradation of Organic Pollutants

A major environmental concern is the increasing release of organic pollutants into water sources, which is driven by both world population expansion and industrialization [240, 241, 242]. Novel strategies are required, as conventional wastewater treatment processes are unable to mineralize these complex chemicals [243]. A new and effective method for treating wastewater is heterogeneous photocatalysis, which is an aspect of advanced oxidation processes (AOPs). This method employs highly reactive oxygen‐based radicals to decompose organic contaminants into harmless substances. However, compared to traditional transition‐metal catalysts, it has significant drawbacks in terms of accessibility, affordability, and toxicity.

**FIGURE 15:**
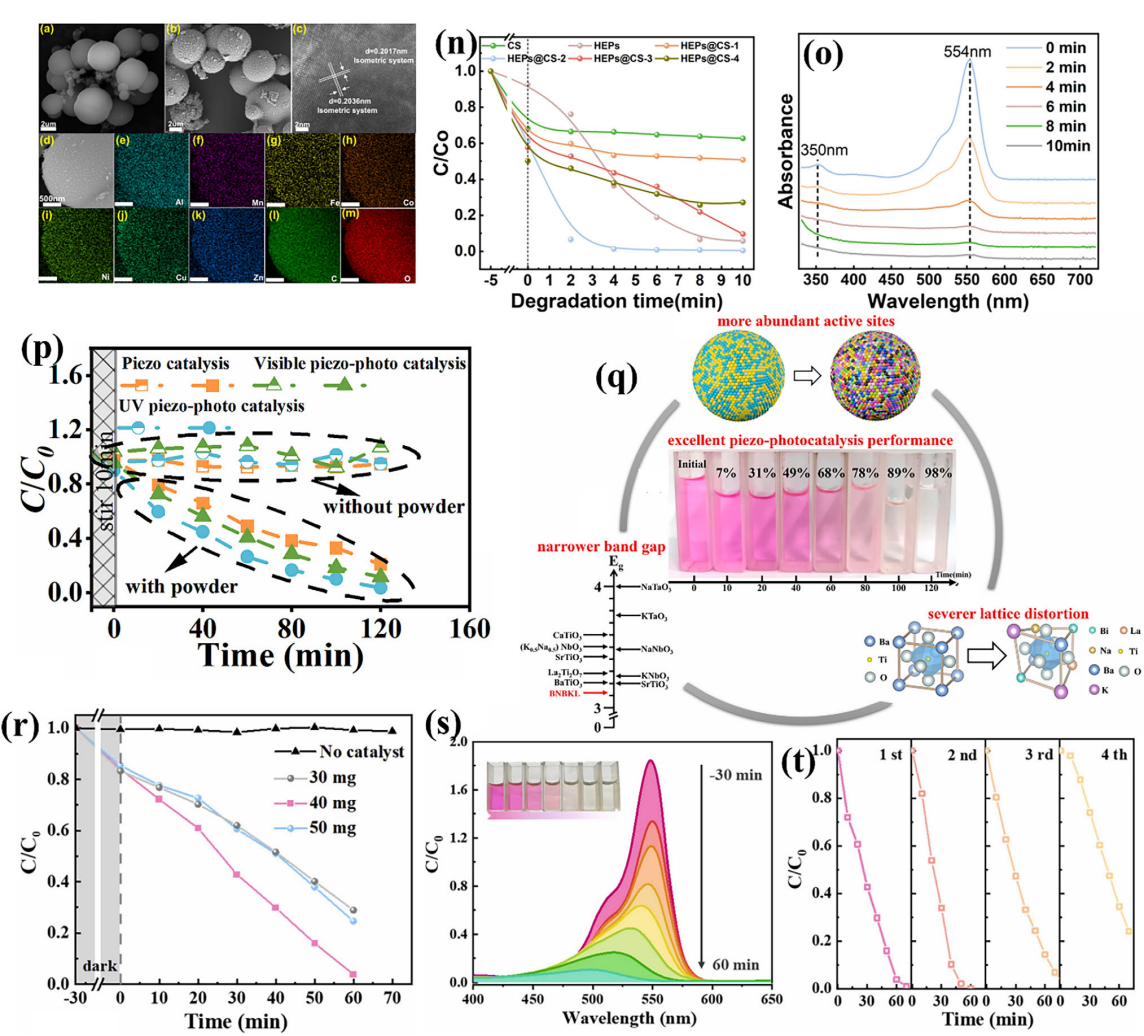
(a–c) HRTEM image, (d–m) EDS mappings of La(Al_1/7_Mn_1/7_Fe_1/7_Co_1/7_Ni_1/7_Cu_1/7_Zn_1/7_)O_3_@ carbon microsphere, (n) RhB decomposition via various catalysts in a Fenton‐like mechanism, and (o) UV–vis absorption spectra for varying RhB degradation intervals. Reproduced with permission [244]. Copyright 2025, Elsevier. (p) The piezo– and piezo–photo catalytic performance of the degradation of RhB using Bi_0.2_Na_0.2_Ba_0.2_K_0.2_La_0.2_)TiO_3_ HEP catalysts as a function of time. (q) Schematic illustration of the advantages of Bi_0.2_Na_0.2_Ba_0.2_K_0.2_La_0.2_)TiO_3_ HEP. Reproduced with permission [245]. Copyright 2024, Elsevier. (r) The rate of RhB degradation with varying amounts of catalyst, (s) UV–vis spectra of RhB in the existence of Cs(Mn_0.2_Ni_0.2_Sn_0.2_Co_0.2_Bi_0.1_)Br_3_, and (t) Cs(Mn_0.2_Ni_0.2_Sn_0.2_Co_0.2_Bi_0.1_)Br_3_ was tested using a sequential deterioration cycle. Reproduced with permission [246]. Copyright 2025, Elsevier.

In this context, HEPs have recently emerged as highly efficient catalysts for decomposition of organic contaminants owing to their multiple redox‐active metal centers, tunable electronic structures and abundant oxygen vacancies. A recent study reported a HEP embedded in carbon (La(Al_1/7_Mn_1/7_Fe_1/7_Co_1/7_Ni_1/7_Cu_1/7_Zn_1/7_)O_3_@carbon microspheres) for peroxymonosulfate (PMS) activation, where rapid Rhodamine B degradation was achieved over a wide pH range with excellent recyclability [244]. The narrowed band structure and modified electronic states of HEPs enhance visible‐light absorption and charge separation efficiency. The introduction of multiple cations creates intermediate electronic states that facilitate the migration of photogenerated carriers. In Figure 15a–m, TEM and EDS mapping confirmed a uniform distribution of critical elements (Co, Zn, Al, Cu, Fe, Mn, and Ni), resulting in lattice distortion and increased entropy in the material. The significant achievement was a 99.7% degradation efficiency for RhB within 10 min (Figure 15n,o), with a rate constant of 0.5815 min^−1^. The multimetal synergy promoted nonradical and radical pathways, improving electron transfer and pollutant adsorption compared with single‐component perovskites. The catalyst maintained high stability after several cycles, demonstrating the advantage of entropy‐stabilized lattice distortion and numerous active sites [244].

Fu and colleagues developed a novel composite material (Bi_0.2_Na_0.2_Ba_0.2_K_0.2_La_0.2_)TiO_3_ (BNBKL) for the effective decomposition of contaminants like rhodamine B (RhB) through a combination of ball milling and calcination techniques [245]. The degradation rates for RhB were quantified at 7.65 × 10^−3 ^min^−1^ under visible light (Vis) and 6.85 × 10^−3 ^min^−1^ under ultraviolet (UV) light. Notably, under piezoelectric‐photocatalytic conditions, these rates increased to 19.05 × 10^−3 ^min^−1^ (Vis) and 24.26 × 10^−3 ^min^−1^ (UV), demonstrating a significant synergistic effect beyond merely additive rates of photocatalytic or piezoelectric degradation. BNBKL achieved an impressive 98% degradation of RhB within 120 min (Figure 15p,q) [245]. Furthermore, the material exhibited varying efficiencies for different dyes, achieving 98% for methyl orange (MO), 73% for methylene blue (MB), and 46% for amido black 10B (AB10B). These findings highlight the superior photocatalytic capabilities of BNBKL, attributed to the incorporation of diverse metal ions that enhance active site availability, induce lattice distortion, and improve light absorption, collectively leading to enhanced piezoelectric photodegradation performance.

Similarly, Sharma et al. developed HEP ceramic (Bi_0.2_K_0.2_Na_0.2_Ba_0.2_Sr_0.2_)TiO_3_, which exhibited a photocatalytic degradation rate of MB that is nearly 7.4 times greater than that of conventional BaTiO_3_ [247]. This improvement is accredited to the occurrence of Ti^3+^ species, oxygen vacancies, and lattice distortions from multicomponent cations, which improve bandgap alignment, enhancing visible‐light absorption and accelerating photogenerated charge separation, ultimately leading to more efficient dye mineralization. The findings indicate that configurational entropy can enhance photocatalytic kinetics relative to single‐composition perovskites. Another HEP ceramic (Bi_0.2_K_0.2_Na_0.2_Ca_0.2_Ba_0.2_)TiO_3_ demonstrated excellent dye degradation under both indoor light and sunlight irradiation [248]. The multicomponent A‐site substitution induced bandgap narrowing and increased surface defect density, leading to faster photocatalytic decomposition of organic dyes compared with single‐component perovskites. The study showed that high‐entropy engineering improves visible‐light absorption and enhances hydroxyl radical generation, thereby accelerating degradation efficiency. Furthermore, high‐entropy lead‐free halide perovskite nanoparticles Cs(Mn_0.2_Ni_0.2_Sn_0.2_Co_0.2_Bi_0.1_)Br_3_ showed remarkable photocatalytic degradation of Rhodamine B (Figure 15r–t) [246]. The catalyst degraded more than 97% of 10 mg L^−1^ RhB within 60 min under visible light. The multicomponent active sites and defect‐rich structures of HEPs can facilitate the activation of oxidizing species while maintaining structural stability under harsh wastewater treatment conditions. Such findings suggest that entropy engineering could be an effective strategy for developing durable catalysts for the remediation of emerging contaminants [249]. The wide absorption band and multi‐ion doping enhanced electron–hole separation and reduced recombination compared with conventional Mn‐based perovskites. Comparative analysis of HEP‐based photocatalysts for the decomposition of organic contaminants is presented in Table 5.

**TABLE 5:** Compared with HEP‐based photocatalysts for the degradation of organic contaminants.

High‐entropy perovskite	Pollutant	Pollutant concentration, mg/L	Catalyst dose, mg	Degradation performance	**Rate constant, min** ^**−1**^	Light source	Reference
(Bi_0.2_K_0.2_Na_0.2_Ba_0.2_Sr_0.2_)TiO_3_	Methylene Blue	7.5	100 g	61% in 180 min	5.57 × 10^−3^	UV lamp, 3 × 8 W	[247]
(Bi_0.2_K_0.2_Na_0.2_Ca_0.2_Ba_0.2_)TiO_3_	Methylene Blue	7.5	100	63% in 180 min	4.7 × 10^−3^	UV light (360 nm, 3 × 8 W)	[248]
(Bi_0.2_K_0.2_Na_0.2_Ba_0.2_Ca_0.2_)TiO_3_	Methylene Blue	7.5	200	84% in 180 min	9.9 × 10^−3^	UV light (3 × 8 W, 360 nm)	[250]
(Bi0_0.2_K_0.2_Na_0.2_Ba_0.2_Ca_0.2_)TiO_3_	Methylene Blue	7.5	200	92% in 180 min	14 × 10^−3^	Sunlight	[250]
Pb(Mg_1/3_Nb_2/3_)O_3_‐_0.3_PbZrO_3–0.45_Pb(Ti_*x*_Hf_1−*x*_)O_3_	Methylene Blue	—	—	95% in 60 min	∼0.05	Piezocatalysis	[251]
(La(Al_1/7_Mn_1/7_Fe_1/7_Co_1/7_Ni_1/7_Cu_1/7_Zn_1/7_)O_3_@carbon microspheres	Rhodamine B	50	4	99.7% in 10 min	0.5815	PMS activation	[244]
Cs(Mn_0.2_Ni_0.2_Sn_0.2_Co_0.2_Bi_0.1_)Br_3_	Rhodamine B	10	40	97% in 60 min	43.58	300 W xenon lamp	[246]
(Bi_0⋅2_Na_0⋅2_Ba_0⋅2_K_0⋅2_La_0.2_)TiO_3_	Rhodamine B	5	50	96% in 120 min	24.26 × 10^−3^	300 W Xe lamp	[245]
(Bi_0⋅2_Na_0⋅2_Ba_0⋅2_K_0⋅2_La_0.2_)TiO_3_	Amido black 10 B	5	50	98% in 120 min	39.18 × 10^−3^	300 W Xe lamp	[245]
(Bi_0⋅2_Na_0⋅2_Ba_0⋅2_K_0⋅2_La_0.2_)TiO_3_	Methylene blue	5	50	73% in 120 min	10.75 × 10^−3^	300 W Xe lamp	[245]
(Bi_0⋅2_Na_0⋅2_Ba_0⋅2_K_0⋅2_La_0.2_)TiO_3_	Methyl orange	5	50	46% in 120 min	5.12 × 10^−3^	300 W Xe lamp	[245]
0.25Pb(Mg_1/3_Nb_2/3_)O_3_ −0.75Pb(Hf_0.2_Zr_0.42_Ti_0.38_)O_3_	Methylene blue	10	45	90% in 60 min	0.01948	150 W xenon lamp	[252]
Sr_32_Ti_8_Sn_8_Nb_4_Ta_4_Fe_8_O_96_	Cr (VI)	5	5	93% in 40 min	0.0186	UV 75 W	[39]
0.90BaTiO_3_−0.10Sm(Ca_0.2_Zn_0.2_Zr_0.2_Sn_0.2_Hf_0.2_)O_3_	Rhodamine B	5	100	91% in 60 min	38.7 × 10^−3^	100 W piezo‐catalytic technology	[253]
0.90BaTiO_3_−0.10Sm(Ca_0.2_Zn_0.2_Zr_0.2_Sn_0.2_Hf_0.2_)O_3_	Methylene blue	5	100	86% in 60 min	31.8 × 10^−3^	100 W piezo‐catalytic technology	[253]
0.90BaTiO_3_−0.10Sm(Ca_0.2_Zn_0.2_Zr_0.2_Sn_0.2_Hf_0.2_)O_3_	Methyl orange	5	100	69% in 60 min	19.4 × 10^−3^	100 W piezo‐catalytic technology	[253]

## 6 Conclusion and Future Perspectives

HEPs have recently emerged as a versatile and rapidly expanding class of materials that extend beyond conventional oxide‐based systems to include halides, fluorides, oxynitrides, chalcogenides, and hybrid perovskites. The incorporation of numerous cations into the A and/or B sublattices results in a highly disordered, thermodynamically stabilized lattice with severe lattice distortion, multimetallic interactions, and highly tunable electronic structures in HEPs. These features collectively enable a continuous distribution of adsorption energies, improved defect chemistry, and enhanced structural robustness under harsh operating conditions. As discussed in this review, entropy engineering has enabled the rational development of HEP oxides, fluorides, halide perovskites, and mixed‐anion systems for a broad spectrum of applications, including oxygen evolution and reduction reactions, hydrogen evolution, ammonia decomposition, CO_2_ reduction, photocatalytic processes, and pollutant degradation. Furthermore, the ability to tailor both cationic and anionic disorder significantly expands the compositional landscape, offering unprecedented flexibility in optimizing catalytic activity, conductivity, and durability.

Significant progress has also been achieved in the development of HEP synthesis techniques. Conventional solid‐state and sol–gel methods remain widely used, while advanced approaches, including SPS, flash sintering, NSP, hydrothermal, sonochemical, and UHS, offer new opportunities for lowering synthesis temperatures, controlling particle size, and improving compositional homogeneity. Meanwhile, computational tools, including DFT, SQSs, CALPHAD, and machine‐learning‐assisted descriptors, are beginning to unravel the thermodynamic stabilization mechanisms and catalytic behavior of these highly disordered systems. The combination of experimental and computational insights is gradually enabling predictive design of HEPs, although this field is still at an early stage.

Despite the rapid development, several fundamental challenges remain. To begin with, the interactions between configurational entropy, local ordering, anion disorder and catalytic performance are not well comprehended, especially for nonoxide HEPs such as fluorides and halides. Second, the majority of reported HEPs are based on equimolar compositions, with the extensive nonequimolar compositional space remaining mostly unexplored. Third, phase purity and homogeneity across chemistries with different anions are difficult to control, particularly in hybrid and low‐temperature syntheses. Moreover, multicomponent environments and dynamic surface reconstruction by operating conditions complicate mechanistic knowledge of active sites. These challenges highlight the need for advanced in situ/operando characterization methods and multiscale modeling to elucidate structure–property relationships in complex HEP systems.

The future study of HEPs will likely shift to several promising directions. (i) The further development of compositional design with multianion HEPs (oxide‐fluoride, oxide‐nitride, halide‐based systems) might offer new avenues of electronic structure and catalytic pathways control. (ii) Nonequimolar and gradient entropy designs have the potential to unlock better catalytic activities in comparison to the traditional equiatomic compositions. (iii) Integrating high‐throughput computation and machine learning with experimental synthesis will accelerate discovery across the enormous compositional space of HEPs. (iv) Scalable and low‐temperature synthesis methods will be essential to practical uses, especially of hybrid and halide HEPs. (v) Constructing heterostructures, nanostructured architectures, and hybrid composites with conductive supports may significantly improve charge transfer and catalytic durability. Lastly, the ability of entropy‐stabilized perovskites to have a broader range of applications in catalysis, energy storage, thermoelectrics, dielectric materials and optoelectronic devices will further highlight the versatile nature of these materials.

In summary, HEPs represent a new paradigm in catalyst design, where compositional disorder is deliberately exploited to create multifunctional active sites and robust structures. Further progress in synthesis, theoretical insight, and application‐oriented design will enable the next generation of HEPs, including oxides, fluorides, halides, and hybrid materials, to support sustainable energy and environmental technologies.

## Author Contributions

**K. Aravinthkumar:** conceptualization, validation, data curation, investigation, resources, writing – original draft. **Pei‐Ying Lin:** conceptualization, formal analysis, data curation. **Shu‐Ling Hsieh:** writing – review & editing, supervision, formal analysis, resources. **Shuchen Hsieh:** writing – review & editing, visualization, validation, supervision, investigation, formal analysis, data curation.

## Funding

This study was supported by National Science and Technology Council (Grants NSTC 114‐2221‐E‐992‐019‐MY3, 114‐2113‐M‐110‐006, and 114‐2811‐M‐110‐023).

## Conflicts of Interest

The authors declare no conflicts of interest.

## Supporting information

Supplementary Material

## Data Availability

The data that support the findings of this study are available from the corresponding author upon reasonable request.
