# Supplementary material for: Beyond Single‐Active Sites: The Emergence of High‐Entropy Perovskites in Energy and Environment Catalysis
Source: ChemSusChem. 2026 Jul 2;19(13):e70851. doi: 10.1002/cssc.70851 (PMC13327456; doi:10.1002/cssc.70851)
Supplement: Supplementary file 1 — Supplementary Material [file CSSC-19-e70851-s001.pdf]

Supporting information

Beyond Single-Active Sites: The Emergence of High-Entropy Perovskites in Energy and Environment Catalysis

K. Aravinthkumar [1, 2], Pei-Ying Lin [1, 2], Shu-Ling Hsieh [1], Shuchen Hsieh * [2, 3, 4, 5, 6]

[1] Department of Seafood Science, National Kaohsiung University of Science and Technology, Kaohsiung 81157, Taiwan.

[2] Department of Chemistry, National Sun Yat-sen University, No. 70, Lien-Hai Road, Gushan District, Kaohsiung City 80424, Taiwan.

[3] Center for Green-Energy Key Materials Research, National Sun Yat-Sen University, Kaohsiung 80424, Taiwan.

[4] School of Pharmacy, College of Pharmacy, Kaohsiung Medical University, 100 Shih-Chuan 1st Rd., Kaohsiung 80708, Taiwan.

[5] Regenerative Medicine and Cell Therapy Research Center, Kaohsiung Medical University, 100 Shih-Chuan 1st Rd., Kaohsiung 80708, Taiwan.

[6] Institute of Aquatic Science and Technology, College of Hydrosphere Science, National Kaohsiung University of Science and Technology 142, Haijhuan Rd., Kaohsiung 81157, Taiwan.

<u>Contact correspondence to</u>

Prof. Shuchen Hsieh; E-mail: shsieh@faculty.nsysu.edu.tw

Department of Chemistry, National Sun Yat-sen University, Taiwan.

Table S1: Compositions, ion-substitution sites, and synthesis techniques of HEPs documented in the literature

Composition	Number of A-site elements	Number of B-site elements	Reaction temperature (°C)	Synthesis method	References
$Ba(Zr_{0.2}Ti_{0.2}Sn_{0.2}Hf_{0.2}Nb_{0.2})O_3$	1	5	1350	SSR	[1]
$Ba(Zr_{0.2}Ti_{0.2}Sn_{0.2}Hf_{0.2}Mo_{0.2})O_3$	1	5	1350	SSR	[1]
$Ba(Zr_{0.2}Ti_{0.2}Sn_{0.2}Hf_{0.2}W_{0.2})O_3$	1	5	1350	SSR	[1]
$Ba(Zr_{0.2}Ti_{0.2}Sn_{0.2}Hf_{0.2}V_{0.2})O_3$	1	5	1350	SSR	[1]
$Ba(Zr_{0.2}Ti_{0.2}Sn_{0.2}Hf_{0.2}Ta_{0.2})O_3$	1	5	1350	SSR	[1]
$Ba(Ti_{1/6}Sn_{1/6}Zr_{1/6}Hf_{1/6}Nb_{1/6}Ga_{1/6})O_3$	1	6	1600	SSR	[2]
$(Na_{0.2}Bi_{0.2}Ba_{0.2}Sr_{0.2}Ca_{0.2})TiO_3$	5	1	1220	SSR	[3]
$Sr(Zr_{0.2}Sn_{0.2}Ti_{0.2}Hf_{0.2}Mn_{0.2})O_3$	1	5	1300-1500	SSR	[4]
$Sr(Zr_{0.2}Sn_{0.2}Ti_{0.2}Hf_{0.2}Nb_{0.2})O_3$	1	5	1300-1500	SSR	[4]
$Sr(Zr_{0.2}Sn_{0.2}Ti_{0.2}Hf_{0.2}Y_{0.2})O_3$	1	5	1300-1500	SSR	[4]
$Sr(Zr_{0.2}Sn_{0.2}Ti_{0.2}Hf_{0.2}Ce_{0.2})O_3$	1	5	1300-1500	SSR	[4]
$Sr(Zr_{0.2}Sn_{0.2}Ti_{0.2}Hf_{0.2}Ge_{0.2})O_3$	1	5	1300-1500	SSR	[4]
$Ba(Zr_{0.2}Sn_{0.2}Ti_{0.2}Hf_{0.2}Mn_{0.2})O_3$	1	5	1300-1500	SSR	[4]
$Ba(Zr_{0.2}Sn_{0.2}Ti_{0.2}Hf_{0.2}Nb_{0.2})O_3$	1	5	1300-1500	SSR	[4]
$Ba(Zr_{0.2}Sn_{0.2}Ti_{0.2}Hf_{0.2}Y_{0.2})O_3$	1	5	1300-1500	SSR	[4]
$Ba(Zr_{0.2}Sn_{0.2}Ti_{0.2}Hf_{0.2}Ce_{0.2})O_3$	1	5	1300-	SSR	[4]

			1500		
$Ba(Zr_{0.2}Sn_{0.2}Ti_{0.2}Hf_{0.2}Ge_{0.2})O_3$	1	5	1300-1500	SSR	[4]
$(Sr_{0.5}Ba_{0.5})(Zr_{0.2}Sn_{0.2}Ti_{0.2}Hf_{0.2}Nb_{0.2})O_3$	2	5	1300-1500	SSR	[4]
$(Na_{0.30}K_{0.07}Ca_{0.2}4La_{0.18}Ce_{0.21})TiO_3$	5	1	1400	SSR	[5]
$La(Cr_{0.2}Mn_{0.2}Fe_{0.2}Co_{0.2}Ni_{0.2})O_3$	1	5	1400	SSR	[6]
$[(Bi,Na)_{0.2}(La,Li)_{0.2}(Ce,K)_{0.2}Ca_{0.2}Sr_{0.2}]TiO_3$	8	1	1250	SSR	[7]
$Ba(Zr_{0.2}Sn_{0.2}Ti_{0.2}Hf_{0.2}Nb_{0.2})O_3$	1	5	1400	SSR	[8]
$(Ba_{0.2}Sr_{0.2}Ca_{0.2}Bi_{0.2}La_{0.2})TiO_3$	5	1	1350	SSR	[9]
$(Ba_{0.2}Sr_{0.2}Ca_{0.2}Bi_{0.2}Na_{0.2})TiO_3$	5	1	1350	SSR	[9]
$(Ba_{0.2}Sr_{0.2}Ca_{0.2}La_{0.2}Na_{0.2})TiO_3$	5	1	1350	SSR	[9]
$(Bi_{0.2}K_{0.2}Ba_{0.2}Sr_{0.2}Ca_{0.2})TiO_3$	5	1	1175	SSR	[10]
$Sr(Ti_{0.2}Fe_{0.2}Mo_{0.2}Nb_{0.2}Cr_{0.2})O_3$	1	5	1227	SSR	[11]
$BaZr_{0.2}Sn_{0.2}Ti_{0.2}Hf_{0.2}Ce_{0.2}O_{3-\delta}$	1	5	1400	SSR	[12]
$BaZr_{0.2}Sn_{0.2}Ti_{0.2}Hf_{0.2}$-$Y_{0.2}O_{3-\delta}$	1	5	1400	SSR	[12]
$BaZr_{1/7}Sn_{1/7}Ti_{1/7}Hf_{1/7}Ce_{1/7}Nb_{1/7}Y_{1/7}O_{3-\delta}$	1	7	1400	SSR	[12]
$BaZr_{0.15}Sn_{0.15}Ti_{0.15}Hf_{0.15}Ce_{0.15}Nb_{0.15}Y_{0.10}O_{3-\delta}$	1	7	1400	SSR	[12]
$Ba(Mg_{0.2}Zn_{0.2}Ti_{0.2}W_{0.2}Mo_{0.2})O_3$	1	5	1600	SSR	[13]
$Ba(Zn_{0.2}Yb_{0.2}Y_{0.2}W_{0.2}Mo_{0.2})O_3$	1	5	1600	SSR	[13]
$Ba(Mg_{0.2}Zn_{0.2}Nb_{0.2}Ta_{0.2}W_{0.2})O_3$	1	5	1600	SSR	[13]
$Ba(Zn_{0.2}Yb_{0.2}Ti_{0.2}Nb_{0.2}W_{0.2})O_3$	1	5	1600	SSR	[13]
$Ba(Zn_{0.2}Yb_{0.2}Nb_{0.2}Ta_{0.2}Sb_{0.2})O_3$	1	5	1600	SSR	[13]
$Ba(Zn_{0.2}Ti_{0.2}Zr_{0.2}Hf_{0.2}W_{0.2})O_3$	1	5	1600	SSR	[13]
$Ba(Zn_{0.2}Ti_{0.2}Zr_{0.2}Nb_{0.2}Ta_{0.2})O_3$	1	5	1600	SSR	[13]
$Ba(Yb_{0.2}Y_{0.2}Ga_{0.2}Nb_{0.2}W_{0.2})O_3$	1	5	1600	SSR	[13]
$Ba(Yb_{0.2}Y_{0.2}Ti_{0.2}Zr_{0.2}W_{0.2})O_3$	1	5	1600	SSR	[13]
$Ba(Yb_{0.2}Y_{0.2}Ti_{0.2}Nb_{0.2}Ta_{0.2})O_3$	1	5	1600	SSR	[13]
$Ba(Yb_{0.2}Zr_{0.2}Ti_{0.2}Sn_{0.2}Nb_{0.2})O_3$	1	5	1600	SSR	[13]
$Ba(Zr_{0.2}Ti_{0.2}Ce_{0.2}Hf_{0.2}Sn_{0.2})O_3$	1	5	1600	SSR	[13]
$La_{0.5}Nd_{0.5}(Cr_{0.2}Mn_{0.2}Fe_{0.2}Co_{0.2}Ni_{0.2})O_3$	2	5	1300	SSR	[14]

$La(Cr_{0.2}Mn_{0.2}Fe_{0.2}Co_{0.2}Ni_{0.2})O_3$	1	5	1300	SSR	[14]
$Nd(Cr_{0.2}Mn_{0.2}Fe_{0.2}Co_{0.2}Ni_{0.2})O_3$	1	5	1300	SSR	[14]
$(Ca_{0.2}Sr_{0.2}Ba_{0.2}Pb_{0.2}La_{0.2})TiO_3$	5	1	1200	SSR	[15]
$(Bi_{1/6}Na_{1/6}Sr_{1/6}Ba_{1/6}Pb_{1/6}Ca_{1/6})TiO_3$	6	1	1260	SSR	[16]
$(Bi_{1/6}La_{1/6}Na_{1/6}K_{1/6}Sr_{1/6}Ba_{1/6})TiO_3$	6	1	1260	SSR	[16]
$(La_{0.2}Li_{0.2}Ba_{0.2}Sr_{0.2}Ca_{0.2})TiO_3$	5	1	1500	SSR	[17]
$La(Ti_{1/7}Co_{1/7}Cr_{1/7}Cu_{1/7}Fe_{1/7}Mn_{1/7}Ni_{1/7})O_3$	1	7	1100	SSR	[18]
$La_{0.5}Nd_{0.5}(Ti_{1/7}Co_{1/7}Cr_{1/7}Cu_{1/7}Fe_{1/7}Mn_{1/7}Ni_{1/7})O_3$	2	7	1100	SSR	[18]
$Sm(Ti_{1/7}Co_{1/7}Cr_{1/7}Cu_{1/7}Fe_{1/7}Mn_{1/7}Ni_{1/7})O_3$	1	7	1100	SSR	[18]
$Sm(Co_{0.2}Cr_{0.2}Fe_{0.2}Mn_{0.2}Ni_{0.2})O_3$	1	5	1100	SSR	[18]
$La(Co_{0.2}Cr_{0.2}Fe_{0.2}Mn_{0.2}Ni_{0.2})O_3$	1	5	1100	SSR	[18]
$La(Ti_{1/6}Co_{1/6}Cr_{1/6}Fe_{1/6}Mn_{1/6}Ni_{1/6})O_3$	1	6	1100	SSR	[18]
$La(Ti_{1/8}Co_{1/8}Cr_{1/8}Cu_{1/8}Fe_{1/8}Mn_{2/8}Ni_{1/8})O_3$	1	7	1100	SSR	[18]
$La(Co_{1/7}Cr_{1/7}Fe_{1/7}Mn_{3/7}Ni_{1/7})O_3$	1	5	1100	SSR	[18]
$La(Ti_{1/6}Co_{2/6}Cu_{1/6}Mn_{1/6}Ni_{1/6})O_3$	1	5	1100	SSR	[18]
$La0.8Ca_{0.2}(Co_{0.2}Cr_{0.2}Fe_{0.2}Mn_{0.2}Ni_{0.2})O_3$	2	5	1100	SSR	[18]
$(Na_{0.2}Bi_{0.2}Ba_{0.2}Sr_{0.2}Ca_{0.2})TiO_3$	5	1	1275	SSR	[19]
$(Na_{0.2}Bi_{0.2}Ba_{0.2}Sr_{0.2}Ca_{0.2})TiO_3$	5	1	1300	SSR	[20]
$(Bi_{0.2}K_{0.2}Na_{0.2}Ba_{0.2}Ca_{0.2})TiO_3$	5	1	1150	SSR	[21]
$(Ba_{0.2}Sr_{0.2}Ca_{0.2}Mg_{0.2}Pb_{0.2})(Ti_{0.5}Zr_{0.5})O_3$	5	2	1400	SSR	[22]
$(Ba_{0.2}Sr_{0.2}Ca_{0.2}Mg_{0.2}Pb_{0.2})(Ti_{0.33}Zr_{0.33}Hf_{0.33})O_3$	5	3	1400	SSR	[22]
$(Ba_{0.2}Sr_{0.2}Ca_{0.2}Mg_{0.2}Pb_{0.2})(Ti_{0.25}Zr_{0.25}Hf_{0.25}Sn_{0.25})O_3$	5	4	1400	SSR	[22]
$(Ba_{0.2}Sr_{0.2}Ca_{0.2}Mg_{0.2}Pb_{0.2})(Ti_{0.2}Zr_{0.2}Hf_{0.2}Sn_{0.2}Mn_{0.2})O_3$	5	5	1400	SSR	[22]
$(Ca_{0.2}Sr_{0.2}Ba_{0.2}La_{0.2}Pb_{0.2})TiO_3$	5	1	1300	SSR	[23]
$Ba(Mg_{0.17}Zn_{0.17}Ti_{0.21}Nb_{0.22}W_{0.23})O_3$	1	5	1500	SSR	[24]
$Ba(Mg_{0.15}Yb_{0.17}Ti_{0.2}1Nb_{0.23}Ta_{0.24})O_3$	1	5	1500	SSR	[24]
$Ba(Mg_{0.16}Y_{0.19}Yb_{0.19}Nb_{0.22}W_{0.24})O_3$	1	5	1500	SSR	[24]
$Ba(Mg_{0.22}Zn_{0.23}Nb_{0.20}W_{0.17}Mo_{0.18})O_3$	1	5	1500	SSR	[24]

$Ba(Mg_{0.23}Yb_{0.22}Ti_{0.21}W_{0.17}Mo_{0.17})O_3$	1	5	1500	SSR	[24]
$Ba(Mg_{0.24}Yb_{0.22}Nb_{0.19}Ta_{0.19}W_{0.16})O_3$	1	5	1500	SSR	[24]
$Ba(Mg_{0.25}Ti_{0.21}Zr_{0.20}Nb_{0.18}W_{0.16})O_3$	1	5	1500	SSR	[24]
$Ba(Y_{0.22}Yb_{0.22}Dy_{0.22}W_{0.17}Mo_{0.17})O_3$	1	5	1500	SSR	[24]
$(BaNaBi)_{0.205}(SrCa)_{0.1925}TiO_3$	5	1	1275	SSR	[25]
$(BaNaBi)_{0.205}(SrCa)_{0.1925}Ti_{0.92}Hf_{0.08}O_3$	5	2	1275	SSR	[25]
$(BaNaBi)_{0.205}(SrCa)_{0.1925}Ti_{0.92}Nb_{0.08}O_3$	5	2	1275	SSR	[25]
$Bi_{0.2}Na_{0.2}Ba_{0.2}Sr_{0.2}Ca_{0.2}TiO_3$	5	1	1300	SSR	[26]
$Bi(Zn_{0.2}Mg_{0.2}Al_{0.2}Sn_{0.2}Zr_{0.2})$ O_3	1	5	1025	SSR	[27]
$Sm(Cr_{0.2}Mn_{0.2}Fe_{0.2}Co_{0.2}Ni_{0.2})O_3$	1	5	1400	SSR	[28]
$Eu(Cr_{0.2}Mn_{0.2}Fe_{0.2}Co_{0.2}Ni_{0.2})O_3$	1	5	1400	SSR	[28]
$Gd(Cr_{0.2}Mn_{0.2}Fe_{0.2}Co_{0.2}Ni_{0.2})O_3$	1	5	1400	SSR	[28]
$Li_x(Li_{0.2}La_{0.2}Ca_{0.2}Sr_{0.2}Ba_{0.2})Ti_{1-x}Al_xO_3$ (x=0-0.2)	5	2	1325	SSR	[29]
$(Bi_{0.2}Na_{0.2}Ba_{0.2}Sr_{0.2}Ca_{0.2})TiO_3$	5	1	1250	SSR	[30]
$(Bi_{0.2}Li_{0.2}Sr_{0.2}Ba_{0.2}Pb_{0.2})TiO_3$	5	1	1150	SSR	[30]
$(Bi_{0.2}Na_{0.2}Ba_{0.2}Sr_{0.2}Pb_{0.2})TiO_3$	5	1	1200	SSR	[30]
$(Bi_{0.2}K_{0.2}Ba_{0.2}Sr_{0.2}Pb_{0.2})TiO_3$	5	1	1200	SSR	[30]
$(Bi_{0.2}Ag_{0.2}Ba_{0.2}Sr_{0.2}Pb_{0.2})TiO_3$	5	1	1150	SSR	[30]
$(Gd_{0.2}Dy_{0.2}Ho_{0.2}Er_{0.2}Tb_{0.2})FeO_3$	5	1	1400	SSR	[31]
$Sr(Zr_{0.2}Sn_{0.2}Hf_{0.2}Ti_{0.2}Nb_{0.2})O_3$	1	5	1620	SSR	[32]
$(La_{0.2}Nd_{0.2}Pr_{0.2}Sm_{0.2}Eu_{0.2})_{1-x}Sr_xCoO_3$ (x=0-0.1)	6	1	1350	SSR	[33]
$(Na_{0.2}Bi_{0.2}Ca_{0.2}Sr_{0.2}Ba_{0.2})TiO_{3-x}$	5	1	1220	SSR	[34]
$Sr_{0.9}La_{0.1}(Zr_{0.25}Sn_{0.25}Ti_{0.25}Hf_{0.25})O_3$	2	4	1400	SSR	[35]
$La(Fe_{0.2}Co_{0.2}Ni_{0.2}Cr_{0.2}Mn_{0.2})O_3$	1	5	1500	SSR	[36]
$(La_{0.5}K_{0.5})_x((Bi_{0.5}Na_{0.5})_{0.25}Ba_{0.25}Sr_{0.25}Ca_{0.25})_{1-x}TiO_3$ (x=0.08-0.32)	7	1	1300	SSR	[37]
$(Bi_{0.2}Na_{0.2}Ba_{0.2}Sr_{0.2}Ca_{0.2})TiO_{3-x}$	5	1	1300	SSR	[38]
$(La_{0.2}Ca_{0.2}Sr_{0.2}Ba_{0.2}Y_{0.2})MnO_3$	5	1	1300	SSR	[39]
$(Nd_{0.2}Ca_{0.2}Sr_{0.2}Ba_{0.2}Y_{0.2})MnO_3$	5	1	1300	SSR	[39]

$(Ho_{0.2}Ca_{0.2}Sr_{0.2}Ba_{0.2}Y_{0.2})MnO_3$	5	1	1300	SSR	[39]
$(Lu_{0.2}Ca_{0.2}Sr_{0.2}Ba_{0.2}Y_{0.2})MnO_3$	5	1	1300	SSR	[39]
$Sm(Cr_{0.2}Mn_{0.2}Fe_{0.2}Co_{0.2}Ni_{0.2})O_3$	1	5	1400	SSR	[40]
$Eu(Cr_{0.2}Mn_{0.2}Fe_{0.2}Co_{0.2}Ni_{0.2})O_3$	1	5	1400	SSR	[40]
$Gd(Cr_{0.2}Mn_{0.2}Fe_{0.2}Co_{0.2}Ni_{0.2})O_3$	1	5	1400	SSR	[40]
$Bi(Li_{0.2}Y_{0.2}Mg_{0.2}Ti_{0.2}Ta_{0.2})O_3$	1	5	1320	SSR	[41]
$(Ca_{1/3}Sr_{1/3}Ba_{1/3})(Y_{1/4}Zr_{1/2}Nb_{1/4})O_3$	3	3	1500	SSR	[42]
$(Pb_{1/6}Ba_{1/6}Sr_{1/6}Ca_{1/6}Na_{1/6}Bi_{1/6})TiO_3$	6	1	1300	SSR	[43]
$Pb(Ni_{1/6}W_{1/6}Mn_{1/6}Nb_{1/6}Zr_{1/6}Ti_{1/6})O_3$	1	6	860	SSR	[44]
$Sr_{0.9}La_{0.1}(Zr_{0.25}Sn_{0.25}Ti_{0.25}Hf_{0.25})O_3$	2	4	1550	SSR	[45]
$Sr_{0.9}La0.1(Zr_{0.2}Sn_{0.2}Ti_{0.2}Hf_{0.2}Nb_{0.2})O_3$	2	5	1550	SSR	[45]
$Sr_{0.9}La0.1(Zr_{0.2}Sn_{0.2}Ti_{0.2}Hf_{0.2}Mn_{0.2})O_3$	2	5	1550	SSR	[45]
$Sr_{0.9}La0.1(Zr_{1/6}Sn_{1/6}Ti_{1/6}Hf_{1/6}Mn_{1/6}Nb_{1/6})O_3$	2	6	1550	SSR	[45]
$(La_{0.25}Sr_{0.25}Ba_{0.25}Na_{0.25})(Ti_{0.5}Sn_{0.5})O_{3-\delta}$	4	2	1500	SSR	[46]
$(La_{0.25}Sr_{0.25}Ba_{0.25}Na_{0.25})(Ti_{0.5}Zr_{0.5})O_{3-\delta}$	4	2	1500	SSR	[46]
$(La_{0.25}Sr_{0.25}Ba_{0.25}Na_{0.25})(Ti_{0.5}Hf_{0.5})O_{3-\delta}$	4	2	1500	SSR	[46]
$(Na_{0.2}Bi_{0.2}Ca_{0.2}Sr_{0.2}Ba_{0.2})TiO_{3-x}$	5	1	1225	SSR	[47]
$[(K_{0.2}Na_{0.8})_{0.8}Li_{0.08}Ba_{0.02}Bi_{0.1}]$ $(Nb_{0.68}Sc_{0.02}Hf_{0.08}Zr_{0.1}Ta_{0.08}Sb_{0.04})O_3$	5	6	1230	SSR	[48]
$(Bi_{0.5}Na_{0.5})(Ti_{1-2x}Fe_xNb_x)O_3$	2	3	1250	SSR	[48]
$La(Cu_{0.2}Ni_{0.2}Co_{0.2}Ga_{0.2}Nb_{0.2})O_{3-\delta}$	1	5	1050	SSR	[49]
$La(Cu_{0.2}Ni_{0.2}Co_{0.2}Ga_{0.2}Ta_{0.2})O_{3-\delta}$	1	5	1050	SSR	[49]
$La(Cu_{0.2}Ni_{0.2}Co_{0.2}Fe_{0.2}Nb_{0.2})O_{3-\delta}$	1	5	1050	SSR	[49]
$La(Cu_{0.2}Ni_{0.2}Fe_{0.2}Ga_{0.2}Nb_{0.2})O_{3-\delta}$	1	5	1050	SSR	[49]
$La(Cu_{0.2}Ni_{0.2}Fe_{0.2}Ga_{0.2}Ta_{0.2})O_{3-\delta}$	1	5	1050	SSR	[49]
$La(Cu_{0.2}Ni_{0.2}Co_{0.2}Fe_{0.2}Ta_{0.2})O_{3-\delta}$	1	5	1050	SSR	[49]
$SrNi_{0.2}Nb_{0.2}W_{0.2}Ti_{0.2}Mn_{0.2}O_3$	1	5	1600	SSR	[50]
$SrNi_{0.2}Nb_{0.2}W_{0.2}Ti_{0.2}Al_{0.2}O_3$	1	5	1600	SSR	[50]
$SrNi_{0.2}Nb_{0.2}W_{0.2}Ti_{0.2}Fe_{0.2}O_3$	1	5	1600	SSR	[50]
$La_{1-x}Sr_x(Co_{0.2}Cr_{0.2}Fe_{0.2}Mn_{0.2}Ni_{0.2})O_{3-\delta}$	2	5	1000	Sol-gel	[51]

(x = 0.1-0.5)					
La-$(Fe_{0.2}Co_{0.2}Ni_{0.2}Cu_{0.2}Cr_{0.2})O_3$	1	5	850	Sol-gel	[52]
$Ba(Zr_{0.2}Sn_{0.2}Ti_{0.2}Hf_{0.2}Nb_{0.2})O_3$	1	5	800	Sol-gel	[53]
$(Pr_{1/6}La_{1/6}Nd_{1/6}Ba_{1/6}Sr_{1/6}Ca_{1/6})CoO_{3-\delta}$	6	1	1000	Sol-gel	[54]
$(La_{0.2}Y_{0.2}Nd_{0.2}Gd_{0.2}Sr_{0.2})CrO_3$	5	1	1250	Sol-gel	[55]
$(La_{0.2}Nd_{0.2}Sm_{0.2}Y_{0.2}Gd_{0.2})MnO_3$	5	1	1200	Sol-gel	[56]
$(La_{0.2}Nd_{0.2}Pr_{0.2}Sr_{0.2}Ba_{0.2})MnO_3$	5	1	1200	Sol-gel	[56]
$(La_{0.2}Nd_{0.2}Sm_{0.2}Ca_{0.2}Sr_{0.2})MnO_3$	5	1	1200	Sol-gel	[56]
$(La_{0.2}Pr_{0.2}Ca_{0.2}Sr_{0.2}Ba_{0.2})MnO_3$	5	1	1200	Sol-gel	[56]
$(La_{0.2}Nd_{0.2}Ca_{0.2}Sr_{0.2}Ba_{0.2})MnO_3$	5	1	1200	Sol-gel	[56]
$Ba(Ti_{0.2}Zr_{0.2}Sn_{0.2}Hf_{0.2}Ce_{0.2})O_3$	1	5	900	Sol-gel	[57]
$La(Fe_{0.2}Co_{0.2}Mn_{0.2}Ni_{0.2}Cr_{0.2})O_3$	1	5	950	Sol-gel	[58]
$La_{0.5}Sr_{1.5}Mn_{0.2}Fe_{0.2}Ni_{0.2}Cu_{0.2}Co_{0.2}O_4$	2	5	1000	Sol-gel	[59]
$(La_{0.2}Sr_{0.2}Pr_{0.2}Gd_{0.2}Sm_{0.2})Co_{0.2}Fe_{0.8}O_{3-\delta}$	5	2	1300	Sol-gel	[60]
$(La_{0.2}Sr_{0.2}Pr_{0.2}Gd_{0.2}Y_{0.2})Co_{0.2}$Fe0.8$O_{3-\delta}$	5	2	1300	Sol-gel	[60]
$(La_{0.2}Sr_{0.2}Pr_{0.2}Gd_{0.2}Ba_{0.2})Co_{0.2}$Fe0.8$O_{3-\delta}$	5	2	1300	Sol-gel	[60]
$(La_{0.2}Sr_{0.2}Pr_{0.2}Y_{0.2}Sm_{0.2})Co_{0.2}$Fe0.8$O_{3-\delta}$	5	2	1300	Sol-gel	[60]
$(La_{0.2}Sr_{0.2}Pr_{0.2}Y_{0.2}Nd_{0.2})Co_{0.2}Fe_{0.8}O_{3-\delta}$	5	2	1300	Sol-gel	[60]
$(La_{0.2}Sr_{0.2}Pr_{0.2}Y_{0.2}Ba_{0.2})Co_{0.2}Fe_{0.8}O_{3-\delta}$	5	2	1300	Sol-gel	[60]
$(La_{0.2}Sr_{0.2}Gd_{0.2}Y_{0.2}Ba_{0.2})Co_{0.2}Fe_{0.8}O_{3-\delta}$	5	2	1300	Sol-gel	[60]
$La_{0.6}Sr_{0.4}[Co_{0.2}(Fe, Ni, Mn)_{0.8}]O_3$	2	4	850	Sol-gel	[61]
$La_{0.6}Sr_{0.4}[Co_{0.2}(Fe,Ni,Mn,Mg)_{0.8}]O_3$	2	5	850	Sol-gel	[61]
$LaMg_{0.2}Fe_{0.2}Co_{0.2}Ni_{0.2}Mn_{0.2}O_2$	1	5	-	Sol-gel	[62]
$LaMg_{0.2}Fe_{0.2}Co_{0.2}Ni_{0.2}Cu_{0.2}O_2$	1	5	-	Sol-gel	[62]
$Ba(Sn_{0.16}Zr_{0.24}Ce_{0.35}Y_{0.1}Yb_{0.1}Dy_{0.05})O_{3-\delta}$	1	6	1550	Sol-gel	[63]
$Pr_{1-x}Sr_x(Cr_{0.2}Mn_{0.2}Fe_{0.2}Co_{0.2}Ni_{0.2})O_{3-\delta}$ (x = 0-0.5)	2	5	1400	Sol-gel	[64]
$Ca_{0.1}La_{0.02}Gd_{0.02}Bi_{0.02}Ba_{0.42}Sr_{0.42}Co_{0.8}Fe_{0.2}O_{3-\delta}$	6	2	800	Sol-gel	[65]
$Ba_{0.5}Sr_{0.5}Co_{0.736}Fe_{0.184}Zr_{0.02}Ni_{0.02}Cu_{0.02}Al_{0.02}O_{3-\delta}$	2	6	800	Sol-gel	[65]
$(Ca_{0.1}La_{0.02}Gd_{0.02}Bi_{0.02}Ba_{0.42}Sr_{0.42})(Co_{0.736}Fe_0$	6	6	800	Sol-gel	[65]

$_{.184}Zr_{0.02}Ni_{0.02}Cu_{0.02}Al_{0.02})O_{3-\delta}$					
$Ba(Ti_{0.2}Zr_{0.2}Sn_{0.2}Hf_{0.2}Ce_{0.2})O_3$	1	5	1200	Sol-gel	[66]
$La_xSr_{1-x}(CoCrFeMnNi)O_{3-\delta}$ (x = 0.5, 0.7, 0.9)	2	5	1000	Solution combustion	[67]
$(Y_{0.2}Nd_{0.2}Sm_{0.2}Eu_{0.2}Er_{0.2})AlO_3$	5	1	1500	Co-precipitation	[68]
$La(Co, Cr, Fe, Mn, Ni, Al_x)_{1/(5+x)}O_{3-\delta}$ (x = 0-0.8)	1	6	1200	Co-precipitation	[69]
$La(Co_{0.2}Cr_{0.2}Fe_{0.2}Mn_{0.2}Ni_{0.2})O_3$	1	5	1500	Co-precipitation	[70]
$La(CrMnFeCo_2Ni)O_3$	1	5	750	Co-precipitation	[71]
$Ba(Ce_{0.2}Zr_{0.2}Gd_{0.2}La_{0.2}Y_{0.2})O_{2.7}$	1	5	1300	Co-precipitation	[72]
$Sr((Zr_{0.94}Y_{0.06})_{0.2}Sn_{0.2}Ti_{0.2}Hf_{0.2}Mn_{0.2})O_{3-x}$	1	6	1450	SPS	[73]
$(Bi_{0.2}Na_{0.2}K_{0.2}Ba_{0.2}Ca_{0.2})TiO_3$	5	1	840	Flash-sintering	[74]
$Sr(Ti_{0.2}Y_{0.2}Zr_{0.2}Sn_{0.2}Hf_{0.2})O_{3-\delta}$	1	5	1470	Flash-sintering	[75]
$(Na_{0.2}La_{0.2}Ca_{0.2}Sr_{0.2}Ba_{0.2})TiO_{3-x}$	5	1	1200	Mechanochemical	[76]
$(Gd_{0.2}Nd_{0.2}La_{0.2}Sm_{0.2}Y_{0.2})CoO_3$	5	1	1200	Coprecipitation-hydrothermal	[77]
$(Bi_{0.2}Na_{0.2}Ba_{0.2}Ca_{0.2}Sr_{0.2})TiO_3$	5	1	1200	Hydrothermal	[78]

(La,Ca)(Ti,Fe,Mn)O_3	2	3	1400	Hydrothermal	[79]
$Gd_{0.2}La_{0.2}Nd_{0.2}Sm_{0.2}Y_{0.2})(Co_{0.2}Cr_{0.2}Fe_{0.2}Mn_{0.2}Ni_{0.2})O_3$	5	5	1200	Nebulized spray pyrolysis	[80]
$(Gd_{0.2}La_{0.2}Nd_{0.2}Sm_{0.2}Y_{0.2})CoO_3$	5	1	1200	Nebulized spray pyrolysis	[80]
$(Gd_{0.2}La_{0.2}Nd_{0.2}Sm_{0.2}Y_{0.2})CrO_3$	5	1	1200	Nebulized spray pyrolysis	[80]
$(Gd_{0.2}La_{0.2}Nd_{0.2}Sm_{0.2}Y_{0.2})FeO_3$	5	1	1200	Nebulized spray pyrolysis	[80]
$(Gd_{0.2}La_{0.2}Nd_{0.2}Sm_{0.2}Y_{0.2})MnO_3$	5	1	1200	Nebulized spray pyrolysis	[80]
$(Gd_{0.2}La_{0.2}Nd_{0.2}Sm_{0.2}Y_{0.2})NiO_3$	5	1	1200	Nebulized spray pyrolysis	[80]
$Gd(Co_{0.2}Cr_{0.2}Fe_{0.2}Mn_{0.2}Ni_{0.2})O_3$	1	5	1200	Nebulized spray pyrolysis	[80]
$La(Co_{0.2}Cr_{0.2}Fe_{0.2}Mn_{0.2}Ni_{0.2})O_3$	1	5	1200	Nebulized spray pyrolysis	[80]
$Nd(Co_{0.2}Cr_{0.2}Fe_{0.2}Mn_{0.2}Ni_{0.2})O_3$	1	5	1200	Nebulized spray pyrolysis	[80]
$Sm(Co_{0.2}Cr_{0.2}Fe_{0.2}Mn_{0.2}Ni_{0.2})O_3$	1	5	1200	Nebulized spray pyrolysis	[80]
$Y(Co_{0.2}Cr_{0.2}Fe_{0.2}Mn_{0.2}Ni_{0.2})O_3$	1	5	1200	Nebulized	[80]

				spray pyrolysis	
$La(Cr_{0.2}Mn_{0.2}Fe_{0.2}Co_{0.2}Ni_{0.2})O_3$	1	5	1200	Nebulized spray pyrolysis	[81]
$Gd(Cr_{0.2}Mn_{0.2}Fe_{0.2}Co_{0.2}Ni_{0.2})O_3$	1	5	1200	Nebulized spray pyrolysis	[81]
$(La_{0.2}Nd_{0.2}Sm_{0.2}Gd_{0.2}Y_{0.2})(Cr_{0.2}Mn_{0.2}Fe_{0.2}Co_{0.2}Ni_{0.2})O_3$	5	5	1200	Nebulized spray pyrolysis	[81]
$(Gd_{0.2}La_{0.2}Nd_{0.2}Sm_{0.2}Y_{0.2})CoO_3$	5	1	1200	Nebulized spray pyrolysis	[82]
$(Gd_{0.2}La_{0.2}Nd_{0.2}Sm_{0.2}Y_{0.2})CrO_3$	5	1	1200	Nebulized spray pyrolysis	[82]
$(Gd_{0.2}La_{0.2}Nd_{0.2}Sm_{0.2}Y_{0.2})FeO_3$	5	1	1200	Nebulized spray pyrolysis	[82]
$(Gd_{0.2}La_{0.2}Nd_{0.2}Sm_{0.2}Y_{0.2})(1\text{-}x)CaxFeO_3$	6	1	1200	Nebulized spray pyrolysis	[83]
$(La_{0.2}Pr_{0.2}Nd_{0.2}Sm_{0.2}Eu_{0.2})NiO_3$	5	1	735	PLD	[84]
$Tb(Fe_{0.2}Mn_{0.2}Co_{0.2}Cr_{0.2}Ni_{0.2})O_3$	1	5	840	PLD	[84]
$La_{1-x}Sr_x(Cr_{0.2}Mn_{0.2}Fe_{0.2}Co_{0.2}Ni_{0.2})O_3$ (x=0-0.5)	2	5	700	PLD	[85]
$LaCr_{0.2}Mn_{0.2}Fe_{0.2}Co_{0.2}Ni_{0.2}O_{3-\delta}$	1	5	1500	PLD	[86]
$Ba(Ti_{0.2}Sn_{0.2}Zr_{0.2}Hf_{0.2}Nb_{0.2})O_3$	1	5	750	PLD	[87]
$Pb(Hf_{0.2}Zr_{0.2}Ti_{0.2}Nb_{0.2}Mn_{0.2})O_3$	1	5	1200	PLD	[88]
$Pb(Hf_{0.2}Zr_{0.2}Ti_{0.2}Nb_{0.2}Al_{0.2})O_3$	1	5	1200	PLD	[88]
$(La_{0.2}Lu_{0.2}Y_{0.2}Gd_{0.2}Ce_{0.2})AlO_3$	5	1	775	PLD	[89]
$La(Cr_{0.2}Mn_{0.2}Fe_{0.2}Co_{0.2}Ni_{0.2})O_3$	1	5	700	Pechini	[90]

				technique	

SSR: solid-state reaction, SPS: spark plasma sintering, PLD: pulsed laser deposition.

References

[1] S. Zhou, Y. Pu, Q. Zhang, R. Shi, X. Guo, W. Wang, J. Ji, T. Wei, T. Ouyang, "Microstructure and dielectric properties of high entropy $Ba(Zr_{0.2}Ti_{0.2}Sn_{0.2}Hf_{0.2}Me_{0.2})O_3$ perovskite oxides" *Ceram. Int.* **2020**, *46*, 7430–7437.

[2] Q. Du, J. Yan, X. Zhang, J. Li, X. Liu, J. Zhang, X. Qi, "Phase evolution and dielectric properties of $Ba(Ti_{1/6}Sn_{1/6}Zr_{1/6}Hf_{1/6}Nb_{1/6}Ga_{1/6})O_3$ high-entropy perovskite ceramics" *J. Mater. Sci. Mater. Electron.* **2020**, *31*, 7760–7765.

[3] Y. Pu, Q. Zhang, R. Li, E. Al., "Dielectric properties and electrocaloric effect of high-entropy $(Na_{0.2}Bi_{0.2}Ba_{0.2}Sr_{0.2}Ca_{0.2})TiO_3$ ceramic" *Appl. Phys. Lett.* **2019**, *115*, 223901.

[4] S. Jiang, T. Hu, J. Gild, N. Zhou, J. Nie, M. Qin, T. Harrington, K. Vecchio, J. Luo, "A new class of high-entropy perovskite oxides" *Scr. Mater.* **2018**, *142*, 116–120.

[5] D. A. Vinnik, E. A. Trofimov, V. E. Zhivulin, S. A. Gudkova, O. V Zaitseva, D. A. Zherebtsov, A. Y. Starikov, D. P. Sherstyuk, A. A. Amirov, A. V Kalgin, S. V Trukhanov, F. V Podgornov, "High Entropy Oxide Phases with Perovskite Structure" *Nanomaterials* **2020**, *10*, 268.

[6] Y. Sharma, Q. Zheng, A. R. Mazza, E. Skoropata, T. Heitmann, Z. Gai, B. Musico, P. F. Miceli, B. C. Sales, V. Keppens, M. Brahlek, T. Z. Ward, "Magnetic anisotropy in single-crystal high-entropy perovskite oxide $La(Cr_{0.2}Mn_{0.2}Fe_{0.2}Co_{0.2}Ni_{0.2})O_3$ film" *Phys. Rev. Mater.* **2020**, *4*, 14404.

[7] J. Yan, D. Wang, X. Zhang, J. Li, Q. Du, X. Liu, J. Zhang, X. Qi, "A high-entropy perovskite titanate lithium-ion battery anode" *J. Mater. Sci.* **2020**, *55*, 6942–6951.

[8] Y. Sharma, B. L. Musico, X. Gao, C. Hua, A. F. May, A. Herklotz, A. Rastogi, D. Mandrus, J. Yan, H. N. Lee, M. F. Chisholm, V. Keppens, T. Z. Ward, "Single-crystal high entropy perovskite oxide epitaxial films" *Phys. Rev. Mater.* **2018**, *2*, 060404 (1–6).

[9] W. Qiao, J. Mei, M. Bai, Y. Gao, X. Zhu, Y. Hu, X. Lou, "Microstructural and

dielectric characteristics of A-site high-entropy oxide ceramics with a perovskite structure" *Ceram. Int.* **2024**, *50*, 51812–51819.

[10] J. Zhou, P. Zheng, W. Bai, Q. Fan, L. Zheng, Y. Zhang, "Breaking the Mutual Constraint between Polarization and Voltage Resistance with Nanograined High-Entropy Ceramic" *ACS Appl. Mater. Interfaces* **2024**, *16*, 2530−2538.

[11] R. Banerjee, S. Chatterjee, M. Ranjan, T. Bhattacharya, S. Mukherjee, "High-Entropy Perovskites: An Emergent Class of Oxide Thermoelectrics with Ultralow Thermal Conductivity" *ACS Sustain. Chem. Eng.* **2020**, *8*, 17022−17032.

[12] M. Gazda, T. Miruszewski, D. Jaworski, A. Mielewczyk-gryn, W. Skubida, I. Szpunar, E. Dzik, S. Wachowski, P. Winiarz, K. Dzierzgowski, Ł. Marcin, "Novel Class of Proton Conducting Materials - High Entropy Oxides" *Mater. Lett.* **2020**, *2*, 1315−1321.

[13] L. Tang, Z. Li, K. Chen, E. Al., "High-entropy oxides based on valence combinations : design and practice" *J. Am. Ceram. Soc.* **2021**, *104*, 1953–1958.

[14] V. E. Zhivulin, E. A. Trofimov, S. A. Gudkova, I. Y. Pashkeev, A. Y. Punda, M. Gavrilyak, O. V Zaitseva, S. V Taskaev, F. V Podgornov, M. A. Darwish, M. A. Almessiere, Y. Slimani, A. Baykal, S. V Trukhanov, A. V Trukhanov, D. A. Vinnik, "Polysubstituted High-Entropy [LaNd]($Cr_{0.2}Mn_{0.2}Fe_{0.2}Co_{0.2}Ni_{0.2}$)$O_3$ Perovskites: Correlation of the Electrical and Magnetic Properties" *Nanomaterials* **2021**, *11*, 1014.

[15] Y. Zheng, M. Zou, W. Zhang, D. Yi, J. Lan, C. Nan, Y. Lin, "Electrical and thermal transport behaviours of high-entropy perovskite thermoelectric oxides" *J. Adv. Ceram.* **2021**, *10*, 377–384.

[16] W. Liu, F. Li, G. Chen, G. Li, H. Shi, L. Li, Y. Guo, "Comparative study of phase structure , dielectric properties and electrocaloric effect in novel high- entropy ceramics" *J. Mater. Sci.* **2021**, *56*, 18417–18429.

[17] Z. Xiaoyan, L. Xinyue, Y. Jinhua, E. Al., "Preparation and Property of High-entropy" *J. Inorg. Mater.* **2021**, *36*, 379–385.

[18] J. Cedervall, R. Clulow, H. L. B. Bostr, M. S. Andersson, R. Mathieu, P. Beran, R. I. Smith, J. Tseng, M. Sahlberg, P. Berastegui, S. Shafeie, "Phase stability and structural transitions in compositionally complex $LnMO_3$ perovskites" *J. Solid State Chem.*

2021, *300*, 122213.

[19] K. Bunpang, W. Somsri, D. P. Cann, “Enhanced temperature stability and reduced tan δ in B-site modified titanate-based high-entropy perovskite oxides” *J. Am. Ceram. Soc.* **2025**, *108*, e20353.

[20] Y. Ning, Y. Pu, Q. Zhang, S. Zhou, C. Wu, L. Zhang, “Achieving high energy storage properties in perovskite oxide via high-entropy design” *Ceram. Int.* **2023**, *49*, 12214–12223.

[21] A. Kumar, R. Vaish, G. Singh, “Solar energy enhanced tribocatalytic dye degradation using high entropy perovskite ceramics” *Sol. Energy* **2025**, *301*, 113906.

[22] O. V Zaitseva, S. A. Gudkova, E. A. Trofimov, “New high-entropy oxide phases with the perovskite structure” *IOP Conf. Ser. Mater. Sci. Eng.* **2021**, *1014*, 012060.

[23] P. Zhang, Z. Lou, M. Qin, J. Xu, J. Zhu, Z. Shi, Q. Chen, M. J. Reece, H. Yan, F. Gao, “High-entropy $(Ca_{0.2}Sr_{0.2}Ba_{0.2}La_{0.2}Pb_{0.2})TiO_3$ perovskite ceramics with A-site short-range disorder for thermoelectric applications Ping” *J. Mater. Sci. Technol.* **2022**, *97*, 182–189.

[24] J. Ma, K. Chen, C. Li, X. Zhang, L. An, “High-entropy stoichiometric perovskite oxides based on valence combinations” *Ceram. Int.* **2021**, *47*, 24348–24352.

[25] Y. Ning, Y. Pu, Z. Chen, L. Zhang, C. Wu, X. Zhang, B. Wang, “Novel high-entropy relaxors with ultrahigh energy-storage efficiency and density” *Chem. Eng. J.* **2023**, *476*, 146673.

[26] J. Guo, H. Yu, Y. Ren, H. Qi, X. Yang, Y. Deng, S. Zhang, “Multi-symmetry high-entropy relaxor ferroelectric with giant capacitive energy storage” *Nano Energy* **2023**, *112*, 108458.

[27] S. Zhou, Y. Pu, X. Zhang, Y. Shi, Z. Gao, Y. Feng, G. Shen, X. Wang, D. Wang, “High energy density , temperature stable lead-free ceramics by introducing high entropy perovskite oxide” *Chem. Eng. J.* **2022**, *427*, 131684.

[28] D. A. Vinnik, V. E. Zhivulin, E. A. Trofimov, S. A. Gudkova, A. Y. Punda, A. N. Valiulina, M. Gavrilyak, O. V Zaitseva, S. V Taskaev, M. U. Khandaker, A. Alqahtani, D. A. Bradley, M. I. Sayyed, V. A. Turchenko, A. V Trukhanov, S. V Trukhanov, “A-Site Cation Size Effect on Structure and Magnetic Properties of

$Sm(Eu,Gd)Cr_{0.2}Mn_{0.2}Fe_{0.2}Co_{0.2}Ni_{0.2}O_3$ High-Entropy Solid Solutions" *Nanomaterials* **2022**, *12*, 36.

[29] K. Yazhou, Y. Zhiren, "Synthesis , structure and electrochemical properties of Al doped high entropy perovskite $Li_x(LiLaCaSrBa)Ti_{1-x}Al_xO_3$" *Ceram. Int.* **2022**, *48*, 5035–5039.

[30] Z. Liu, S. Xu, T. Li, B. Xie, K. Guo, J. Lu, "Microstructure and ferroelectric properties of high-entropy perovskite oxides with A-site disorder" *Ceram. Int.* **2021**, *47*, 33039–33046.

[31] F. Shi, G. Liu, "Spin-glass behavior and magnetocaloric properties of high-entropy perovskite oxides" *Appl. Phys. Lett.* **2025**, *120*, 082404.

[32] S. Zhou, T. Ouyang, J. Li, "Dielectric temperature stability and energy storage performance of NBT-based ceramics by introducing high-entropy oxide" *J. Am. Ceram. Soc.* **2022**, *105*, 4796–4804.

[33] A. Kumar, D. Dragoe, D. Berardan, N. Dragoe, "Thermoelectric properties of high-entropy rare-earth cobaltates" *J. Mater.* **2023**, *9*, 191–196.

[34] J. Fang, T. Wang, K. Li, Y. Li, W. Gong, "Energy storage properties of Mn-modified $(Na_{0.2}Bi_{0.2}Ca_{0.2}Sr_{0.2}Ba_{0.2})TiO_3$ high-entropy relaxor-ferroelectric ceramics" *Results Phys.* **2022**, *38*, 105617.

[35] Z. Lou, P. Zhang, J. Zhu, L. Gong, J. Xu, Q. Chen, M. J. Reece, H. Yan, F. Gao, "A novel high-entropy perovskite ceramics $Sr_{0.9}La_{0.1}(Zr_{0.25}Sn_{0.25}Ti_{0.25}Hf_{0.25})O_3$ with low thermal conductivity and high Seebeck coefficient" *J. Eur. Ceram. Soc.* **2022**, *42*, 3480–3488.

[36] Q. Wang, Q. Zhang, G. Wang, Y. Zhang, M. Xia, "High-entropy $La(Fe_{0.2}Co_{0.2}Ni_{0.2}Cr_{0.2}Mn_{0.2})O_3$ ceramic exhibiting high emissivity and low thermal conductivity" *Am. Ceram. Soc.* **2022**, *19*, 2963–2966.

[37] C. Zhou, X. Zhang, S. Li, J. Yan, X. Qi, "Dielectric and energy storage properties of (La,Li $)_x[(Bi,Na)BaSrCa]_{1-x}TiO_3$ high-entropy perovskite ceramics" *Ceram. Int.* **2022**, *48*, 24268–24275.

[38] J. Guo, W. Xiao, X. Zhang, J. Zhang, J. Wang, G. Zhang, "Achieving Excellent Energy Storage Properties in Fine-Grain High-Entropy Relaxor Ferroelectric

Ceramics” *Adv. Electron. Mater.* **2022**, *8*, 2200503.

[39] Z. Shi, J. Zhang, J. Wei, X. Hou, S. Cao, S. Tong, S. Liu, X. Li, Y. Zhang, “A-site deficiency improved the thermoelectric performance of high-entropy perovskite manganite-based ceramics” *J. Mater. Chem. C* **2022**, *10*, 15582–15592.

[40] V. E. Zhivulin, E. A. Trofimov, S. A. Gudkova, E. Al., “Impact of the A-site rare-earth ions (Ln^{3+} – Sm^{3+}, Eu^{3+}, Gd^{3+}) on structure and electrical properties of the high entropy $LnCr_{0.2}Mn_{0.2}Fe_{0.2}Co_{0.2}Ni_{0.2}O_3$ perovskites” *Ceram. Int.* **2022**, *48*, 9239–9247.

[41] Z. Li, Z. Chen, J. Xu, “Enhanced energy storage performance of $BaTi_{0.97}Ca_{0.03}O_{2.97}$-based ceramics by doping high-entropy perovskite oxide” *J. Alloys Compd.* **2022**, *922*, 166179.

[42] Y. Yang, H. Li, B. Duan, Q. Feng, C. Li, X. Lu, G. Chen, C. Li, “A novel high entropy perovskite oxide with co-substitution in A and B sites $(Ca_{1/3}Sr_{1/3}Ba_{1/3})(Y_{1/4}Zr_{1/2}Nb_{1/4})O_3$ design, synthesis and structural characterization” *Ceram. Int.* **2023**, *49*, 7920–7926.

[43] W. Xiong, H. Zhang, Z. Hu, M. J. Reece, H. Yan, “Low thermal conductivity in A-site high entropy perovskite relaxor ferroelectric” *Appl. Phys. Lett.* **2022**, *121*, 112901.

[44] Z. Chen, J. Wu, Z. Chen, H. Yang, K. Zou, X. Zhao, “Entropy Enhanced Perovskite Oxide Ceramic for Efficient Electrochemical Reduction of Oxygen to Hydrogen Peroxide Angewandte” *Angew. Chemie - Int. Ed.* **2022**, *61*, e202200086.

[45] Z. Lou, X. Xu, P. Zhang, L. Gong, Q. Chen, J. Xu, A. Rydosz, F. Gao, “Microstructure and dielectric properties of high- entropy $Sr_{0.9}La_{0.1}MeO_3$ (Me: Zr, Sn, Ti, Hf, Mn, Nb) perovskite ceramics” *J. Mater. Res. Technol.* **2022**, *21*, 850–858.

[46] Y. Chen, R. Li, Y. Zhang, Y. Long, N. Liu, H. Xia, “Preparation and dielectric properties of lead-free perovskite-structured high-entropy ceramics of $(La_{0.25}Sr_{0.25}Ba_{0.25}Na_{0.25})(Ti_{0.5}Me_{0.5})O_{3-\delta}$ (Me=Sn, Zr, Hf) via doping at both A and B sites” *Ceram. Int.* **2023**, *49*, 1038–1047.

[47] T. Wang, Y. Li, X. Zhang, D. Zhang, W. Gong, “Simultaneous excellent energy storage density and efficiency under applied low electric field for high entropy relaxor ferroelectric ceramics” *Mater. Res. Bull.* **2023**, *157*, 112024.

[48] L. Chen, S. Deng, H. Liu, E. Al, “Giant energy-storage density with ultrahigh

efficiency in lead-free relaxors via high-entropy design" *Nat. Commun.* **2022**, *13*, 3089.

[49] K. Zielinska, J. Dabrowa, M. Zajusz, M. Kozusznik, K. ´Swierczek, "Novel OER/ORR Electrocatalysts Based on High-Entropy Perovskites with 3d/4d/5d Dopants" *Catalysts* **2026**, *16*, 192.

[50] R. A. Shishkin, A. P. Tyutyunnik, V. A. Bykov, I. O. Yurev, "Structure and thermal properties of high-entropy perovskites $SrNi_{0.2}Nb_{0.2}W_{0.2}Ti_{0.2}M_{0.2}O_3$, where M = Fe, Mn" *J. Alloys Compd.* **2025**, *1022*, 179797.

[51] M. M. and K. ´Swierczek Juliusz Da˛brow, Anna Olszewska, Andreas Falkenstein, Christian Schwab, Maria Szymczak, Marek Zajusz, Maciej Mo´zdzierz, Andrzej Mikuła, Klaudia Zieli´nska, Katarzyna Berent, Tomasz Czeppe, "An innovative approach to design SOFC air electrode materials: high entropy $La_{1-x}Sr_x(Co,Cr,Fe,Mn,Ni)O_{3-\delta}$ (x = 0, 0.1, 0.2, 0.3) perovskites synthesized by the sol–gel method" *J. Mater. Chem. A* **2020**, *8*, 24455.

[52] X. He, F. Wang, J. Cao, J. Wang, Y. Zheng, M. Zhu, J. Pan, C. Li, "Porous g-C3N4/High-Entropy Perovskite $La(Fe_{0.2}Co_{0.2}Ni_{0.2}Cu_{0.2}Cr_{0.2})O_3$ Nanosheet Heterojunctions for Photocatalytic H_2 Evolution" *ACS Appl. Nano Mater.* **2025**, *8*, 22049−22060.

[53] Y. Liang, B. Luo, H. Dong, D. Wang, "Electronic structure and transport properties of sol-gel-derived high-entropy $Ba(Zr_{0.2}Sn_{0.2}Ti_{0.2}Hf_{0.2}Nb_{0.2})O_3$ thin films" *Ceram. Int.* **2021**, *47*, 20196–20200.

[54] Z. Liu, Z. Tang, Y. Song, G. Yang, W. Qian, "High - Entropy Perovskite Oxide : A New Opportunity for Developing Highly Active and Durable Air Electrode for Reversible Protonic Ceramic Electrochemical Cells" *Nano-Micro Lett.* **2022**, *14*, 1–16.

[55] X. Zhang, L. Xue, F. Yang, Z. Shao, H. Zhang, Z. Zhao, K. Wang, "$(La_{0.2}Y_{0.2}Nd_{0.2}Gd_{0.2}Sr_{0.2})CrO_3$_ A novel conductive porous high-entropy ceramic synthesized by the sol-gel method" *J. Alloys Compd.* **2021**, *863*, 158763.

[56] Y. Shi, N. Ni, Q. Ding, Z. and Xiaofeng, "Tailoring high-temperature stability and electrical conductivity of high entropy lanthanum manganite for solid oxide fuel cell cathodes" *J. Mater. Chem. A* **2022**, *10*, 2256.

[57] C. Jia, B. He, R. Wang, E. Al., "Transparent pn junction in $CuCrO_2/WO_3$ for enhanced photovoltaic response and stability" *Appl. Phys. Lett.* **2024**, *125*, 261901.

[58] J. Luo, Z. Zhang, R. Kang, F. Yang, Z. Hong, S. Xiong, "High-entropy lanthanum-based perovskites for ultra-efficient electromagnetic wave absorption" *J. Alloys Compd.* **2025**, *1042*, 184052.

[59] L. Ma, H.-Y. Niu, C.-J. et al. Jin, "R-P phase high-entropy perovskite $La_{0.5}Sr_{1.5}Mn_{0.2}Fe_{0.2}Ni_{0.2}Cu_{0.2}Co_{0.2}O_4$ achieves complete PMS utilization via multi-pathway activation for efficient water purification" *Chem. Eng. J.* **2025**, 168173.

[60] Z. Li, B. Guan, F. Xia, J. Nie, W. Li, L. Ma, W. Li, L. Zhou, Y. Wang, H. Tian, J. Luo, Y. Chen, M. Frost, K. An, X. Liu, "High-Entropy Perovskite as a High-Performing Chromium-Tolerant Cathode for Solid Oxide Fuel Cells" *ACS Appl. Mater. Interfaces* **2022**, *14*, 24363−24373.

[61] L. Tang, Y. Yang, H. Guo, Y. Wang, M. Wang, Z. Liu, G. Yang, X. Fu, Y. Luo, C. Jiang, Y. Zhao, Z. Shao, Y. Sun, "High Configuration Entropy Activated Lattice Oxygen for O_2 Formation on Perovskite Electrocatalyst" *Adv. Funct. Mater.* **2022**, *32*, 2112157.

[62] A. Bhaskaran, S. A. Singh, S. Roy, "Exploring La-Based High Entropy Perovskites for Syngas Production Via Methane Reforming" *Catal. Letters* **2025**, *155*, 344.

[63] R. Guo, T. He, "High-Entropy Perovskite Electrolyte for Protonic Ceramic Fuel Cells Operating below 600 °C" *ACS Mater. Lett.* **2022**, *4*, 1646−1652.

[64] Z. Zhao, L. Rehder, F. Steinbach, A. Feldhoff, "High-Entropy Perovskites $Pr_{1-x}Sr_x(Cr,Mn,Fe,Co,Ni)O_{3-\delta}$ (x = 0–0.5): Synthesis and Oxygen Permeation Properties" *Membranes (Basel).* **2022**, *12*, 1123.

[65] J. Wang, Z. Cao, X. Zhu, W. Yang, "Improving intermediate-temperature stability of BSCF by constructing high entropy perovskites" *J. Membr. Sci. Lett.* **2022**, *2,* 100026.

[66] J. Liu, C. Ma, L. Wang, E. Al., "Single-phase formation mechanism and dielectric properties of sol-gel-derived $Ba(Ti_{0.2}Zr_{0.2}Sn_{0.2}Hf_{0.2}Ce_{0.2})O_3$ high-entropy ceramics" *J. Mater. Sci. Technol.* **2022**, *130,* 103–111.

[67] K. Chakraborty, S. M. Priya, S. Hati, R. Bal, "Bifunctional $La_xSr_{1-x}(CoCrFeMnNi)O_{3-\delta}$ (x = 0.5;0.7;0.9) high entropy perovskites as potential solid oxide cell air electrode"

Materials Research Bulletin. **2026**, *201*, 114124.

[68] Z. Zhao, H. Chen, H. Xiang, F. Dai, X. Wang, W. Xu, K. Sun, Z. Peng, Y. Zhou, "High-entropy ($Y_{0.2}Nd_{0.2}Sm_{0.2}Eu_{0.2}Er_{0.2}$)$AlO_3$: A promising thermal/environmental barrier material for oxide/oxide composites" *J. Mater. Sci. Technol.* **2020**, *47*, 45–51.

[69] M. Guo, Y. Liu, F. Zhang, F. Cheng, C. Cheng, Y. Miao, F. Gao, J. Yu, "Inactive Al^{3+}-doped La($CoCrFeMnNiAl_x$)1/(5+x)O_3 high-entropy perovskite oxides as high performance supercapacitor electrodes" *J. Adv. Ceram.* **2022**, *11*, 742–753.

[70] G. Meng, Z. Fengnian, M. Yang, E. Al., "Preparation and Electrical Properties of High-entropy La($Co_{0.2}Cr_{0.2}Fe_{0.2}Mn_{0.2}Ni_{0.2}$)$O_3$ Perovskite Ceramics Powder" *J. Inorg. Mater.* **2021**, *36*, 431–435.

[71] T. X. Nguyen, Y. Liao, C. Lin, Y. Su, J. Ting, "Advanced High Entropy Perovskite Oxide Electrocatalyst for Oxygen Evolution Reaction" *Adv. Funct. Mater.* **2021**, *31,* 2101632.

[72] L. Spiridigliozzi, M. Biesuz, V. M. Sglavo, G. D. Agli, "Design , synthesis and formation mechanism of a novel entropy-stabilized perovskite oxide derived from barium cerate / zirconate" *J. Eur. Ceram. Soc.* **2024**, *44*, 2223–2232.

[73] M. Biesuz, S. Fu, J. Dong, A. Jiang, D. Ke, Q. Xu, M. Bortolotti, M. J. Reece, C. Hu, S. Grasso, "High entropy synthesis by reactive spark plasma sintering" *J. Asian Ceram. Soc.* **2019**, *7*, 127–132.

[74] B. Na, K. B. Ca, J. Liu, K. Ren, C. Ma, H. Du, Y. Wang, "Dielectric and energy storage properties of fl ash-sintered high-entropy" *Ceram. Int.* **2020**, *46*, 20576–20581.

[75] K. Wang, B. Ma, T. Li, C. Xie, Z. Sun, D. Liu, J. Liu, L. An, "Fabrication of high-entropy perovskite oxide by reactive flash sintering" *Ceram. Int.* **2020**, *46*, 18358–18361.

[76] T. Parida, A. Karati, S. Mishra, K. Parthiban, K. Parthiban, B. S. Murty, "Low temperature synthesis of multicomponent perovskite by mechanochemical route" *Ceram. Int.* **2022**, *48*, 6385–6392.

[77] and A. Z. Paweł A. Krawczyk, Michał Jurczyszyn, Jakub Pawlak, Wojciech Salamon, Paweł Baran, Angelika Kmita, Łukasz Gondek, Marcin Sikora, Czesław Kapusta, Tomasz Strączek, Jan Wyrwa, "High-Entropy Perovskites as Multifunctional Metal

Oxide Semiconductors: Synthesis and Characterization of $(Gd_{0.2}Nd_{0.2}La_{0.2}Sm_{0.2}Y_{0.2})CoO_3$" *ACS Appl. Electron. Mater.* **2020**, *2*, 3211−3220.

[78] W. Sun, F. Zhang, X. Zhang, T. Shi, J. Li, Y. Bai, C. Wang, Z. Wang, "high-entropy ceramics prepared by hydrothermal method" *Ceram. Int.* **2022**, *48*, 19492–19500.

[79] Y. Ji, X. Zhu, W. Wu, L. Qian, H. Long, "Unraveling the relationship between N_2 selectivity and B-site dispersion in high-entropy perovskites for photocatalytic NO x removal" *J. Catal.* **2026**, *453*, 116560.

[80] A. Sarkar, R. Djenadic, D. Wang, C. Hein, R. Kautenburger, "Rare earth and transition metal based entropy stabilised perovskite type oxides" *J. Eur. Ceram. Soc.* **2018**, *38*, 2318–2327.

[81] L. Su, H. Huyan, A. Sarkar, W. Gao, R. Kruk, H. Hahn, X. Pan, C. Addiego, "Direct observation of elemental fluctuation and oxygen octahedral distortion-dependent charge distribution in high entropy oxides" *Nat. Commun.* **2022**, *13*, 2358.

[82] R. Witte, A. Sarkar, L. Velasco, R. Kruk, R. A. Brand, B. Eggert, K. Ollefs, E. Weschke, H. Wende, H. Hahn, A. Sarkar, L. Velasco, R. Kruk, R. A. Brand, "Magnetic properties of rare-earth and transition metal based perovskite type high entropy oxides" *J. Appl. Phys.* **2020**, *127*, 185109.

[83] L. Eiselt, R. Kruk, H. Hahn, A. Sarkar, "Hole-doped high entropy ferrites: Structure and charge compensation mechanisms in $(Gd_{0.2}La_{0.2}Nd_{0.2}Sm_{0.2}Y_{0.2})_{1-x}Ca_xFeO_3$" *Int. J. ofApplied Ceram. Technol.* **2023**, *20*, 213–223.

[84] R. K. Patel, S. K. Ojha, S. Kumar, A. Saha, P. Mandal, J. W. Freeland, S. Middey, "Epitaxial stabilization of ultra thin films of high entropy perovskite" *Appl. Phys. Lett.* **2020**, *116*, 071601.

[85] A. R. Mazza, E. Skoropata, J. Lapano, J. Zhang, Y. Sharma, B. L. Musico, V. Keppens, Z. Gai, M. J. Brahlek, A. Moreo, D. A. Gilbert, E. Dagotto, T. Z. Ward, "Charge doping effects on magnetic properties of single-crystal $La_{1-x}Sr_x(Cr_{0.2}Mn_{0.2}Fe_{0.2}Co_{0.2}Ni_{0.2})O_3$ $(0 \leq x \leq 0.5)$ high-entropy perovskite oxides" *Phys. Rev. B* **2021**, *104*, 094204.

[86] M. V Kante, M. L. Weber, S. Ni, I. C. G. Van Den Bosch, E. Van Der Minne, L. Heymann, L. J. Falling, N. Gauquelin, M. Tsvetanova, D. M. Cunha, H. Hahn, L. V.

Estrada, G. Koster, F. Gunkel, C. Baeumer, "A High-Entropy Oxide as High-Activity Electrocatalyst for Water Oxidation" *ACS Nano* **2023**, *17*, 5329−5339.

[87] Y. Sharma, M. Lee, K. C. Pitike, K. K. Mishra, Q. Zheng, X. Gao, B. L. Musico, A. R. Mazza, R. S. Katiyar, V. Keppens, M. Brahlek, D. A. Yarotski, R. P. Prasankumar, A. Chen, V. R. Cooper, T. Z. Ward, "High Entropy Oxide Relaxor Ferroelectrics" *ACS Appl. Mater. Interfaces* **2022**, *14*, 11962−11970.

[88] Y. Son, W. Zhu, S. E. Trolier-mckinstry, "Electrocaloric Effect of Perovskite High Entropy Oxide Films" *Adv. Electron. Mater.* **2022**, *8*, 2200352.

[89] Z. J. Corey, P. Lu, G. Zhang, E. Al., "Structural and Optical Properties ofHigh Entropy (La,Lu,Y,Gd,Ce)AlO_3 Perovskite Thin Films" *Adv. Sci.* **2022**, *9*, 2202671.

[90] N. F. Eremeev, S. A. Hanna, E. M. Sadovskaya, A. A. Leonova, O. A. Bulavchenko, A. V Ishchenko, I. P. Prosvirin, V. A. Sadykov, Y. N. Bespalko, "Catalysts for ethanol dry reforming based on high-entropy perovskites" *J. Catal.* **2025**, *445*, 116028.
